# Virtuous victims

**DOI:** 10.1126/sciadv.abg5902

**Published:** 2021-10-13

**Authors:** Jillian J. Jordan, Maryam Kouchaki

**Affiliations:** 1 Harvard Business School, Soldiers Field Road, Boston, MA 02163, USA.; 2 Kellogg School of Management, Northwestern University, 2211 Campus Dr., Evanston, IL 60208, USA.

## Abstract

How do people perceive the moral character of victims? We find, across a range of transgressions, that people frequently see victims of wrongdoing as more moral than nonvictims who have behaved identically. Across 17 experiments (total *n* = 9676), we document this Virtuous Victim effect and explore the mechanisms underlying it. We also find support for the Justice Restoration Hypothesis, which proposes that people see victims as moral because this perception serves to motivate punishment of perpetrators and helping of victims, and people frequently face incentives to enact or encourage these “justice-restorative” actions. Our results validate predictions of this hypothesis and suggest that the Virtuous Victim effect does not merely reflect (i) that victims look good in contrast to perpetrators, (ii) that people are generally inclined to positively evaluate those who have suffered, or (iii) that people hold a genuine belief that victims tend to be people who behave morally.

## Teaser

People see victims as morally virtuous—not because of anything that they have done, but because others have mistreated them.

## INTRODUCTION

People ubiquitously encounter narratives about immoral acts and their victims. We are exposed to victim narratives in our personal and working lives, in the news, and in online and social media, and it has been proposed that victim narratives are an increasingly prevalent staple of contemporary discourse (*1*, *2*). How do these narratives influence our perceptions of victims?

The answer to this question has important societal implications. Perceptions of victims may shape the policy and legal responses that follow wrongdoing toward victims, the ways that victims are treated by members of their social networks, the decisions that victims make about whether to share their stories with others, and the ways that society frames and evaluates moral debates surrounding allegations of victimization.

Research from psychology provides some insight into the ways that people perceive victims of wrongdoing. Research on “victim blaming” has demonstrated that people sometimes see victims as to blame for causing their own victimization (*3*–*6*). Furthermore, a body of research on “moral typecasting” has provided evidence that people can see moral “patients” (i.e., the recipients of moral action), including victims, as less agentic and more passive (*7*–*9*). Here, we investigate a different question surrounding perceptions of victims. In particular, we ask: How do people perceive the moral character of victims?

Moral character is a predominant dimension on which people evaluate others and plays an enormous role in shaping whom we form positive impressions of, choose to affiliate with, and behave prosocially toward (*10*–*14*). Furthermore, asking whether victims are seen as having good or bad moral character is theoretically distinct from asking whether victims are seen as passive (an attribution that is not morally valanced) or receive causal blame (given that it is theoretically possible, for example, to see a victim as morally good while also believing that they contributed, causally, to their victimization). Yet, at first blush, it may seem strange to hypothesize that one’s status as a victim might influence their perceived moral character. After all, both good and bad people can be mistreated: Victims are defined as the recipients of bad treatment, not as actors who behave morally or immorally.

Previous research has established that one’s own moral action is a key determinant of perceived moral character: People who behave morally are seen as morally good, while people who behave immorally are seen as morally bad (*12*–*15*). We also know that other direct attributes of an individual—such as their social group membership (e.g., their race, nationality, religious identity, or political party) (*16*–*20*) or physical attractiveness (*21*, *22*)—may influence their perceived moral character. In contrast, previous research does not provide much direct basis to expect an individual’s perceived moral character to be shaped by the way they are treated by others (or by their status as a victim of others’ immoral action in particular).

Yet, here, we provide evidence that victim narratives can meaningfully shape the perceived morality of victims. We find, across a range of moral transgressions, that people frequently see victims as having elevated moral character—not because of anything that they have done, but because others have mistreated them.

Here, we document this “Virtuous Victim effect” and explore the mechanisms underlying it. We investigate its robustness across potential boundary conditions, considering, for example, which narrative features give rise to the Virtuous Victim effect, and whether the effect is moderated by the victim’s race or gender. We also ask whether the Virtuous Victim effect is specific to victims of immorality (or does it extend to victims of accidental misfortune?) and to moral virtue (or does it extend to positive but nonmoral traits?). In addition, we investigate whether the effect extends beyond perceptions of victims’ moral character to predictions about victims’ moral behavior.

We also evaluate several potential explanations for the Virtuous Victim effect. Ultimately, we find support for a proposal that we term the “Justice Restoration Hypothesis.” According to the Justice Restoration Hypothesis, people see victims as moral because this perception serves to motivate punishment of perpetrators and helping of victims, and people frequently face incentives to enact or encourage these “justice-restorative” actions.

If you see the victim of a transgression as morally good, you might feel especially motivated to help her and to punish on her behalf. Furthermore, there is reason to believe that these are often adaptive responses to wrongdoing (*23*–*28*). Punishment serves to deter future transgressions and thus can be supported by processes like reciprocity (*29*), reputation (*30*–*36*), institutions (*37*, *38*), and cultural group selection (*39*–*42*). For example, in the domain of reputation, punishment can serve as a signal of moral character (*29*–*32*, *36*, *43*) or be supported by social norms (*34*, *35*). Relative to punishment, there has been less research investigating the processes that incentivize victim compensation. However, the same mechanisms that encourage punishment (e.g., reciprocity and reputation) can also encourage prosocial helping (*44*). Moreover, there is some evidence that these mechanisms can encourage helping of victims specifically; for example, helping victims can confer even larger reputational benefits than punishing perpetrators (*45*–*47*). Thus, people might benefit from seeing victims as virtuous, insofar as this perception motivates them to punish perpetrators and/or help victims.

Furthermore, people sometimes face incentives not merely to personally enact these justice-restorative actions but also to encourage others to do the same. Groups often enact collective punishment of norm violators (*48*–*50*) because coordinating can reduce the costs of punishing (*40*). Moreover, when moral disputes occur, people stand to benefit from forming coalitions around their preferred side (*51*). Thus, when wrongdoing occurs, people may face incentives to persuade others that justice-restorative action is merited. These incentives could provide another reason to elevate the morality of victims. If you see a victim as moral, you might be more persuasive at recruiting others to help her and/or punish the perpetrator who harmed her.

In summary, then, people frequently face incentives to enact or encourage justice-restorative action, and it may therefore be beneficial to see victims as virtuous. Moreover, a body of psychological research suggests that self-interest can color our moral judgements (*43*, *52*–*54*). Thus, despite the fact that an individual’s victim status merely reflects how they have been treated by others (and does not provide information about their own moral behavior), we hypothesize that incentives for justice-restorative action might cause people to see victims of wrongdoing as morally good.

As noted above, we also consider other potential explanations for the Virtuous Victim effect. First, victims might benefit from standing in narrative contrast to (morally bad) perpetrators [a “moral contrast effect” (*7*)]. Second, people might feel sympathy for victims and therefore be inclined to evaluate them positively. Third, people might see victims as moral because of a genuine (and perhaps accurate) evaluation that victims tend to be people who behave morally. For example, people might believe, based on their personal experiences, that people who behave morally are easier to exploit and thus more likely to be victimized, or that people who have been victimized are typically disinclined to do the same unto others. However, our results ultimately validate predictions of the Justice Restoration Hypothesis and provide evidence against these alternatives.

Here, we begin by documenting the Virtuous Victim effect and exploring its potential boundaries. Next, we provide support for the Justice Restoration Hypothesis. We then conclude by providing evidence against the aforementioned alternative explanations and, in doing so, further elucidating the effect’s underlying mechanisms.

To these ends, we report analyses from 17 experiments (total *n* = 9676). In all experiments, subjects were recruited online via Amazon Turk, with the exception of one laboratory experiment. We individually preregistered 15 of our experiments; for each of these experiments, we adhered to our preregistered sample size and exclusion criteria. However, given our large number of experiments, we do not sequentially present results for each individual experiment. Rather, we structure our paper around a series of claims and support each claim by analyzing all relevant experiments (including by pooling data across experiments when many are relevant); this approach both facilitates brevity and allows us to provide statistical estimates that are less noisy and more precise. The analyses that we report generally test the same questions as our primary preregistered analyses, with some exceptions. See Materials and Methods for a design overview of each experiment, sections S1 and S5 for more information about our samples and designs, and section S3 for a discussion of preregistered predictions.

## RESULTS

### The Virtuous Victim effect

We begin by documenting the Virtuous Victim effect and exploring its potential boundaries. To do so, we present results from experiments in which subjects read narratives about target characters who were or were not victimized by others.

**Fig. 1.**
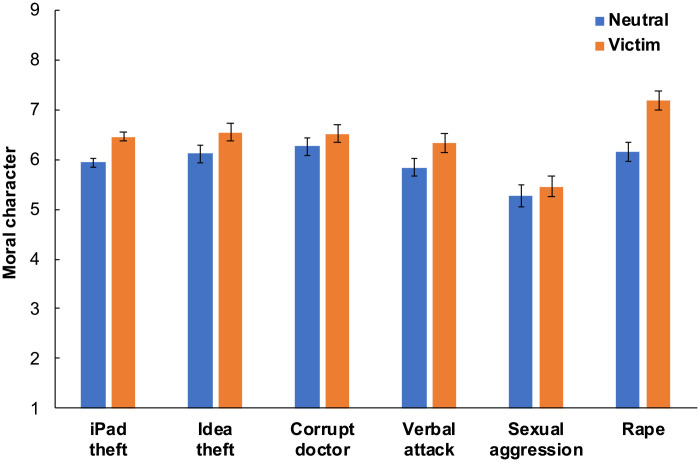
The Virtuous Victim effect across vignettes. We plot the effect of our victim manipulation on ratings of moral character (computed by averaging ratings of morality and trustworthiness) in experiments using our basic design for our iPad theft (experiments 1 to 5; *n* = 1917), idea theft (experiment 6; *n* = 403), corrupt doctor (experiment 7; *n* = 401), verbal attack (experiment 4; *n* = 510), sexual aggression (experiment 4; *n* = 510), and rape (experiment 8; *n* = 437) vignettes. We find evidence that victims are seen as more virtuous than neutral targets (who took the same actions as victims but were not mistreated). Error bars are 95% CIs.

To start, we report results from experiments using our “basic design” (Fig. 1). These experiments manipulated, between subjects, whether subjects were assigned to a “neutral” or “victim” condition. In both conditions, we presented narratives containing identical information about a target character’s behavior. However, in the victim condition, we also informed subjects that another character treated the target immorally. After reading their assigned narratives, subjects evaluated the target’s moral character. Specifically, subjects rated the target’s morality (“How moral of a person is [target]?”) and trustworthiness (“How trustworthy of a person is [target]?”) on 1-to-9 Likert scales (1 = “Not at all”, 3 = “A little bit”, 5 = “Moderately”, 7 = “Quite a lot”, 9 = “Extremely”). Throughout this paper, we treat these questions as two measures of the same underlying construct (i.e., moral character).

In experiments featuring this basic design, subjects did not face or imagine facing any specific incentives to engage in justice-restorative action by punishing the perpetrator or helping the victim. However, we hypothesize that because people frequently face incentives for justice-restorative action in their lives outside of our experiments, they behave by default as if such incentives are present and thus are inclined to elevate the moral character of victims.

We applied our basic design to six vignettes featuring six distinct moral transgressions. Below, for each vignette, we use linear regression to compare the neutral and victim conditions (0 = neutral, 1 = victim) of all experiments featuring our basic design and the relevant vignette. (Some of these experiments also included other conditions, which are not included in these analyses; we note that when introducing each experiment for the first time and reporting its sample size, we report the full *n* across all experimental conditions.) Throughout this paper, for all analyses that aggregate data across multiple experiments, we include experiment dummies in our models. We also note that for all results, we report both unstandardized coefficients (*b*) with their SEs and standardized coefficients (*B*).

Our first vignette, to which we applied our basic design in experiments 1 (*n* = 802), 2 (*n* = 207), 3 (*n* = 803), 4 (*n* = 510), and 5 (*n* = 803) (all preregistered), described the theft of an iPad. In both conditions, subjects learned that the target (e.g., “Sarah”) was a college student and had classmates over to study for an exam. While they were studying, one classmate (e.g., “Gabrielle”) looked something up on Sarah’s iPad. In the neutral condition, the vignette then ended; Sarah was thus a neutral target. In the victim condition, however, the vignette continued to explain that Gabrielle subsequently broke in and stole Sarah’s iPad; Sarah was thus a victim.

Within the basic design conditions across experiments 1 to 5, subjects who read that Sarah’s iPad was stolen perceived Sarah as having elevated moral character. Specifically, relative to neutral targets, theft victims were seen as more moral, *b* = 0.52 [0.39, 0.65], *t* = 7.96, *B* = 0.18, *P* < 0.001, and trustworthy, *b* = 0.50 [0.36, 0.63], *t* = 7.26, *B* = 0.16, *P* < 0.001, *n* = 1917. We also note that in experiment 2, which (unlike all other experiments) was conducted in the physical laboratory with a university subject pool, we found a significant effect of victim status on morality, *b* = 0.52 [0.19, 0.85], *t* = 3.15, *B* = 0.21, *P* = 0.002, but not trustworthiness, *b* = 0.31 [−0.06, 0.68], *t* = 1.64, *B* = 0.11, *P* = 0.102, *n* = 207.

Our second vignette, to which we applied our basic design in experiment 6 (*n* = 802; preregistered), described the theft of an idea. In both conditions, the target worked at an advertising agency and had a good idea for a slogan. In the victim condition only, the target’s manager took undue credit for this idea. Within the basic design conditions of experiment 6, relative to neutral targets, idea theft victims were seen as more moral, *b* = 0.39 [0.13, 0.66], *t* = 2.93, *B* = 0.14, *P* = 0.004, and trustworthy, *b* = 0.48 [0.22, 0.75], *t* = 3.58, *B* = 0.18, *P* < 0.001, *n* = 403.

Our third vignette, to which we applied our basic design in experiment 7 (*n* = 401, preregistered), described a corrupt doctor. In both conditions, the target discussed a medical condition with a doctor. In the victim condition only, to profit, the doctor recommended a drug that he anticipated would (and ultimately did) have adverse effects. We also used our corrupt doctor vignette to investigate whether the Virtuous Victim effect can occur even when subjects have relatively rich background information about the target. Thus, our corrupt doctor vignette (i) described the target’s occupation, marital status, political affiliation, religious background, and hobbies, and (ii) provided information relevant to the target’s moral character (e.g., subjects read that the target can “be a bit self-focused” and “get defensive when criticized” but also “usually comes through when you need a favor”). Relative to neutral targets, victims of the corrupt doctor were seen as significantly more moral, *b* = 0.30 [0.02, 0.59], *t* = 2.12, *B* = 0.11, *P* = 0.034, although not significantly more trustworthy, *b* = 0.20 [−0.06, 0.46], *t* = 1.49, *B* = 0.07, *P* = 0.138, *n* = 401.

Our fourth vignette described a verbal attack. We applied our basic design to this vignette in experiment 4 (in which all subjects evaluated three distinct vignettes). In both conditions, the target attended a party and was approached by a classmate who brought up the fact that he was a gun owner. In the victim condition only, the classmate aggressively verbally attacked the target for his gun ownership. Relative to neutral targets, verbal attack victims were seen as more moral, *b* = 0.52 [0.26, 0.79], *t* = 3.86, *B* = 0.17, *P* < 0.001, and trustworthy, *b* = 0.47 [0.19, 0.76], *t* = 3.32, *B* = 0.15, *P* = 0.001, *n* = 510.

Our fifth vignette, where we likewise applied our basic design in experiment 4, described sexual aggression. In both conditions, the target, a college student, attended a party, began engaging sexually with a man, and then asked him to stop. In the victim condition only, the man nonetheless continued making advances. The vignette did not specify, however, the specific nature of these further advances or whether a sexual assault occurred. We did not find a significant Virtuous Victim effect for this vignette. Relative to neutral targets, victims were not seen as significantly more moral, *b* = 0.19 [−0.13, 0.51], *t* = 1.16, *B* = 0.05, *P* = 0.245, or trustworthy, *b* = 0.18 [−0.13, 0.48], *t* = 1.14, *B* = 0.05, *P* = 0.256, *n* = 510.

Our final vignette, to which we applied our basic design in experiment 8 (*n* = 437; preregistered), described rape. In both conditions, the target was walking home from the grocery store when a male acquaintance offered to help carry her groceries inside, and she accepted. In the victim condition only, once in the target’s apartment, this man locked the door and then raped the target, covering her mouth and threatening her when she tried to scream. The victim condition of this vignette was sourced from the work of Niemi and Young (*5*), and we adapted the vignette to create a neutral condition. We found a strong Virtuous Victim effect for this vignette. Relative to neutral targets, victims were seen as significantly more moral, *b* = 0.99 [0.71, 1.27], *t* = 6.98, *B* = 0.32, *P* < 0.001, and trustworthy, *b* = 1.10 [0.82, 1.38], *t* = 7.64, *B* = 0.34, *P* < 0.001, *n* = 437.

Thus, in five of our six vignettes, subjects saw victims as more moral than nonvictims who behaved identically. We therefore find evidence that victims can be seen as virtuous—not because of actions that they have taken, but simply because others have wronged them.

However, does this Virtuous Victim effect reflect an inference about actions that victims have not taken? In particular, might the effect reflect that subjects are impressed that the victims in our vignettes are not described as lashing out at the perpetrators who wronged them? This explanation struggles to account for the Virtuous Victim effect in (i) our iPad theft vignette (in which the perpetrator is not present when the victim discovers that their iPad is missing), (ii) our corrupt doctor vignette (in which the victim never learns that the doctor was corrupt), and (iii) our rape vignette (in which the victim tries to resist her attacker but is physically overpowered).

We also note that when aggregating across all of our basic design experiments (i.e., all data included in Fig. 1), we very robustly find a positive effect of victim status on both perceived morality, *b* = 0.50 [0.39, 0.60], *t* = 9.50, *B* = 0.16, *P* < 0.001, and trustworthiness, *b* = 0.49 [0.39, 0.59], *t* = 9.28, *B* = 0.15, *P* < 0.001 (*n* = 4178 observations across 3158 unique subjects; SEs are clustered to account for repeated measures across some subjects). Furthermore, in section S2.1, we report and plot the Virtuous Victim effect (i.e., the comparison of our neutral and standard victim conditions) within each individual experiment that featured these conditions and overall in aggregate analyses (see table S3 and fig. S1). We report effects for our morality and trustworthiness dependent variables and also evaluate, for each experiment and in aggregate analyses, whether the magnitude of the effect differed across these two dependent variables. These analyses suggest that the Virtuous Victim effect is robust, and comparable in magnitude, for both morality and trustworthiness. Next, we turn to exploring a few potential boundaries of the Virtuous Victim effect.

#### Which narrative features give rise to the Virtuous Victim effect?

First, we shine the spotlight on two interesting features of the victim narratives featured in our basic design experiments. In these experiments, subjects always read victim narratives that (i) described the perpetrator of the transgression and (ii) were presented in third person (by a presumptively objective narrator). Are these narrative features necessary to produce the Virtuous Victim effect?

Experiment 1 investigated whether the Virtuous Victim effect relies on narrative detail about the perpetrator. To this end, in experiment 1 (which featured our iPad theft vignette), we included an additional condition in which subjects learned that the target’s iPad was stolen but not who stole it or how the theft occurred. Relative to the neutral condition, subjects in this “minimal victim narrative” condition (which is not included in our above analyses or Fig. 1) saw the target as significantly more moral, *b* = 0.37 [0.10, 0.63], *t* = 2.71, *B* = 0.13, *P* = 0.007, but not significantly more trustworthy, *b* = 0.09 [−0.17, 0.36], *t* = 0.67, *B* = 0.03, *P* = 0.500, *n* = 403. Furthermore, relative to minimal narrative victims, standard “full narrative” victims were seen as more moral, *b* = 0.38 [0.11, 0.66], *t* = 2.72, *B* = 0.14, *P* = 0.007, and trustworthy, *b* = 0.52 [0.21, 0.82], *t* = 3.35, *B* = 0.17, *P* = 0.001, *n* = 398. Thus, we find some evidence that the Virtuous Victim effect may occur in the absence of information about the perpetrator, but such detail seems to enhance the effect.

Experiments 5 and 6 investigated whether the Virtuous Victim effect extends to first-person narratives. To this end, we crossed our victim manipulation with a third- versus first-person manipulation, in both experiments 5 (using our iPad vignette) and 6 (using our idea theft vignette). The first-person conditions of these experiments (which, unlike the third-person conditions, are not included in our above basic design analyses or Fig. 1) presented the same information as the third-person conditions, but in the form of the target speaking in first person to a friend. Within the first-person conditions, we observed a significant Virtuous Victim effect for our idea theft vignette but not for our iPad theft vignette (whereas the third-person conditions produced a significant effect for both vignettes). For full results, see section S2.3.

Thus, victims who share their stories can be seen as morally virtuous, but they are not always. We also note that in our iPad theft (but not idea theft) vignette, the victim was not present when the theft occurred, which may have caused subjects to question the credulity of her first-person narrative. As such, our results might reflect that first-person narratives are less likely to inspire the Virtuous Victim effect when there is more room to doubt them.

Together, these analyses shed light on potential boundaries of the Virtuous Victim effect. In particular, they suggest that the Virtuous Victim effect may be especially likely to flow from victim narratives that (i) describe a transgression’s perpetrator and (ii) are presented by a third-person narrator (or perhaps, more generally, a narrator who is unlikely to be doubted).

#### Is the Virtuous Victim effect moderated by target race or gender?

Next, we investigate whether the Virtuous Victim effect is moderated by target race and/or gender. To this end, in experiment 9 (*n* = 904, preregistered), we manipulated victim status via our iPad theft vignette but modified our basic design by providing a photograph of the target in all conditions. We used these photographs, selected from the Chicago Face Database (*55*), to manipulate target race (in particular, by contrasting white versus black targets) and gender.

Using effect coding for each of our three independent variables, we predicted target morality as a function of victim status (−0.5 = neutral; 0.5 = victim), target race (−0.5 = white; 0.5 = black), target gender (−0.5 = male; 0.5 = female), all two-way interactions, and the three-way interaction. We found a main effect of victim status (*b* = 0.44 [0.25, 0.64], *t* = 4.45, *B* = 0.15, *P* < 0.001) and no significant interaction between victim status and target race (*b* = 0.14 [−0.25, 0.53], *t* = 0.72, *B* = 0.02, *P* = 0.475), victim status and target gender (*b* = −0.06 [−0.45, 0.33], *t* = −0.29, *B* = −0.01, *P* = 0.774), or victim status, target race, and target gender (*b* = −0.47 [−1.25, 0.30], *t* = −1.20, *B* = −0.04, *P* = 0.232). Similarly, when using this same approach to predict trustworthiness, we found a main effect of victim status (*b* = 0.43 [0.23, 0.63], *t* = 4.20, *B* = 0.14, *P* < 0.001) that did not significantly interact with race (*b* = 0.30 [−0.10, 0.70], *t* = 1.47, *B* = 0.05, *P* = 0.143), gender (*b* = 0.11[−0.30, 0.51], *t* = 0.52, *B* = 0.02, *P* = 0.604), or race and gender (*b* = −0.48 [−1.28, 0.33], *t* = −1.17, *B* = −0.04, *P* = 0.243), *n* = 904. See section S2.5 for more information about experiment 9, including further analyses of the results and norming data for the selected photographs.

We also note that our broader set of experiments bolsters the claim that the Virtuous Victim effect is not moderated by target gender. Experiment 9 was our only experiment to manipulate target gender via a photograph. However, we manipulated target gender by varying the target’s name and pronouns across a much larger set of experiments. In particular, experiments 1 to 7, 9, and 13 to 15 all both manipulated victim status and manipulated target gender in this way (although note that experiment 4 specifically manipulated gender in the iPad theft but not verbal attack or sexual aggression vignettes). In an aggregate analysis of these experiments (effect coding our independent variables as described above), we find a significant Virtuous Victim effect that is not significantly moderated by target gender, when predicting both morality (main effect: *b* = 0.39 [0.31, 0.47], *t* = 9.41, *B* = 0.13, *P* < 0.001; gender interaction: *b* = −0.10 [−0.26, 0.06], *t* = −1.23, *B* = −0.02, *P* = 0.219) and trustworthiness (main effect: *b* = 0.42 [0.33, 0.50], *t* = 9.75, *B* = 0.14, *P* < 0.001; gender interaction: *b* = −0.01[−0.18, 0.16], *t* = −0.09, *B* = −0.001, *P* = 0.927), *n* = 4829. For more information about the experimental conditions included in this analysis, see section S2.6.

Thus, we find no evidence that the Virtuous Victim effect is moderated by target gender or white versus black race. Of course, the lack of evidence for moderation is not definitive evidence of absence. The confidence intervals (CIs) on the coefficients reported above reveal the upper bounds of interaction effects that are plausible in light of our results and highlight that our null interaction effects are more precisely estimated in the context of our aggregate gender analysis (which draws on 11 different experiments) than experiment 9.

We do note, however, that we find evidence that the Virtuous Victim effect can hold for both black and white victims and for both male and female victims. In particular, when evaluating the Virtuous Victim effect within each race group in experiment 9 (while controlling for target gender), we find significant effects among black targets (morality: *b* = 0.51 [0.24, 0.78], *t* = 3.74, *B* = 0.17, *P* < 0.001; trustworthiness: *b* = 0.58 [0.30, 0.86], *t* = 4.10, *B* = 0.19, *P* < 0.001; *n* = 460). Among white targets, we find a significant effect on morality (*b* = 0.37 [0.09, 0.65], *t* = 2.60, *B* = 0.12, *P* = 0.010) and a marginally significant effect on trustworthiness (*b* = 0.28 [−0.01, 0.57], *t* = 1.92, *B* = 0.09, *P* = 0.056; *n* = 444). In addition, when evaluating the Virtuous Victim effect within each gender group in experiment 9 (while controlling for target race), we find significant effects among female targets (morality: *b* = 0.41 [0.14, 0.68], *t* = 2.99, *B* = 0.14, *P* = 0.003; trustworthiness: *b* = 0.48 [0.22, 0.75], *t* = 3.56, *B* = 0.17, *P* < 0.001; *n* = 449) and among male targets (morality: *b* = 0.48 [0.20, 0.76], *t* = 3.35, *B* = 0.16, *P* = 0.001; trustworthiness: *b* = 0.39 [0.09, 0.69], *B* = 0.12, *t* = 2.54, *P* = 0.011; *n* = 455). Furthermore, when drawing on the data from our aggregate gender analysis, we find significant effects among female targets (morality: *b* = 0.34 [0.23, 0.46], *t* = 5.81, *B* = 0.12, *P* < 0.001; trustworthiness: *b* = 0.42 [0.30, 0.54], *t* = 6.85, *B* = 0.14, *P* < 0.001; *n* = 2361) and among male targets (morality: *b* = 0.45 [0.33, 0.56], *t* = 7.67, *B* = 0.15, *P* < 0.001; trustworthiness: *b* = 0.42 [0.30, 0.54], *t* = 6.98, *B* = 0.14, *P* < 0.001; *n* = 2468). In summary, our results are consistent with the hypothesis that the Virtuous Victim effect can hold regardless of victim gender or (white versus black) race.

### Support for the Justice Restoration Hypothesis

Next, we turn to evaluating potential explanations for this Virtuous Victim effect. We begin by considering our proposed Justice Restoration Hypothesis, which argues that people see victims as morally virtuous because (i) people typically face incentives for justice-restorative action and (ii) seeing victims as virtuous serves to motivate people to help victims and punish perpetrators.

To preview our results, we find two important pieces of support for this hypothesis. First, we find that seeing victims as virtuous does motivate people to help victims and punish perpetrators, as assumed by the Justice Restoration Hypothesis. Second, we find that introducing disincentives to help victims and punish perpetrators eliminates the Virtuous Victim effect, as predicted by the Justice Restoration Hypothesis.

#### Seeing victims as virtuous motivates justice-restorative action

To evaluate the Justice Restoration Hypothesis, we begin by testing its key assumption: that seeing victims as virtuous motivates justice-restorative action. To this end, in experiment 10 (*n* = 598, preregistered), we investigated whether describing victims as morally good motivates people to help them and to punish the perpetrators who have harmed them. Consistent with this prediction, a recent paper showed that people are more willing to help victims who are described as virtuous and that such victims are seen as more deserving of help (*56*). Experiment 10 serves to bolster this evidence and shows that virtuous victims can also motivate punishment of perpetrators.

In experiment 10, we assigned all subjects to the victim condition of our idea theft vignette (in which the target’s idea is stolen). However, before presenting the idea theft vignette, we provided subjects with background information about the target. We used this information to manipulate the target’s morality, describing her as more morally good (e.g., as more likely to donate money, volunteer, and behave prosocially toward friends and family) in the “moral” condition than the “control” condition. After reading this information, subjects learned that the target’s idea was stolen and then rated, on a series of 1-to-9 Likert scales, their willingness to punish the perpetrator and help the target (as well as the target’s morality, as a manipulation check).

**Fig. 2.**
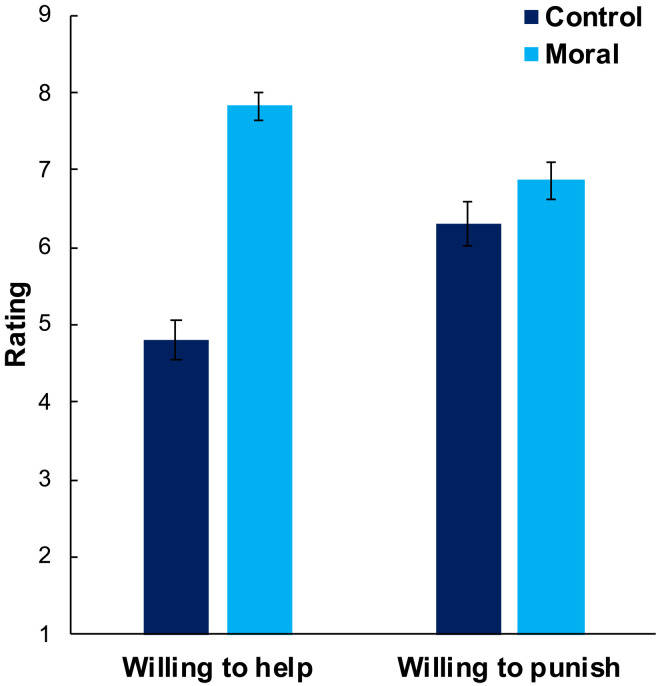
Seeing victims as virtuous motivates justice-restorative action. We plot the effect of our victim morality manipulation on ratings of willingness to help and punish in experiment 10 (*n* = 401). When the victim is described as morally virtuous, subjects are more willing to help her and to punish the perpetrator who harmed her. Error bars are 95% CIs.

Comparing the control and moral conditions (0 = control, 1 = moral), we find that subjects in the moral condition perceived the target as more moral, *b* = 3.55 [3.30, 3.80], *t* = 27.80, *B* = 0.81, *P* < 0.001, and, critically, they were more willing to help her, *b* = 3.02 [2.70, 3.34], *t* = 18.46, *B* = 0.68, *P* < 0.001, and to punish on her behalf, *b* = 0.56 [0.19, 0.93], *t* = 2.99, *B* = 0.15, *P* = 0.003, *n* = 401 (Fig. 2). We thus find evidence, consistent with previous research (*56*), that seeing victims as virtuous serves to motivate justice-restorative action—as assumed by the Justice Restoration Hypothesis.

We also note that experiment 10 included a third condition, in which we described the target as particularly competent (rather than moral). Relative to the control, subjects in this “competent” condition were more willing to help the target, but no more willing to punish the perpetrator. Furthermore, they were less willing than subjects in the moral condition both to help and to punish. Thus, we find some evidence that describing victims as morally virtuous may be especially effective at motivating justice-restorative action. However, this evidence is merely suggestive, because our morality manipulation was (unexpectedly) stronger than our competence manipulation (i.e., it had a larger effect on perceived morality than the competence manipulation had on perceived competence); see section S2.7 for more detail.

In summary, experiment 10 reveals that seeing victims as virtuous motivates justice-restorative action. Next, we ask: Is motivating justice-restorative action actually the function of the Virtuous Victim effect, as proposed by the Justice Restoration Hypothesis? In other words, do people see victims as moral because this perception bolsters motivation to help victims and punish perpetrators? If so, then introducing disincentives for justice-restorative action—such that bolstering these motivations is no longer adaptive—should eliminate the Virtuous Victim effect. We now turn to evaluating this prediction.

#### Introducing disincentives for justice-restorative action eliminates the Virtuous Victim effect

**Fig. 3.**
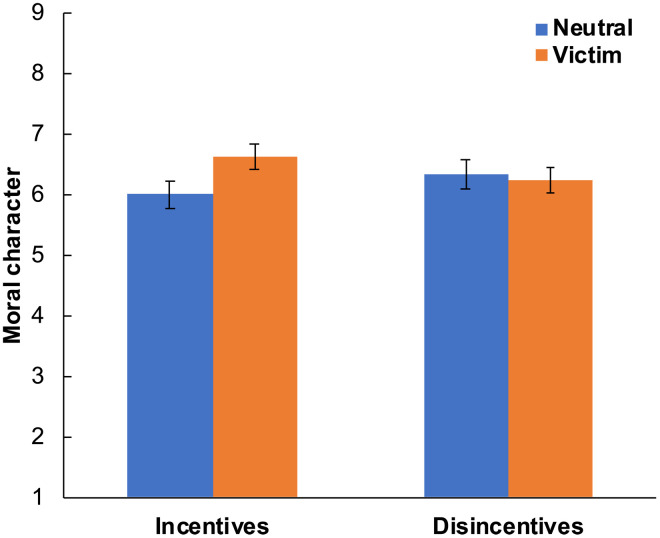
Introducing disincentives for justice-restorative action eliminates the Virtuous Victim effect. We plot ratings of moral character (computed by averaging ratings of morality and trustworthiness) as a function of our disincentives manipulation and victim status in experiment 11a (*n* = 801). We find that when subjects imagine facing disincentives to punish perpetrators and help victims, the Virtuous Victim effect disappears. Error bars are 95% CIs.

In experiment 11a (*n* = 801, preregistered), we investigate whether introducing disincentives to punish perpetrators and help victims causes the Virtuous Victim effect to disappear (Fig. 3). To this end, experiment 11a crossed our standard victim manipulation with a manipulation of disincentives (versus incentives) for justice-restorative action.

Before presenting our idea theft vignette, we asked subjects to imagine working for an advertising agency where ideas are brainstormed in teams, creating the potential for idea theft. In the “incentives” condition, we also asked subjects to imagine (i) believing that idea theft occurs frequently and (ii) having therefore been a vocal critic of the brainstorming system at work. In contrast, in the “disincentives” condition, we asked subjects to imagine (i) believing that idea theft, while unjust, happens rarely (and that the brainstorming system is otherwise very effective) and (ii) having therefore been a vocal defender of the brainstorming system.

In this way, we attempted to manipulate whether subjects, in the scenario they imagined, would face incentives versus disincentives to respond to idea theft with justice-restorative action. We reasoned that engaging in justice-restorative action (i.e., punishing perpetrators and helping victims of idea theft) would call attention to the problem of idea theft, potentially reducing support for the brainstorming system at work. Critically, this outcome was described as consistent with subjects’ imagined goals in the incentives condition but inconsistent with subjects’ imagined goals in the disincentives condition. Furthermore, we reasoned that in the incentives condition, calling attention to idea theft would validate subjects’ imagined public stance (that idea theft was common and the brainstorming system was bad), conferring reputational benefits. In contrast, in the disincentives condition, calling attention to idea theft would undermine subjects’ imagined public stance (that idea theft was rare and the brainstorming system was good), conferring reputational costs. For these reasons, we anticipated that our disincentives condition would convey to subjects that, in the imagined scenario, engaging in justice-restorative action would carry meaningful disadvantages, offsetting the incentives for justice-restorative action that subjects might otherwise face.

After presenting this disincentives manipulation, we presented our idea theft vignette, manipulating whether the target character became a victim of idea theft, and then asked subjects to rate the target’s moral character. We found a significant negative interaction between disincentives (0 = incentives, 1 = disincentives) and victim status (0 = neutral, 1 = victim) on morality, *b* = −0.74 [−1.20, −0.29], *t* = −3.20, *B* = −0.19, *P* = 0.001, and trustworthiness, *b* = −0.73 [−1.18, −0.27], *t* = −3.15, *B* = −0.19, *P* = 0.002, *n* = 801. In the incentives condition, victims were rated as more moral, *b* = 0.58 [0.27, 0.88], *t* = 3.71, *B* = 0.18, *P* < 0.001, and trustworthy, *b* = 0.69 [0.38, 1.00], *t* = 4.39, *B* = 0.21, *P* < 0.001, *n* = 405, than neutral targets. In contrast, in the disincentives condition, there was no significant victim effect on morality, *b* = −0.17 [−0.51, 0.17], *t* = −0.97, *B* = −0.05, *P* = 0.334, or trustworthiness, *b* = −0.04 [−0.38, 0.29], *t* = −0.25, *B* = −0.01, *P* = 0.807, *n* = 396.

Thus, the incentives condition of experiment 11a replicated the Virtuous Victim effect that we typically observe in experiments without an incentives manipulation. In contrast, the disincentives condition of experiment 11a caused the Virtuous Victim effect to disappear. These results are consistent with the hypothesis that people typically face incentives for justice-restorative action and thus behave by default as if such incentives are present. Aligning with this proposal, the Virtuous Victim effect occurs in experiments with no incentives manipulation, and when subjects imagine facing concrete and specific incentives for justice-restorative action (in the incentives condition of experiment 11a). However, when subjects imagine facing concrete and specific disincentives to engage in justice-restorative action (in the disincentives condition of experiment 11a), they cease to elevate victims.

Next, we ask: Did our disincentives manipulation actually influence perceived incentives for justice-restorative action? Or might the disincentives condition have eliminated the Virtuous Victim effect for an unrelated reason? In particular, subjects in the disincentives condition were asked to imagine believing that idea theft, while unjust, is rare, which could conceivably have prevented them from seeing the target of idea theft as a true victim.

To investigate, we conducted experiment 11b (*n* = 399, not preregistered), which served as a post hoc manipulation check for experiment 11a (for a discussion of secondary variables collected in experiment 11a that can also speak to the consequences of our disincentives manipulation, see section S4.1.2). We designed experiment 11b to achieve two key aims. First, we sought to confirm that subjects actually perceive weaker incentives for justice-restorative action in the scenario described by our disincentives (versus incentives) condition. Second, we sought to confirm that taking on the perspective described by our disincentives condition does not prevent people from seeing idea theft as a genuine transgression that creates genuine victims.

To achieve our first aim, we assigned subjects in experiment 11b to evaluate, from an objective third-party perspective, the incentives that subjects in experiment 11a imagined facing. Subjects in experiment 11b thus read about a person named James, who opposed (in the incentives condition) or supported (in the disincentives condition) the brainstorming system. Critically, we described James and his perspective using the same language that we used in experiment 11a to describe subjects’ imagined perspectives. Then, all subjects in experiment 11b (i) read the victim version of our idea theft vignette (in which theft occurs) and (ii) rated James’ incentives to punish the perpetrator and help the victim. Specifically, subjects rated, on 1-to-9 Likert scales, the extent to which these actions would each “help James to achieve his goals” and “be in James’ self interest” (1 = “It would strongly [hurt his goals/be against his interests]”; 5 = “It would be neutral”; 9 = “It would strongly [help his goals/be in his interests]”). For each action, we averaged these two questions together to form a composite measure of perceived incentives.

To achieve our second aim, we asked subjects to take James’ perspective (in particular, by imagining that idea theft occurs either frequently or rarely) and then investigated whether subjects saw the target of idea theft as a victim. Subjects answered the question “To what extent do you think [target] is a victim of wrongdoing?” (1 = “[Target] is not at all a victim”; 5 = “[Target] is somewhat a victim”; 9 = “[Target] is very much a victim”).

We found that, in absolute terms, subjects in the incentives condition both clearly saw the target as a victim (*M* = 8.29, SD = 1.25) and clearly saw James as facing positive incentives to help the victim (*M* = 6.67, SD = 1.75) and punish the perpetrator (*M* = 6.64, SD = 1.55). In contrast, subjects in the disincentives condition also clearly saw the target as a victim (*M* = 8.04, SD = 1.49) but did not see James as facing strong incentives to help (*M* = 5.81, SD = 1.98) or punish (*M* = 4.99, SD = 2.17). Comparing the two conditions (0 = incentives, 1 = disincentives), we find robust differences for incentives to help, *b* = −0.86 [−1.23, −0.49], *t* = −4.55, *B* = −0.22, *P* < 0.001, and to punish, *b* = −1.65 [−2.03, −1.27], *t* = −8.59, *B* = −0.40, *P* < 0.001, as well as a marginally significant difference for victim ratings, *b* = −0.25 [−0.53, 0.02], *t* = −1.81, *B* = −0.09, *P* = 0.071, *n* = 399.

We thus find evidence that our disincentives condition meaningfully weakens perceived incentives for justice-restorative action, as intended. Furthermore, it does not prevent subjects from seeing the target of idea theft as a victim: Victim ratings were very high in both conditions of experiment 11b, despite the marginal difference between them. Together, these results suggest that the disincentives condition really did eliminate the Virtuous Victim effect by reducing incentives for justice-restorative action (and not simply by preventing subjects from seeing idea theft as a genuine transgression that creates genuine victims).

Therefore, experiment 11 supports a key prediction of the Justice Restoration Hypothesis: Introducing disincentives for justice-restorative action causes the Virtuous Victim effect to disappear. Alternative explanations for the Virtuous Victim effect struggle to parsimoniously explain this finding. If the Virtuous Victim effect merely reflected that (i) victims stand in positive contrast to perpetrators, (ii) people feel sympathy for victims and are therefore inclined to evaluate them positively, (iii) people hold a genuine belief that victims tend to behave morally, or (iv) people are impressed that the victims in our vignettes do not lash out at their perpetrators, one would expect the effect to persist in our disincentives condition.

Experiment 11 also provides evidence that the Virtuous Victim effect does not merely reflect a shallow attempt by subjects to communicate their disapproval of the perpetrator. In experiment 11a, before subjects evaluated the target’s morality, they evaluated the morality of her manager (who, in the victim conditions, stole her idea). Thus, subjects in the victim conditions had a direct opportunity to express disapproval of the perpetrator before evaluating the victim—and yet the incentives condition still produced a significant Virtuous Victim effect.

### Evidence against alternative explanations

Together, experiments 10 and 11 support the Justice Restoration Hypothesis and challenge other hypotheses. In the final section of our results, we build on these findings by more directly testing three alternative explanations for the Virtuous Victim effect. In doing so, we both find evidence against these alternatives and further elucidate the effect’s underlying mechanisms. To preview our results, we find that the Virtuous Victim effect (i) does not extend to all parties who stand in contrast to the perpetrator (suggesting that it is not merely a contrast effect), (ii) is specific to victims of immorality (versus accidental misfortune) and to perceptions of moral (versus positive but nonmoral) traits (suggesting that it does not merely reflect a general inclination to positively evaluate anybody who has suffered), and (iii) holds for perceptions of moral character but not predictions of moral behavior (suggesting that it does not merely reflect a genuine belief about the typical conduct of victims).

#### The Virtuous Victim effect is not merely a contrast effect

First, we more directly test the hypothesis that the Virtuous Victim effect merely reflects a contrast effect, whereby victims look good because they stand in contrast to perpetrators. According to this hypothesis, we should expect reading about a transgression to make all nonperpetrator parties look equally good, even if they are not victims. Instead we find evidence that after subjects read about a transgression, they see victims as more moral than nonvictim parties.

Experiment 1 contrasted our standard iPad victim condition (in which Gabrielle stole Sarah’s iPad) with an “other victim” condition (in which Gabrielle, after using Sarah’s iPad to study, stole an iPad from somebody else named Rachel). Subjects then always evaluated Sarah’s moral character. In both the standard and other victim conditions, subjects could contrast Sarah to Gabrielle, but only in the standard victim condition was Sarah a victim. Comparing these conditions (0 = other victim, 1 = standard victim), we find that subjects in the standard victim condition rated Sarah as marginally significantly more moral, *b* = 0.31[−0.03, 0.64], *t* = 1.81, *B* = 0.09, *P* = 0.071, but not significantly more trustworthy, *b* = 0.28 [−0.07, 0.63], *t* = 1.55, *B* = 0.08, *P* = 0.121, *n* = 399.

These results provide some suggestive evidence, but are equivocal. However, although Sarah was not victimized in the other victim condition of experiment 1, because Gabrielle borrowed her iPad before stealing Rachel’s iPad, subjects may have nonetheless seen Sarah as a potential victim. To avoid this perception, in experiment 12 (*n* = 602, preregistered), we modified the other victim condition such that, after borrowing Sarah’s iPad, Gabrielle stole something else from Rachel (specifically, her valuable box of jewelry). (Experiment 12 also differed from experiment 1 by including a hypothetical economic game with the target, discussed further below.) In experiment 12, we found that, relative to the other victim condition (in which Gabrielle stole Rachel’s jewelry), subjects in the standard victim condition (in which Gabrielle stole Sarah’s iPad) rated Sarah as significantly more moral, *b* = 0.43 [0.13, 0.73], *t* = 2.82, *B* = 0.14, *P* = 0.005, and trustworthy, *b* = 0.48 [0.16, 0.80], *t* = 2.96, *B* = 0.15, *P* = 0.003, *n* = 402. Together, we thus find evidence that after subjects read about a transgression, they see the victim as more moral than other parties, suggesting that the Virtuous Victim effect does not merely reflect a contrast effect.

#### The Virtuous Victim effect is specific to morality, suggesting that it is not merely a simple sympathy effect

**Fig. 4.**
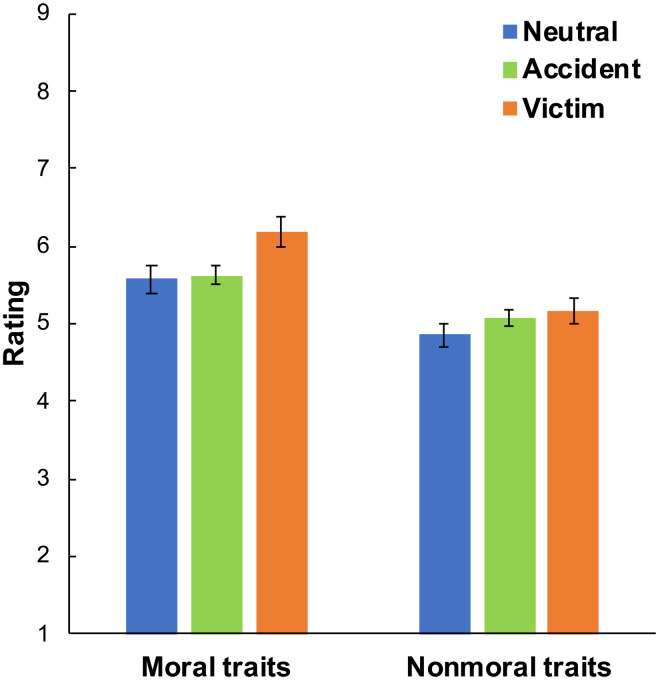
The Virtuous Victim effect is specific to victims of immorality and to moral virtue. We plot average ratings of moral (morality and trustworthiness) and nonmoral (intelligence, athleticism, sociability, and funniness) traits in the neutral, accident (combined earthquake and cat), and victim conditions in experiment 3 (*n* = 803). We find that victims of both immorality and accidental misfortune are seen slightly more positively than neutral targets in the context of nonmoral traits. However, victims of immorality receive an even larger boost in the context of moral traits, while accident victims are seen as no more moral than neutral. Error bars are 95% CIs.

Next, we consider the hypothesis that the Virtuous Victim effect merely reflects that subjects feel sympathy for victims and are thus driven to evaluate them positively. According to this hypothesis, we should expect the Virtuous Victim effect to be quite general: It should extend to all sorts of positive traits and apply to anybody who has suffered. Instead, we find that the Virtuous Victim effect is specific to victims of moral transgressions (i.e., it does not extend to victims of accidental misfortune) and to moral virtue (i.e., it does not extend equally to positive but nonmoral traits) (Fig. 4).

Supporting these claims, in experiment 3, we included our standard victim condition (in which the target’s iPad was stolen) and neutral condition (in which nothing happened to the iPad). We also included two accident conditions, in which the target’s iPad was accidentally knocked off a shelf and destroyed (either because of an earthquake or a stray cat). Furthermore, in addition to rating target morality and trustworthiness, subjects also rated how intelligent, athletic, sociable, and funny the target was. Thus, we measured four positive but nonmoral traits.

The results of experiment 3 suggest that victims of immorality receive a selective boost on moral traits. Comparing victims of immorality to neutral targets (0 = neutral, 1 = immorality victim; *n* = 400), we find that victims are seen more positively on both nonmoral traits (*b* = 0.32 [0.11, 0.53], *t* = 3.00, *B* = 0.15, *P* = 0.003) and moral traits (*b* = 0.61 [0.35, 0.87], *t* = 4.65, *B* = 0.23, *P* < 0.001), but the contrast is significantly more positive for moral traits, *F*(1,1998) = 4.91, *P* = 0.027. [Interactions between victim status and trait type were computed using mixed-model analyses of variance (ANOVAs); see section S1.3 for details.] In contrast, comparing accident victims to neutral targets (0 = neutral, 1 = accident victim; *n* = 605), we find that accident victims are seen more positively on nonmoral traits (*b* = 0.24 [0.06, 0.41], *t* = 2.65, *B* = 0.11, *P* = 0.008) but not moral traits (*b* = 0.05 [−0.16, 0.26], *t* = 0.46, *B* = 0.02, *P* = 0.646), such that the contrast is marginally significantly more positive for nonmoral traits, *F*(1,3023) = 2.97, *P* = 0.085. Moreover, comparing victims of immorality to accident victims (0 = accident victim, 1 = immorality victim; *n* = 601), we find that victims of immorality are not seen significantly more positively on nonmoral traits (*b* = 0.08 [−0.10, 0.26], *t* = 0.89, *B* = 0.04, *P* = 0.376) but are seen more positively on moral traits (*b* = 0.56 [0.35, 0.77], *t* = 5.19, *B* = 0.21, *P* < 0.001), such that the contrast is significantly more positive for moral traits, *F*(1,3003) = 19.70, *P* < 0.001. We note that these analyses aggregate across our two accident conditions, as well as our sets of moral and nonmoral traits; see section S2.2 for more fine-grained analyses, which support the same conclusions.

Thus, subjects in experiment 3 saw victims of immorality, but not victims of accidents, as especially morally virtuous. Other experiments also corroborate these patterns. First, experiment 13 (*n* = 602, preregistered) also provides evidence that victims of immorality are seen as more moral than accident victims. Experiment 13 included our standard iPad theft victim condition and our earthquake condition and measured moral (but not nonmoral) traits. Relative to earthquake victims, theft victims were seen as marginally significantly more moral, *b* = 0.25 [−0.03, 0.54], *t* = 1.75, *B* = 0.09, *P* = 0.081, and significantly more trustworthy, *b* = 0.51 [0.20, 0.81], *t* = 3.28, *B* = 0.16, *P* = 0.001, *n* = 403.

Second, several experiments find that victims of immorality receive a larger boost on moral than nonmoral traits. In addition to experiment 3, experiments 5 to 7 all used our basic design and measured ratings of target morality, trustworthiness, intelligence, and athleticism. We report the effect of our basic victim manipulation on each of these four traits in an aggregate analysis of the standard neutral and immorality victim conditions of these experiments (*n* = 1601).

We find a significant positive effect of victim status on morality, *b* = 0.40 [0.26, 0.54], *t* = 5.75, *B* = 0.14, *P* < 0.001, that holds when controlling for intelligence and athleticism, *b* = 0.35 [0.24, 0.47], *t* = 6.10, *B* = 0.12, *P* < 0.001. Likewise, we find a significant positive effect of victim status on trustworthiness, *b* = 0.41 [0.27, 0.55], *t* = 5.80, *B* = 0.14, *P* < 0.001, that holds when controlling for intelligence and athleticism, *b* = 0.37 [0.25, 0.48], *t* = 6.09, *B* = 0.13, *P* < 0.001. (We do not control for trustworthiness when predicting morality, or vice versa, because both tap the same underlying construct of moral character.) In contrast, we do not find an effect of victim status on intelligence, *b* = 0.04 [−0.09, 0.17], *t* = 0.58, *B* = 0.01, *P* = 0.559, and find a negative effect when controlling for morality, trustworthiness, and athleticism, *b* = −0.22 [−0.33, −0.11], *t* = −3.83, *B* = −0.08, *P* < 0.001. We also find a significant (but relatively smaller) positive effect of victim status on athleticism, *b* = 0.21 [0.07, 0.36], *t* = 2.87, *B* = 0.06, *P* = 0.004, that becomes marginally significant when controlling for morality, trustworthiness, and intelligence, *b* = 0.12 [−0.02, 0.27], *t* = 1.69, *B* = 0.04, *P* = 0.090. Across these four experiments, mixed-model ANOVAs reveal that victim status has a more positive effect on moral traits than it does on intelligence, *F*(1,3197) = 42.09, *P* < 0.001, or athleticism, *F*(1,3197) = 7.22, *P* = 0.007.

In summary, the Virtuous Victim effect does not (i) extend to accidents or (ii) extend equally to positive nonmoral traits. These interesting features of the Virtuous Victim effect suggest that it does not merely reflect a simple sympathy effect, whereby subjects are generally inclined to positively evaluate anybody who has suffered a negative outcome. We note that these results do not rule out the possibility that sympathy is an important part of the psychology surrounding the Virtuous Victim effect. Many of our experiments included sympathy as a secondary variable, and our data are consistent with the possibility that sympathy may be a proximate cause, or consequence, of the Virtuous Victim effect (for analyses and further discussion, see section S4.2.1). Critically, however, any sympathy-based explanation for the Virtuous Victim effect must account for its specificity to morality. This poses a challenge for the simple proposal that people are generally compelled to positively evaluate targets who have suffered.

We also argue that the specificity of the Virtuous Victim effect to morality is compatible with the Justice Restoration Hypothesis. First, consider that the Virtuous Victim effect does not extend equally to positive nonmoral traits. Given this finding, the Justice Restoration Hypothesis should predict that, relative to moral traits, positive nonmoral traits are less effective at motivating justice-restorative action. In other words, we should expect people to be especially motivated to help and punish on behalf of morally virtuous victims. Future research should investigate this interesting possibility, but recall that experiment 10 actually provided some suggestive evidence for it (by contrasting moral versus competent victims).

Second, consider that the Virtuous Victim effect does not extend to victims of accidental misfortune. Given this finding, the Justice Restoration Hypothesis should predict that, relative to cases of immorality, accidents create weaker incentives to help victims and/or punish perpetrators. This is clearly the case with respect to incentives for punishing perpetrators: When accidents occur, there are no perpetrators to punish (or incentives to punish them). Furthermore, although accidents do create victims who may need help, we find empirical evidence that subjects nonetheless perceive weaker incentives to help victims of accidents than victims of immorality.

Specifically, experiment 13 used our iPad vignette to measure perceived (reputation-based) incentives to help different types of targets. Subjects in experiment 13 were assigned to our neutral condition, our theft victim condition, or our earthquake accident condition, and evaluated how good helping the target would look in the eyes of others (before evaluating the target’s moral character). We found that subjects in the theft victim condition reported that helping the target would look better in the eyes of others, both relative to the neutral condition, *b* = 0.74 [0.43, 1.05], *t* = 4.73, *B* = 0.23, *P* < 0.001, *n* = 400, and the accident condition, *b* = 0.81 [0.48, 1.13], *t* = 4.90, *B* = 0.24, *P* < 0.001, *n* = 403. In contrast, relative to neutral, subjects in the accident condition did not report stronger incentives to help, *b* = −0.06 [−0.37, 0.24], *t* = −0.42, *B* = −0.02, *P* = 0.677, *n =* 401.

Thus, while accidents create victims who may need help, subjects perceive relatively stronger (reputation-based) incentives to help victims of immorality. This finding is interesting and, to our knowledge, has not previously been documented. One plausible explanation is that, while all helping behavior signals general prosociality, helping victims of immorality specifically has the added benefit of conveying disapproval of a moral transgression—and thus signaling, like moralistic punishment does (*30*, *31*), that the helper herself is unlikely to transgress.

Regardless, experiment 13 highlights that the Justice Restoration hypothesis is compatible with our finding that the Virtuous Victim effect does not extend to accident victims. The Justice Restoration Hypothesis predicts that the Virtuous Victim effect will specifically occur in contexts where people perceive incentives to help victims and punish perpetrators—and these incentives appear to be weaker in cases of accidents.

#### The Virtuous Victim effect does not extend to predicted behavior, suggesting that it does not merely reflect a genuine belief about the typical conduct of victims

Next we consider the hypothesis that the Virtuous Victim effect merely reflects a genuine belief about the typical conduct of victims. According to this hypothesis, people see victims as morally good because they have evaluated, perhaps accurately, that victims tend to be people who behave morally. If this were true, we would expect subjects to rate victims as more likely to behave virtuously (and/or less likely to behave unethically). Instead, we find that subjects see victims as having elevated moral character (i.e., as being more moral and trustworthy people) but do not expect victims to behave more morally.

**Fig. 5.**
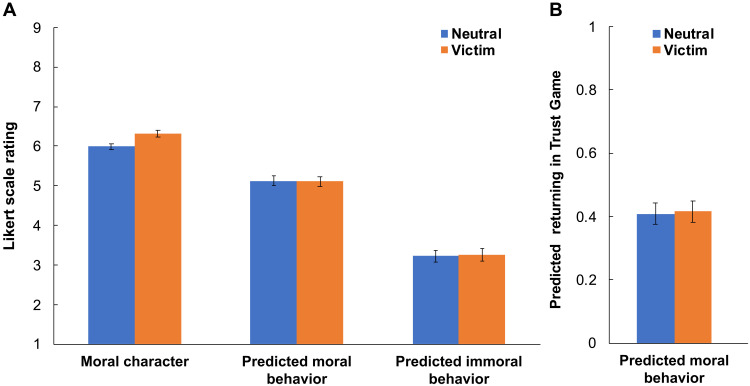
Victims are seen as having elevated moral character but are not expected to behave more morally. We plot the effects of victim status on perceived moral character, predicted moral behavior, and predicted immoral behavior. (**A**) Moral character data (across our set of experiments that measured both moral character and behavior predictions: experiments 1, 7, 12, 14, and 15, *n* = 2008), as well as behavior prediction data from experiments featuring Likert scale measures of predicted behavior (moral behavior data from experiments 1, 7, 14, and 15, *n* = 1608; immoral behavior data from experiments 1, 7, and 14, *n* = 1207). (**B**) Behavior prediction data from experiment 12 (*n* = 400), which measured predicted moral behavior by asking subjects to predict how much money the target would return in a hypothetical economic Trust Game. We find that victims are seen as having elevated moral character but are not expected to behave more morally or less immorally. Error bars are 95% CIs.

Thus far, we have exclusively discussed evaluations of moral character as our key dependent variable. However, experiments 1, 7, 12, 14 (*n* = 403, preregistered), and 15 (*n* = 401, preregistered) all also measured subjects’ predictions about target behavior in moral contexts. These experiments also all used our standard neutral and victim conditions, allowing us to investigate whether the Virtuous Victim effect extends to predicted moral behavior (Fig. 5).

Most of these experiments (specifically, experiments 1, 7, 14, and 15) measured predicted behavior via 1-to-9 Likert scales, making it possible to aggregate data across these experiments. When comparing the standard victim and neutral conditions of these experiments (see Fig. 5A), we find no effect of victim status on predicted moral (*b* = −0.03 [−0.18, 0.12], *t* = −0.38, *B* = −0.01, *P* = 0.707, *n* = 1608) or immoral (*b* = 0.05 [−0.15, 0.25], *t* = 0.51, *B* = 0.01, *P* = 0.613, *n* = 1207) behavior.

In experiment 12, we likewise measured predicted moral behavior. However, rather than using Likert scale measures, we asked subjects to predict how much money the target would return in a hypothetical economic Trust Game (measured as a proportion that could range from 0 to 1). In this experiment (see Fig. 5B), we again found no effect of victim status on predicted moral behavior (*b* = 0.01 [−0.04, 0.06], *t* = 0.29, *B* = 0.01, *P* = 0.775, *n* = 400).

Moreover, when individually analyzing each of our experiments with Likert scale measures of predicted behavior (i.e., each of experiments 1, 7, 14, and 15), we do not find a single significant positive victim effect on predicted moral behavior or a negative victim effect on predicted immoral behavior. These null effects span many measures: Experiment 1 measured predictions of past and future, general and specific, moral and immoral behavior (via eight items). Experiments 7 and 14 measured predictions about four specific moral (e.g., volunteering to tutor) and immoral (e.g., spreading mean gossip) behaviors, and experiment 15 measured predictions about four specific moral behaviors, two of which were habitual (e.g., habitually recycling) and two of which were more active (e.g., surprising one’s mom with a thoughtful gift). Furthermore, experiment 15 provides evidence against the hypothesis that victims are not expected to behave more morally simply because they are perceived to be upset following their victimization. See section S2.9 for more details.

All of our experiments that measured behavior predictions additionally measured moral character ratings, and the order of measures (character ratings versus behavior predictions) varied across experiments. Of note, even when we measured behavior predictions before character ratings (in experiments 12, 14, and 15), although subjects did not expect victims to behave more morally than neutral targets, we did find some evidence that they saw victims as having better character. These three experiments were not included in our aggregated analyses of the basic Virtuous Victim effect (shown in Fig. 1), because they differed from our basic design by measuring behavior predictions before character ratings. Yet, when pooling data across these three experiments, we find that, relative to neutral targets, victims were rated as more moral, *b* = 0.21 [0.05, 0.37], *t* = 2.58, *B* = 0.07, *P* = 0.010, and trustworthy, *b* = 0.29 [0.12, 0.46], *t* = 3.38, *B* = 0.10, *P* = 0.001, *n* = 1204. We do note, however, that these effects are not consistently significant within individual experiments; see section S2.1 (and, in particular, table S3 and fig. S1) for more detail.

Does the dissociation between behavior predictions and character ratings merely reflect that it is too difficult to manipulate behavior predictions? In experiment 16 (*n* = 201, not preregistered), we test this possibility by investigating the effect of a direct morality manipulation on behavior predictions. To directly manipulate morality, we presented subjects with a modified version of the neutral condition of our iPad vignette. In our positively valanced control condition, we modified the vignette by adding that the target “is fun to be around and has a good sense of humor.” In our direct morality condition, we noted these positive traits and called the target “a moral and trustworthy person.” Then, we measured character ratings and then behavior predictions (on a 1-to-9 Likert scale, using the four moral and four immoral behaviors from experiment 14).

This direct morality manipulation (0 = control, 1 = moral) increased ratings of target morality, *b* = 0.45 [0.07, 0.82], *t* = 2.36, *B* = 0.17, *P* = 0.019, and trustworthiness, *b* = 0.54 [0.15, 0.94], *t* = 2.73, *B* = 0.19, *P* = 0.007, *n* = 201. Furthermore, the magnitudes of these effects are comparable to those of our victim manipulation. However, unlike our victim manipulation, our direct morality manipulation also increased predicted moral behavior, *b* = 0.55 [0.15, 0.94], *t* = 2.75, *B* = 0.19, *P* = 0.006—although it had no significant effect on predicted immoral behavior, *b* = −0.28 [−0.72, 0.16], *t* = −1.26, *B* = −0.09, *P* = 0.211, *n* = 201. Thus, we find evidence that the null effects of our victim manipulation on predicted moral behavior do not simply reflect that predicted moral behavior cannot readily be manipulated.

In summary, subjects see victims as having elevated moral character but do not expect them to behave more morally. This interesting dissociation was unexpected. However, critically, it provides evidence against the hypothesis that the Virtuous Victim effect merely reflects a genuine belief that victims tend to be people who behave morally. It also challenges other alternative explanations: If the Virtuous Victim effect were driven by a contrast with the perpetrator or a sympathy-based desire to positively evaluate people who have suffered, it would seem natural for the effect to extend to behavior predictions.

In contrast, we argue that the observed character-behavior dissociation is more readily compatible with the Justice Restoration Hypothesis. The Justice Restoration Hypothesis predicts that people will perceive victims in ways that motivate justice-restorative action. Yet, in our view, it does not make a specific prediction about whether this will merely involve seeing victims as morally good people or will also involve believing that victims are likely to behave morally. Furthermore, insofar as victims do not, in reality, behave any more morally than nonvictims, it may be costly to believe that they do. Thus, elevating the character of victims without expecting them to behave more morally might plausibly function to motivate justice-restorative action while allowing people to avoid the costs of holding inaccurate beliefs. This hypothesis is only speculative and should be investigated in future research. Regardless, however, we argue (i) that the observed character-behavior dissociation provides strong evidence that the Virtuous Victim effect does not reflect a genuine belief about the typical conduct of victims and (ii) that the dissociation is more readily compatible with the Justice Restoration Hypothesis.

## DISCUSSION

Across 17 experiments (total *n* = 9676), we have documented and explored the Virtuous Victim effect. We find that victims are frequently seen as more virtuous than nonvictims—not because of their own behavior, but because others have mistreated them. We observe this effect across a range of moral transgressions and find evidence that it is not moderated by the victim’s (white versus black) race or gender. Humans ubiquitously—and perhaps increasingly (*1*, *2*)—encounter narratives about immoral acts and their victims. By demonstrating that these narratives have the power to confer moral status, our results shed new light on the ways that victims are perceived by society.

We have also explored the boundaries of the Virtuous Victim effect and illuminated the mechanisms that underlie it. For example, we find that the Virtuous Victim effect may be especially likely to flow from victim narratives that describe a transgression’s perpetrator and are presented by a third-person narrator (or perhaps, more generally, a narrator who is unlikely to be doubted). We also find that the effect is specific to victims of immorality (i.e., it does not extend to accident victims) and to moral virtue (i.e., it does not extend equally to positive but nonmoral traits). Furthermore, the effect shapes perceptions of moral character but not predictions about moral behavior.

We have also evaluated several potential explanations for the Virtuous Victim effect. Ultimately, our results provide evidence for the Justice Restoration Hypothesis, which proposes that people see victims as virtuous because this perception serves to motivate punishment of perpetrators and helping of victims, and people frequently face incentives to enact or encourage these justice-restorative actions. We find empirical support for the assumption that seeing victims as virtuous motivates justice-restorative action. We also find, critically, that introducing disincentives for justice-restorative action causes the Virtuous Victim effect to disappear. Moreover, our results provide direct evidence that the Virtuous Victim effect does not merely reflect (i) that victims look good in contrast to perpetrators, (ii) that people are generally inclined to positively evaluate those who have suffered, or (iii) that people hold a genuine belief that victims tend to behave morally.

By supporting the Justice Restoration Hypothesis, our work advances our understanding of how people evaluate the moral character of others. Previous research has established that moral character evaluations are shaped by the direct personal attributes of evaluated individuals, such as their moral or immoral behaviors (*12*–*15*), social group affiliations (*16*–*20*), or physical attractiveness (*21*, *22*). Our results suggest that, because people frequently face incentives to respond to wrongdoing with justice-restorative action, moral character evaluations can also be influenced by whether an individual was the recipient of immoral treatment. In this way, our results contribute to a growing body of evidence from psychology that moral judgements can be colored by self-interested incentives (*43*, *52*–*54*).

By proposing a link between perceptions of victim virtue and justice-restorative action, the Justice Restoration Hypothesis also aligns with theories of “indirect reciprocity.” Such theories posit that people track the reputation status of individuals in their community, and the normative value of a particular action (e.g., helping or stealing from somebody) can depend on the recipient’s reputation standing (*57*, *58*). For example, in some cultures, stealing from somebody in good reputational standing is considered a norm violation, but stealing from somebody in bad reputational standing (e.g., somebody who himself is a thief) is not (*59*). According to this framework, a harmful act is more likely to be viewed by society as a transgression that merits justice-restorative action if the victim is morally virtuous. Thus, it stands to reason that seeing a victim as virtuous would boost motivation for justice-restorative action and that, when we face incentives to enact or encourage justice-restorative action, we would therefore benefit from elevating the victim’s character.

An interesting question for future research is whether incentives for justice-restorative action influence our perceptions of victims in other ways. We find that the Virtuous Victim effect does not extend equally to certain positive nonmoral traits (e.g., intelligence and athleticism). Providing a potential explanation for this pattern, we find some suggestive evidence that describing victims as competent may be less effective at motivating justice-restorative action than describing victims as moral. However, insofar as other traits beyond morality (e.g., helplessness or innocence) are particularly effective at motivating justice-restorative action, we might expect people to elevate victims on those traits.

This proposal may also relate to evidence that people typecast moral patients (i.e., the recipients of moral action), including victims, as less agentic and more passive (*7*–*9*). This phenomenon is distinct from the Virtuous Victim effect (being passive is not the same thing as being moral) and is unlikely to account for our results. If the Virtuous Victim effect simply reflected that victims are seen as passive patients who are incapable of wrongdoing, we would have expected the effect to extend to predicted immoral behavior, but subjects did not rate victims as any less likely to commit immoral acts (e.g., spreading mean gossip). However, future research should investigate whether there may be a psychological link between seeing victims as moral and as passive, insofar as both perceptions could plausibly motivate justice-restorative action.

Another open question is whether the Virtuous Victim effect may ever extend to victims of accidental misfortune. In our experiments, subjects did not see accident victims as more morally virtuous than neutral targets. When viewed through the lens of the Justice Restoration Hypothesis, this pattern makes sense: When accidents occur, there are no perpetrators (and thus no incentives for punishment). In addition, while accidents do create victims who may need help, our subjects did not perceive strong incentives to help them. Notably, our subjects did not expect helping accident victims to look any better than helping neutral targets. However, the accident victims in our experiments suffered relatively minor consequences (the loss of an iPad). When more serious accidents occur (e.g., natural disasters in poor countries), people might plausibly perceive stronger reputational incentives to help, in which case we would expect people to elevate the character of accident victims. Nevertheless, our theory and results also suggest that, holding constant the harm suffered, moral transgressions will create stronger incentives for justice-restorative action than accidents, and victims of immorality will therefore reliably be seen as more virtuous than accident victims.

Future research should also investigate how our results relate to victim blaming. There is ample evidence that people sometimes blame victims for causing their own victimization (*3*–*6*). This observation is not incompatible with our findings: One could conceivably see a victim as morally good and as having contributed, causally, to their victimization. However, moral evaluations of victims may nonetheless correlate interestingly with attributions of causal blame. For example, in some contexts, people face disincentives for justice-restorative action (and thus face pressure not to punish perpetrators and help victims, but rather to excuse wrongdoing and dismiss victims). Our theorizing predicts that in these contexts, people are unlikely to morally elevate victims (and may even derogate their moral character). Indeed, in the disincentives condition of experiment 11a, it was relatively easy to evoke such a context—and consequently eliminate the Virtuous Victim effect—by encouraging subjects to imagine some hypothetical drawbacks of justice-restorative action. Moreover, it seems plausible that in contexts where people perceive disincentives for justice-restorative action, they may also be more likely to attribute causal blame to victims. Future research should test this hypothesis, which is broadly consistent with evidence that motivation (*60*) and ideology (*5*) can influence empathy for victims.

Relatedly, it is interesting to consider why our sexual aggression vignette did not produce a significant Virtuous Victim effect, while our rape vignette produced a strong effect. In the victim condition of our sexual aggression vignette, the target initially participated in a consensual sexual encounter with the perpetrator (who then continued making advances after she asked him to stop). The vignette was also vague: The nature of the continued advances was unclear, and it was thus unclear whether a sexual assault occurred. In contrast, in the victim condition of our rape vignette, the target did not consent to any kind of a sexual encounter, and the vignette described an unambiguous assault. We thus speculate that subjects may have perceived the rape vignette as describing a context that would create greater social consensus that a moral transgression occurred, giving rise to stronger incentives for justice-restorative action and thus a stronger Virtuous Victim effect. Future research should directly test this proposal and more generally investigate when victims of sexual coercion are judged to be morally virtuous.

It is also interesting that, in our experiments, the Virtuous Victim effect was not significantly moderated by target gender or (white versus black) race and extended to female and black victims. When viewed through the lens of the Justice Restoration Hypothesis, these findings suggest that subjects in our experiments perceived incentives to help victims and punish the perpetrators who wronged them, including when the victims in question were female and/or black. However, this perception may not always hold, at least for all subsets of the population. Furthermore, in contexts where people do not perceive incentives to engage in justice-restorative action on behalf of female and/or black victims (or victims from other historically or currently marginalized groups), our theoretical framework suggests that the Virtuous Victim effect may not extend to members of these groups. For example, individuals who perceive incentives to excuse (rather than punish) police violence against black Americans may fail to elevate (and, as described above, perhaps even derogate) the character of such victims. Further research should investigate this important possibility.

Another open question is whether the Virtuous Victim effect occurs across cultures, including in populations that are not “WEIRD” (Western, educated, industrialized, rich, and democratic) (*61*). As just articulated, our theorizing predicts that the generalizability of the Virtuous Victim effect across cultures is likely to depend on the universality of incentives for punishing perpetrators and helping victims. For example, in cultures and contexts where victims are seen as contaminated (*5*) and helping them is not socially rewarded, we predict that the Virtuous Victim effect may disappear (or even reverse).

Further research should also investigate the Virtuous Victim effect outside of the laboratory. A limitation of our work is that we relied on hypothetical vignettes, most of which were presented in third person by a presumptively objective narrator. Thus, future work should explore perceptions of real-world victims, both in contexts where victim narratives are presented by third parties (e.g., news coverage of or gossip about immoral acts and their victims) and victims themselves.

Relatedly, future work should attempt to shed further light on our finding that the Virtuous Victim effect can, but does not always, extend from third- to first-person narratives. We have speculated that this finding may reflect that narrator credibility is crucial for the Virtuous Victim effect. Moreover, while people may be especially likely to question first-person narrators, perceived credibility might also play an important role in shaping evaluations of third-person victim narratives, especially in contexts where narrators seem less objective than they did in our vignettes.

Future research should also investigate the broader societal implications of the Virtuous Victim effect. How does the perception that victims are morally virtuous shape the treatment of victims by society (both in daily life and in domains like policy and law) and the roles that victim narratives play in social debates? Furthermore, what are the implications of the Virtuous Victim effect for the behavior and psychology of victims? For example, when victims are bestowed with moral status, what are the downstream consequences for their moral self-concepts and behavior (*62*, *63*)? In addition, are people aware that being seen as a victim can make them appear moral? Recent research has documented a correlation between the tendency to signal one’s victim status and the tendency to signal one’s moral character (*56*), and our work shows that victim status can itself serve to boost an individual’s perceived moral character. These results raise the question of whether people are motivated to share their victimization to appear virtuous. On the other hand, however, do victims anticipate the pitfalls that may come with personally sharing their first-person narratives? Future work should investigate how these considerations shape the ways that victims choose to come forward with their stories.

In conclusion, we have shown that people frequently see victims of wrongdoing as morally good and provided evidence that this Virtuous Victim effect flows from incentives for justice-restorative action. This work has important implications for the role of victim narratives in society and raises many interesting directions for future research.

## MATERIALS AND METHODS

We recruited subjects online via Amazon Turk and in-lab via a university subject pool. All experiments followed Institutional Review Board guidelines, and informed consent was obtained from all subjects. In all experiments, subjects read vignette(s), evaluated target character(s) on key dependent measure(s), and answered basic demographic questions. Some experiments also included secondary dependent measures (e.g., sympathy for the target), which are analyzed only in the Supplementary Materials. Table 1 provides a design overview of each experiment; see the Supplementary Materials for full design details including secondary variables (sections S1 and S5), as well as supplemental analyses of some of our secondary variables (section S4). We individually preregistered all experiments except experiments 11b and 16; see table S1 for links to all preregistrations, and see section S3 for a discussion of preregistered predictions.

**Table 1.** Overview of experimental designs. We report, for each experiment, the sample source and size, vignette(s) used, key conditions, and key dependent variables. We note that while only experiment 9 manipulated target gender (male versus female) via photographs and as a primary manipulation, experiments 1 to 7, 9, and 13 to 15 also manipulated target gender via names and pronouns. We also note that for experiments 8 and 9, we report the sample sizes for our preregistered primary analyses, which are restricted to subjects who passed a set of attention checks; our unrestricted sample sizes for these experiments are *n* = 503 (experiment 8) and *n* = 999 (experiment 9). (We preregistered attention-based restrictions for experiments 8 and 9 only because these experiments were conducted chronologically last, and during a time in which we had greater concerns about inattention in the Mturk subject pool.)

**Experiment**	**Vignette**	**Key conditions (between subjects)**	**Key dependent measures**
1 (*n* = 802, Mturk)	iPad theft	Neutral; standard victim; other victim; minimal narrative victim	Morality; trustworthiness; predicted past/ future, general/specific, moral/ immoral behavior
2 (*n* = 207, laboratory)	iPad theft	Neutral; standard victim	Morality; trustworthiness
3 (*n* = 803, Mturk)	iPad theft	Neutral; standard victim; accident victim: cat; accident victim: earthquake	Morality; trustworthiness; intelligence; athleticism; funniness; sociability
4 (*n* = 510, Mturk)	iPad theft; verbal attack; sexual aggression (within subjects)	Neutral; standard victim	Morality; trustworthiness
5 (*n* = 803, Mturk)	iPad theft	First-person versus third-person × neutral versus standard victim	Morality; trustworthiness; intelligence; athleticism
6 (*n* = 802, Mturk)	Idea theft	First-person versus third-person × neutral versus standard victim	Morality; trustworthiness; intelligence; athleticism
7 (*n* = 401, Mturk)	Corrupt doctor	Neutral; standard victim	Morality; trustworthiness; intelligence; athleticism; predictions for four moral and four immoral behaviors
8 (*n* = 437, Mturk)	Rape	Neutral; standard victim	Morality; trustworthiness
9 (*n* = 904, Mturk)	iPad theft	Neutral versus standard victim × male vs. female target × white vs. black target	Morality; trustworthiness
10 (*n* = 598, Mturk)	Idea theft	Control victim; moral victim; competent victim	Morality; willingness to help; willingness to punish
11a (*n* = 801, Mturk)	Idea theft	Incentives versus disincentives × neutral versus standard victim	Morality; trustworthiness
11b (*n* = 399, Mturk)	Idea theft	Incentives versus disincentives	Incentives to punish; incentives to help; extent to which victim is a victim
12 (*n* = 602, Mturk)	iPad theft	Neutral; standard victim; other victim	Predicted money returned in hypothetical economic Trust Game; morality; trustworthiness
13 (*n* = 602, Mturk)	iPad theft	Neutral; standard victim; accident victim: earthquake	Reputation-based incentives to help; morality; trustworthiness
14 (*n* = 403, Mturk)	iPad theft	Neutral; standard victim	Predictions for four moral and four immoral behaviors; morality; trustworthiness
15 (*n* = 401, Mturk)	iPad theft	Neutral; standard victim	Predictions for four moral behaviors; morality; trustworthiness
16 (*n* = 201, Mturk)	(No victim narrative; drew from iPad theft)	Positively valanced control; direct morality	Morality; trustworthiness; predictions for four moral and four immoral behaviors

We note that our “basic design” refers to the neutral and standard victim conditions of experiments 1 to 4, 7, and 8, as well as the third-person conditions of experiments 5 and 6. These conditions all used a very similar design, in which subjects were not exposed to other manipulations, and did not complete other measures before rating target moral character (with the exception that, in experiments 3 and 5 to 7, on the same page that subjects rated target moral character, they also rated the target on positive nonmoral traits).

## Supplementary Materials

### This PDF file includes:

Sections S1 to S5 Tables S1 to S11 Figs. S1 to S8
