# Supplementary material for: Virtuous victims
Source: Sci Adv. 2021 Oct 13;7(42):eabg5902. doi: 10.1126/sciadv.abg5902 (PMC8514089; doi:10.1126/sciadv.abg5902)
Supplement: Supplementary file 1 — Sections S1 to S5 Tables S1 to S11 Figs. S1 to S8 [file sciadv.abg5902_sm.pdf]

Science Advances
AAAS

Supplementary Materials for

Virtuous victims

Jillian J. Jordan* and Maryam Kouchaki

*Corresponding author. Email: jijordan@hbs.edu

Published 13 October 2021, *Sci. Adv.* **7**, eabg5902 (2021)
DOI: 10.1126/sciadv.abg5902

This PDF file includes:

Sections S1 to S5
Tables S1 to S11
Figs. S1 to S8

1. Methodological information

1.1. Sample information and exclusion criteria

We recruited subjects online via Amazon Mechanical Turk, or “Mturk” (Experiments 1, 3-16), and in-lab via a university subject pool (Experiment 2). Furthermore, for all of our experiments (as specified in all of our pre-registrations), (i) when we collected duplicate responses from the same IP address or Mturk worker ID, we included only the chronologically first response, and (ii) we excluded responses from subjects who did not complete all the required measures before our demographic questions. Finally, we note that when using Mturk, it is possible to receive slightly more data than requested (if subjects complete the survey, but do not indicate that they have done so on the Mturk platform), so our final simple sizes have the potential to be slightly larger than our target sample sizes. In Table S1, we report sample information (target sample size, total final sample size, final sample size by cell, and age and gender information), as well as links to our pre-registrations, for each experiment.

Experiment	Mean age	Percentage male	Sample size of each cell	Pre-registration link
1 (Target n = 800, final n = 802)	36.43	39.90	Neutral (n = 204), standard victim (n = 199), other victim (n = 200), minimal narrative victim (n = 199)	http://aspredicted.org/blind.php?x=ez3km8
2 (Target n = 200, final n = 207)	20.65	30.92	Neutral (n = 103), standard victim (n = 104)	http://aspredicted.org/blind.php?x=jb7fu7
3 (Target n = 800, n = 803)	35.08	39.85	Neutral (n = 202), standard victim (n = 198), accident victim: cat (n = 201), accident victim: earthquake (n = 202)	http://aspredicted.org/blind.php?x=mn4j6s
4 (Target n = 500, final n = 510)	35.09	40.20	Neutral (n = 255), standard victim (n = 255)	http://aspredicted.org/blind.php?x=fm5kv3
5 (Target n = 800, final n = 803)	35.35	42.47	Neutral first-person (n = 204), neutral third-person (n = 201), standard victim first-person (n = 202), standard victim third-person (n = 196)	http://aspredicted.org/blind.php?x=wn8f8h
6 (Target n = 800, final n = 802)	34.68	41.90	Neutral first-person (n = 196), neutral third-person (n = 198), standard victim first-person (n = 203), standard victim third-person (n = 205)	http://aspredicted.org/blind.php?x=57jf3a
7 (Target n = 400, final n = 401)	35.85	45.89	Neutral (n = 199), standard victim (n =202)	http://aspredicted.org/blind.php?x=38yz33
8 (Target n = 500, final n = 503, primary analysis n = 437)	37.65	49.43	Neutral (n = 222), standard victim (n = 215)	https://aspredicted.org/blind.php?x=59h29w
9 (Target n = 1000, final n = 999, primary analysis n = 904)	41.69	50.77	Neutral n = 441 (White male n = 88, White female n = 101, Black male n = 125, Black female n = 127), Standard victim n = 463 (White male n = 134, White female n = 121, Black male n = 108, Black female n = 100)	https://aspredicted.org/blind.php?x=7gh5g9
10 (Target n = 600, final *n* = 598)	40.34	44.15	Control (n = 189), moral (n = 212), competent (n = 197)	https://aspredicted.org/blind.php?x=ca22f3
11a (Target n = 800, final n = 801)	35.50	41.82	Neutral incentives (n = 200), neutral disincentives (n = 201), standard victim incentives (n = 205), standard victim disincentives (n = 195)	http://aspredicted.org/blind.php?x=q4be2q
11b (Target n = 400, *n* = 399)	39.16	55.14	Incentives (n = 180), disincentives (n = 219)	Not pre-registered
12 (Target n = 600, final n = 602)	35.76	40.37	Neutral (n= 200), standard victim (n = 200), other victim (n = 202)	http://aspredicted.org/blind.php?x=dv2ew7
13 (Target n = 600, final n = 602)	34.98	46.18	Neutral (n = 199), standard victim (n = 201), accident victim: earthquake (n = 202)	http://aspredicted.org/blind.php?x=m437zd
14 (Target n = 400, final n = 403)	33.74	44.67	Neutral (n = 201), standard victim (n = 202)	http://aspredicted.org/blind.php?x=ez3xt6
Pre-test for 15 (Target n = 800, final n = 803)	35.71	42.96	Not upset immoral (n = 202), not upset moral (n = 202), upset immoral (n = 201), upset moral (n = 198)	Not pre-registered
15 (Target n = 400, final n = 401)	35.53	45.39	Neutral (n = 200), standard victim (n = 201)	http://aspredicted.org/blind.php?x=8dr6xz
16 (Target n = 200, final n = 201)	35.61	51.24	Positively-valanced control (n = 103), direct morality (n = 98)	Not pre-registered

Table S1. Sample information and pre-registraiton links for each experiment. Note that in Experiments 9, 10, and 11b, sample sizes are relatively less even across cells because we did not use the "evenly present elements" feature on Qualtrics to ensure equal sample sizes across cells. Note also that Experiments 8-9 included three attention checks and, per our pre-registrations, our primary analyses restrict to subjects who passed all three checks. For these experiments, the reported information about age, gender, and the size of each cell comes specifically from our primary analysis sample. Finally, note that Experiments 2, 8 and 9 (but no other experiments) included a "non-binary/other" option in our measure of subject gender; in Experiment 2, no subjects selected this option; in Experiment 8, the gender distribution was 49.43% male, 49.89% female, and .69% non-binary/other; and in Experiment 9, the gender distribution was 50.77% male, 48.78% female, and .44% non-binary/other.

1.2. Overview of experimental methods

In Tables S2a-b, we provide a detailed overview of the design of each of our experiments. For each table, we report the vignette used, key dependent measures (defined as the variables that we analyzed in the main text), secondary dependent measures (defined as the variables that we did not analyze in the main text), and notes about some salient features of the design.

Then, in the "full experimental methods" section at the end of this document, we provide more extensive design information about each of our experiments. Specifically, for each experiment, we provide a design overview, including the full texts of all versions of our vignettes, and report all measures collected (as well as information about the order in which our measures were collected). Additionally, all of our materials, data, and scripts are available online at https://osf.io/ckxb3/.

We note that Table S2b, the "full experimental methods" section of this document, and our OSF page all include information about a pre-test for Experiment 15; however, this, pre-test is not reported in main text Table 1 (or in the study count or total *n* reported in the main text) because it was merely used to select stimuli for Experiment 15 and its data is not included in any main text analyses.

Experiment	Vignette	Conditions (between-subjects)	Key dependent measures (analyzed in main text) *Note: all variables are with reference to target*	Secondary dependent measures (not analyzed in main text) *Note: only some variables are with reference to target, as specified below*	Design notes
1 (*n* = 802, Mturk)	iPad theft	Neutral; standard victim; other victim; minimal narrative victim	Morality; trustworthiness; predicted past/future, general/specific, moral/immoral behavior	Sympathy towards target; exploitability of target (not including trusting item); blame of "other person" (in all conditions besides neutral)	Randomized gender of characters; two-item Belief in Just World (BJW) scale included in demographic survey (at end of experiment) as potential moderator
2 (*n* = 207, lab)	iPad theft	Neutral; standard victim	Morality; trustworthiness	Sympathy towards target; exploitability of target (including trusting item)	Randomized gender of characters; two-item BJW scale included in demographic survey (at end of experiment) as potential moderator
3 (*n* = 803, Mturk)	iPad theft	Neutral; standard victim; accident victim: cat; accident victim: earthquake	Morality; trustworthiness; intelligence; athleticism; funniness; sociability	Sympathy towards target; exploitability of target (including trusting item)	Randomized gender of characters
4 (*n* = 510, Mturk)	iPad theft; verbal attack; sexual aggression (within-subjects)	Neutral; standard victim	Morality; trustworthiness	Sympathy towards target; exploitability of target (not including trusting item); blame of "other person" (in standard victim)	Randomized gender of characters for iPad theft vignette only; before evaluating vignettes (i.e., at start of experiment), presented demographic survey (with two-item BJW scale) and a set of questions assessing ideology (including about guns, sexual harrassment/assault, and general political ideology), as potential moderators
5 (*n* = 803, Mturk)	iPad theft	First-person vs. third-person X Neutral vs. standard victim	Morality; trustworthiness; intelligence; athleticism	Sympathy towards target; exploitability of target (including trusting item)	Randomized gender of characters
6 (*n* = 802, Mturk)	Idea theft	First-person vs. third-person X Neutral vs. standard victim	Morality; trustworthiness; intelligence; athleticism	Sympathy towards target; exploitability of target (including trusting item); evaluation of target as angry, unforgiving, negative, and entitled; subjects' self-reported belief that events described in vignette were true	Randomized gender of characters
7 (*n* = 401, Mturk)	Corrupt doctor	Neutral; standard victim	Morality; trustworthiness; intelligence; athleticism; predictions for four moral and four immoral behaviors	Sympathy towards target; exploitability of target (including trusting item)	Randomized gender of characters; vignette provides morally relevant background information about the target character
8 (*n* = 503, primary analysis *n* = 437, Mturk)	Rape	Neutral; standard victim	Morality; trustworthiness		
9 (*n* = 999, primary analysis *n* = 904, Mturk)	iPad theft	Neutral vs. standard victim X Male vs. female target X White vs. Black target	Morality; trustworthiness		Provided a photograph of target in all conditions (each subject was randomly presented with one of three potential phtoographs corresponding to their race/gender condition)

Table S2a. Design overview for Experiments 1-9.

Experiment	Vignette	Conditions (between-subjects)	Key dependent measures (analyzed in main text) *Note: all variables are with reference to target, unless otherwise specified*	Secondary dependent measures (not analyzed in main text) *Note: only some variables are with reference to target, as specified below*	Design notes
10 (*n* = 598, Mturk)	Idea theft	Control; moral; competent	Morality; willingness to help target; willingness to punish perpetrator	Competence of target	All subjects saw "victim" condition of idea theft vignette
11a (*n* = 801, Mturk)	Idea theft	Incentives vs. disincentives X Neutral vs. standard victim	Morality; trustworthiness	Morality of "other person"; trustworthiness of "other person"; sympathy towards target; reputation-based incentives to help target; willingness to help target; reputation-based incentives to punish "other person"; willingness to punish "other person"; blame of "other person" (in standard victim); anger towards "other person" (in standard victim)	Manipulation of incentives to vs. not to punish employed an imagination exercise that involved a writing task
11b (*n* = 399, Mturk)	Idea theft	Incentives vs. disincentives	Incentives to punish; incentives to help; extent to which victim is a victim		All subjects saw "victim" condition of idea theft vignette
12 (*n* = 602, Mturk)	iPad theft	Neutral; standard victim; other victim	Predicted money returned in hypothetical economic Trust Game; morality; trustworthiness	Money sent in hypothetical economic Trust Game	Experiment featured a hypothetical Trust Game, in which the subject was the first-mover (i.e., Trustor) and the vignette target was the second-mover (i.e., Trustee), and in which subjects completed a set of Trust Game comprehension questions
13 (*n* = 602, Mturk)	iPad theft	Neutral; standard victim; accident victim: earthquake	Reputation-based incentives to help target; morality; trustworthiness	Willingness to help target; Reputation-based incentives to affiliate with target; willingness to affiliate with target; sympathy towards target	Randomized gender of characters
14 (*n* = 403, Mturk)	iPad theft	Neutral; standard victim	Predictions for four moral and four immoral behaviors; morality; trustworthiness	Intelligence of target; athleticism of target; sympathy towards target; exploitability of target (including trusting item)	Randomized gender of characters; modified the "victim" condition of the vignette to avoid stating that the target was upset following their victimization
Pre-test for 15 (*n* = 803, Mturk)	[did not use a standard vignette]	Immoral vs. moral target X Not upset vs. upset target	Predictions for 12 candidate behaviors		Randomized gender of characters; each subject evaluated a random set of 6 of the 12 candidate behaviors
15 (*n* = 401, Mturk)	iPad theft	Neutral; standard victim	Predictions for four moral behaviors; morality; trustworthiness	Upsetness of target	Randomized gender of characters; used pre-test to select behaviors for behavior predictions DVs. Specifically, two of the moral behaviors were pre-tested *to* be seen as less likely among upset actors, and two were pre-tested *not* to be
16 (*n* = 201, Mturk)	[no victim narrative; drew from iPad theft]	Positively-valanced control; direct morality	Morality; trustworthiness; predictions for four moral and four immoral behaviors	Intelligence of target; athleticism of target	Randomized gender of characters

Table S2b. Design overview for Experiments 10-16.

1.3. Statistical analysis approach

In general, our statistical analyses use linear regression. Whenever we aggregate data across experiments, we include experiment dummies in our statistical models. In Experiment 4, subjects evaluated three different vignettes and thus we have three sets of observations for each subject; in analyses that that include all of these observations (i.e., because they simultaneously analyze more than just one vignette from Experiment 4), our regressions cluster standard error on subject to account for the repeated observations. Additionally, in our analyses of Experiment 3, as well as an aggregate analysis of Experiments 3, 5-7, we compare the effects of our manipulations on ratings of the target on different traits. In these analyses, we use mixed-model ANOVAs in which trait type (e.g., moral vs. nonmoral) is a within-subjects factor and condition (as well as experiment, in our aggregate analysis of Experiments 3, 5-7) is a between-subjects factor.

2. Elaboration of main text analyses

2.1 Analyses of the Virtuous Victim effect on morality and trustworthiness, within each individual experiment and overall

In the main text, we claim that the Virtuous Victim effect is robust, and comparable in magnitude, for both our morality and trustworthiness dependent variables. Here, we support this claim by reporting analyses of the Virtuous Victim effect on each of these dependent variables, within each individual experiment and overall.

To this end, in Table S3 (below), we report results from comparisons of our neutral and standard victim conditions (0 = neutral, 1 = standard victim) within each individual experiment that featured these conditions. For each experiment, we report the vignette used, whether the experiment featured our “basic design” (and if not, how the designed differed from our basic design), the sample size, and the effects of our neutral vs. standard victim comparison on morality and trustworthiness. We also report results from an analysis comparing the magnitude of these two effects (i.e., investigating whether the Virtuous Victim effect was significantly different in size for our morality vs. trustworthiness DVs). To conduct these analyses, we shape our data to have one observation per subject-DV. Then, we predict DV ratings as a function of condition (0 = neutral, 1 = standard victim), DV type (0 = trustworthiness, 1 = morality), and their interaction, clustering standard error on subject to account for repeated measures. We then report the interaction coefficient.

We note that for Experiments 5-6, which crossed our “neutral vs. standard victim” manipulation with a “first vs. third person” manipulation, we report the effects from the *third person* conditions. This choice reflects that (i) all of our other experiments featured only third person conditions, and (ii) our third person conditions more reliably produced a Virtuous Victim effect, thus creating a more informative test of whether that effect was stronger for morality vs. trustworthiness. Similarly, for Experiment 11a, which crossed our “neutral vs. standard victim” manipulation with a manipulation of incentives versus disincentives for justice-restorative action, we report the effects from the *incentives* conditions. This choice reflects that we only predicted and observed a Virtuous Victim effect within our incentives conditions. Finally, we note that in Experiment 9, we report results from the comparison of our neutral and standard victim conditions, collapsing across our manipulations of race and gender.

Table S3 also reports, in bold, results from analyses that pool data across our experiments (and include experiment dummies, as in all aggregate analyses in this paper). In particular, we

conduct one set of analyses that pools data across all of our experiments, and one set of analyses that pools data across our basic design experiments only.

Finally, we note that because subjects in Experiment 4 evaluated three different vignettes, we analyze Experiment 4 three times—once for each of the three vignettes. In our pooled analyses (which include Experiment 4), we shape our Experiment 4 data to have one observation per subject-vignette, clustering standard error on subject to account for repeated measures. (And, in our analyses comparing our morality vs. trustworthiness DVs, we shape our Experiment 4 data to have one observation per subject-vignette-DV.)

Experiment	Vignette	Basic design?	Sample size	Morality effect	Trustworthiness effect	Morality vs. trustworthiness comparison
1	iPad theft	Yes	403	b = .74, t = 5.42, p < .001	b = .61, t = 4.05, p < .001	b = .14, t = 1.16, p = .246
2	iPad theft	Yes	207	b = .52, t = 3.15, p = .002	b = .31, t = 1.64, p = .102	b = .21, t = 1.49, p = .137
3	iPad theft	Yes	400	b = .61, t = 4.36, p < .001	b = .61, t = 3.97, p < .001	b = .01, t = .06, p = .948
4	iPad theft	Yes	510	b = .44, t = 3.14, p = .002	b = .52, t = 3.67, p < .001	b = -.08, t = -.88, p = .379
4	Verbal attack	Yes	510	b = .52, t = 3.86, p < .001	b = .47, t = 3.32, p = .001	b = .05, t = .63, p = .531
4	Sexual agression	Yes	510	b = .19, t = 1.16, p = .245	b = .18, t = 1.14, p = .256	b = .01, t = .13, p = .897
5 (third-person conditions only)	iPad theft	Yes	397	b = .29, t = 2.08, p = .038	b = .34, t = 2.45, p = .015	b = -.06, t = -0.49, p = .621
6 (third-person conditions only)	Idea theft	Yes	403	b = .39, t = 2.93, p = .004	b = .48, t = 3.58, p < .001	b = -.09, t = -0.79, p = .429
7	Corrupt doctor	Yes	401	b = .30, t = 2.12, p = .034	b = .20, t = 1.49, p = .138	b = .11, t = 1.06, p = .290
8	Rape	Yes	437	b = .99, t = 6.98, p < .001	b = 1.10, t = 7.64, p < .001	b = -.11, t = -1.31, p = .192
9	iPad theft	No (included photos of target)	904	b = .40, t = 4.11, p < .001	b = .40, t = 3.90, p < .001	b = .01, t = 0.11, p = .911
11a (incentives conditions only)	Idea theft	No (included incentives manipulation)	405	b = .58, t = 3.71, p < .001	b = .69, t = 4.39, p < .001	b = -.11, t = -1.43, p = .153
12	iPad theft	No (measured predicted moral behavior before measuring moral character)	400	b = .35, t = 2.43, p = .015	b = .26, t = 1.73, p = .085	b = .09, t = 0.92, p = .360
13	iPad theft	No (measured incentives/willingness to help/affiliate with target before measuring moral character)	400	b = .32, t = 2.14, p = .033	b = .45, t = 2.86, p = .004	b = -.12, t = -1.28, p = .202
14	iPad theft	No (measured predicted moral behavior before measuring moral character)	403	b = .14, t = .94, p = .349	b = .39, t = 2.35, p = .019	b = -.25, t = -2.37, p = .018
15	iPad theft	No (measured predicted moral behavior before measuring moral character)	401	b = .14, t = 1.10, p = .271	b = .23, t = 1.73, p = .085	b = -.09, t = -0.97, p = .334
**All experiments, pooled**			**7091 obs, 6071 unique subjects**	**b = .43, t = 11.32, p < .001**	**b = .45, t = 11.67, p < .001**	**b = -.02, t = -0.9, p = .370**
**All basic design experiments, pooled**			**4178 obs, 3158 unique subjects**	**b = .50, t = 9.50, p < .001**	**b = .49, t = 9.28, p < .001**	**b = .01, t = 0.23, p = .815**

Table S3. The Virtuous Victim effect on morality and trustworthiness, across individual experiments and in aggregate analyses.

Together, the analyses presented in Table S3 suggest that the Virtuous Victim effect is robust, and comparable in magnitude, for both our morality and trustworthiness DVs. We also visually illustrate these points in Fig. S1, which displays forest plots from random-effects meta-analyses of the effects reported in Table S3, generated using the "metan" function in STATA. We conducted separate analyses for (i) all experiments in Table S3 (Panels A-B) and (ii) the experiments in Table S3 featuring our basic design (Panels C-D). The overall effects are computed via the DerSimonian-Laird (DL) random-effects model.

Finally, we note that the aggregate analyses reported in Table S3 provide relatively strong evidence that the magnitude of the Virtuous Victim effect does not differ meaningfully across our morality versus trustworthiness DVs. For our aggregate analysis of all experiments, the unstandardized coefficient for the interaction between victim status and DV type is $b = -.02$; the 95% confidence interval on this coefficient is [-.07, .03]. For our aggregate analysis of all basic design experiments, the unstandardized interaction coefficient is $b = .01$; the 95% confidence interval is [-.06, .07]. Given that the overall effect of victim status is about $b = .44$ for all experiments and $b = .50$ for our basic design experiments, these CIs suggest that even the upper bounds of plausible interaction effects are relatively small in comparison.

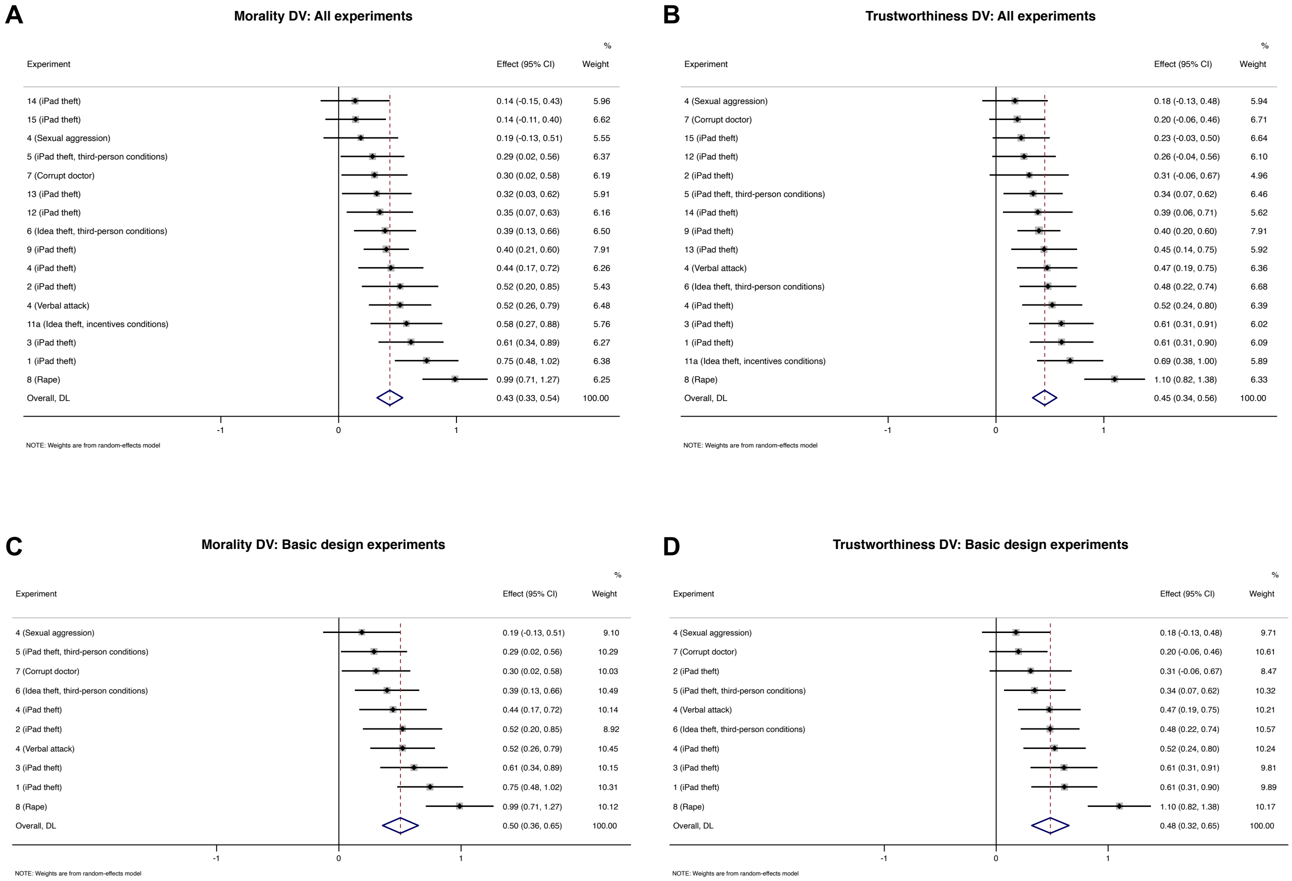

Fig. S1. Forest plots from meta-analyses of the effects reported in Table S3.

2.2. Experiment 3

In main text, we presented analyses of Experiment 3 that aggregated both of our two accident conditions (earthquake and cat), as well as our sets of moral (moral and trustworthy) and nonmoral (intelligent, athletic, funny, and sociable) traits. Here, we elaborate on these analyses. In Fig. S2, we plot ratings of each individual trait in each individual condition, and in Table S4a-b, we present more extensive statistical analyses of these data.

In Table S4a, we present analyses in which we continue to aggregate across our set of moral traits, and across our set of nonmoral traits. For both aggregate moral traits and aggregate nonmoral traits, we report results from linear regressions comparing the victim vs. neutral, accident vs. neutral, and victim vs. accident conditions. Then, we compare the effect of each of these condition contrasts on moral vs. nonmoral traits. To do so, we (i) conduct a mixed-model ANOVA in which the relevant condition contrast is the between-subjects factor and trait type (i.e., moral vs. nonmoral) is the within-subjects factor, and (ii) report the interaction between condition and trait type.

In Table S4b, we present analyses of individual traits. For each trait, we report results from linear regressions comparing the victim vs. neutral, accident vs. neutral, and victim vs. accident conditions.

Throughout Table S4a-b, for all comparisons involving accident conditions, we report results from three tests: one aggregating data from the earthquake and cat conditions, one using data from the earthquake condition only, and one using data from the cat condition only.

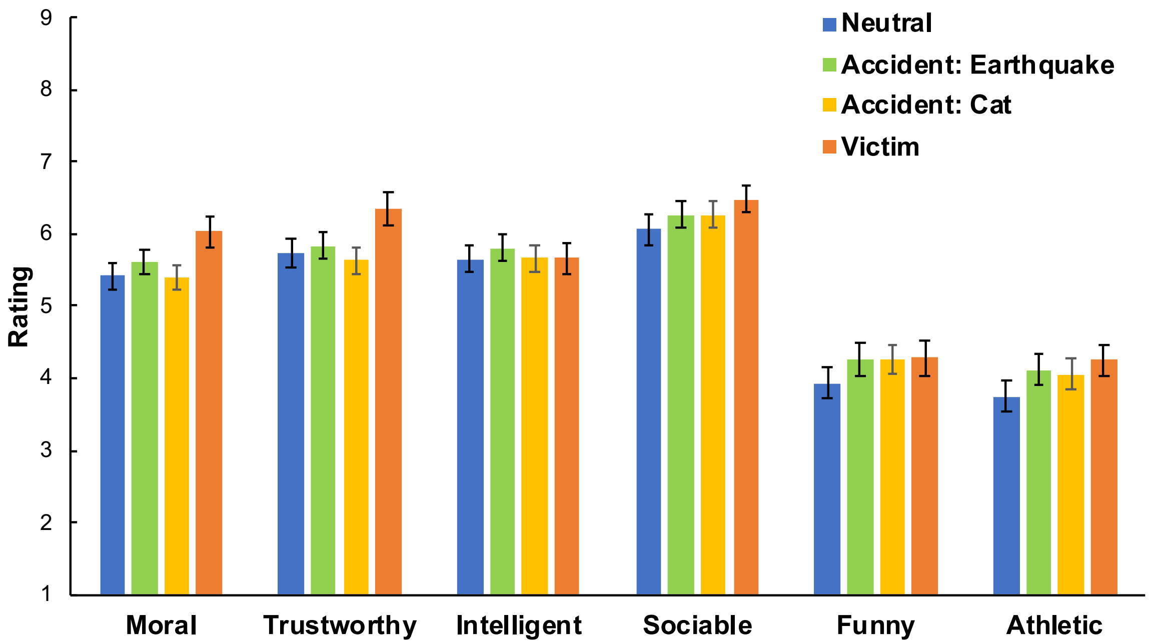

Fig. S2. Trait ratings across conditions in Experiment 3.

	**Victim vs. Neutral (n = 400)**	**Accident vs. Neutral**<br>*Both accident conditions vs. Neutral: n = 605*<br>*Earthquake vs. Neutral: n = 404*<br>*Cat vs. Neutral: n = 403*	**Victim vs. Accident**<br>*Victim vs. Both accident conditions: n = 601*<br>*Victim vs. Earthquake: n = 400*<br>*Victim vs. Cat: n = 399*
Moral traits	Neutral = 5.57, Victim = 6.18, **b = 0.61, t = 4.65, p < .001**	Neutral = 5.57, Both accident conditions = 5.62, **b = 0.05, t = 0.46, p = .646**	Both accident conditions = 5.62, Victim = 6.18, **b = 0.56, t = 5.19, p < .001**
		Neutral = 5.57, Earthquake = 5.73, **b = 0.15, t = 1.23, p = .218**	Earthquake = 5.73, Victim = 6.18, **b = 0.46, t = 3.57, p < .001**
		Neutral = 5.57, Cat = 5.52, **b = -0.06, t = -0.46, p = .643**	Cat = 5.52, Victim = 6.18, **b = 0.67, t = 5.29, p < .001**
Non-moral traits	Neutral = 4.85, Victim = 5.17, **b = 0.32, t = 3.00, p = .003**	Neutral = 4.85, Both accident conditions = 5.09,**b = 0.24, t = 2.65, p = .008**	Both accident conditions = 5.09, Victim = 5.17,**b = 0.08, t = 0.89, p = .376**
		Neutral = 4.85, Earthquake = 5.11, **b = 0.26, t = 2.58, p = .010**	Earthquake = 5.11, Victim = 5.17, **b = 0.06, t = 0.54, p = .591**
		Neutral = 4.85, Cat = 5.06, **b = 0.21, t = 2.05, p = .041**	Cat = 5.06, Victim = 5.17, **b = 0.11, t = 0.98, p = .328**
Moral vs. Non-moral traits	**F = 4.91, p = .027**	Both accident conditions:**F = 2.97, p = .085**	Both accident conditions:**F = 19.70, p < .001**
		Earthquake:**F = 0.69, p = .406**	Earthquake:**F = 9.87, p = .002**
		Cat: **F = 4.50, p = .034**	Cat: **F = 20.10, p < .001**

Table S4a. Analyses of aggregate traits in Experiment 3.

**Trait**	**Victim vs. Neutral (n = 400)**	**Accident vs. Neutral**<br>*Both accident conditions vs. Neutral: n = 605*<br>*Earthquake vs. Neutral: n = 404*<br>*Cat vs. Neutral: n = 403*	**Victim vs. Accident**<br>*Victim vs. Both accident conditions: n = 601*<br>*Victim vs. Earthquake: n = 400*<br>*Victim vs. Cat: n = 399*
Moral	Neutral = 5.41, Victim = 6.03,<br>**b = 0.61, t = 4.36, p < .001**	Neutral = 5.41, Both accident conditions = 5.51,<br>**b = 0.10, t = 0.90, p = .366**	Both accident conditions = 5.51, Victim = 6.03,<br>**b = 0.51, t = 4.51, p < .001**
		Neutral = 5.41, Earthquake = 5.62,<br>**b = 0.21, t = 1.59, p = .112**	Earthquake = 5.62, Victim = 6.03,<br>**b = 0.41, t = 3.00, p = .003**
		Neutral = 5.41, Cat = 5.40,<br>**b = -0.01, t = -0.06, p = .951**	Cat = 5.40, Victim = 6.03,<br>**b = 0.62, t = 4.64, p < .001**
Trust	Neutral = 5.74, Victim = 6.34,<br>**b = 0.61, t = 3.97, p < .001**	Neutral = 5.74, Both accident conditions = 5.73,<br>**b = -0.00, t = -0.03, p = .979**	Both accident conditions = 5.73, Victim = 6.34,<br>**b = 0.61, t = 4.82, p < .001**
		Neutral = 5.74, Earthquake = 5.84,<br>**b = 0.10, t = 0.70, p = .483**	Earthquake = 5.84, Victim = 6.34,<br>**b = 0.51, t = 3.40, p = .001**
		Neutral = 5.74, Cat = 5.63,<br>**b = -0.11, t = -0.75, p = .455**	Cat = 5.63, Victim = 6.34,<br>**b = 0.71, t = 4.76, p < .001**
Intelligent	Neutral = 5.65, Victim = 5.66,<br>**b = 0.01, t = 0.06, p = .955**	Neutral = 5.65, Both accident conditions = 5.73,<br>**b = 0.08, t = 0.65, p = .516**	Both accident conditions = 5.73, Victim = 5.66,<br>**b = -0.07, t = -0.54, p = .588**
		Neutral = 5.65, Earthquake = 5.80,<br>**b = 0.15, t = 1.13, p = .260**	Earthquake = 5.80, Victim = 5.66,<br>**b = -0.14, t = -0.97, p = .334**
		Neutral = 5.65, Cat = 5.66,<br>**b = 0.00, t = 0.02, p = .981**	Cat = 5.66, Victim = 5.66,<br>**b = 0.00, t = 0.03, p = .974**
Sociable	Neutral = 6.06, Victim = 6.48,<br>**b = 0.42, t = 2.93, p = .004**	Neutral = 6.06, Both accident conditions = 6.27,<br>**b = 0.20, t = 1.63, p = .105**	Both accident conditions = 6.27, Victim = 6.48,<br>**b = 0.22, t = 1.84, p = .066**
		Neutral = 6.06, Earthquake = 6.27,<br>**b = 0.20, t = 1.40, p = .163**	Earthquake = 6.27, Victim = 6.48,<br>**b = 0.22, t = 1.59, p = .113**
		Neutral = 6.06, Cat = 6.26,<br>**b = 0.20, t = 1.37, p = .170**	Cat = 6.26, Victim = 6.48,<br>**b = 0.22, t = 1.62, p = .107**
Funny	Neutral = 3.93, Victim = 4.28,<br>**b = 0.35, t = 2.12, p = .035**	Neutral = 3.93, Both accident conditions = 4.26,<br>**b = 0.33, t = 2.42, p = .016**	Both accident conditions = 4.26, Victim = 4.28,<br>**b = 0.02, t = 0.14, p = .887**
		Neutral = 3.93, Earthquake = 4.25,<br>**b = 0.32, t = 2.01, p = .045**	Earthquake = 4.25, Victim = 4.28,<br>**b = 0.03, t = 0.15, p = .878**
		Neutral = 3.93, Cat = 4.26,<br>**b = 0.33, t = 2.16, p = .031**	Cat = 4.26, Victim = 4.28,<br>**b = 0.01, t = 0.09, p = .929**
Athletic	Neutral = 3.75, Victim = 4.25,<br>**b = 0.49, t = 3.12, p = .002**	Neutral = 3.75, Both accident conditions = 4.09,<br>**b = 0.34, t = 2.48, p = .014**	Both accident conditions = 4.09, Victim = 4.25,<br>**b = 0.16, t = 1.16, p = .247**
		Neutral = 3.75, Earthquake = 4.12,<br>**b = 0.37, t = 2.34, p = .020**	Earthquake = 4.12, Victim = 4.25,<br>**b = 0.13, t = 0.82, p = .413**
		Neutral = 3.75, Cat = 4.06,<br>**b = 0.31, t = 1.94, p = .053**	Cat = 4.06, Victim = 4.25,<br>**b = 0.19, t = 1.18, p = .237**

Table S4b. Analyses of individual traits in Experiment 3.

These more extensive analyses support our key conclusions from the main text, and reveal that these conclusions hold for each of our two accident conditions.

Looking to nonmoral traits, we find that both victims of immorality and accidental misfortune are seen slightly more positively than neutral targets (although this effect does not extend to intelligence). The magnitude of this boost is similar for victims of immorality vs. accidents, with no significant differences observed for aggregate nonmoral traits, or for any individual nonmoral traits. (We do, however, find that victims of immorality are seen as marginally significantly more sociable than accident victims—which makes some sense, given that sociability might plausibly be seen as somewhat morally relevant).

Looking to moral traits, we find that victims of immorality receive an even larger boost, relative to neutral targets, than they did for nonmoral traits—and this boost holds for both morality and trustworthiness. In contrast, accident victims are rated as no more moral or trustworthy than neutral targets.

Together, we thus find that *immorality* victims receive a selective boost on *moral* traits.

2.3. Experiments 5-6

As described in the main text, Experiments 5 and 6 investigated whether the Virtuous Victim effect extends to first-person narratives. To this end, we crossed our victim manipulation with a third- vs. first-person manipulation, both in Experiment 5 (using our iPad vignette) and Experiment 6 (using our idea theft vignette). Here, we report results from these experiments.

In Experiment 5, we observed the Virtuous Victim effect within the third-person condition (n = 397) for both morality, b = .29 [.02, .56], t = 2.08, B = .10, p = .038, and trustworthiness, b = .34 [.07, .62], t = 2.45, B = .12, p = .015. However, within the first-person condition (n = 406), we found no significant effect for morality, b = .17 [-.12, .46], t = 1.13, B = .06, p = .259, and found a marginally significant *negative* effect for trustworthiness, b = -.28 [-.59, .02], t = -1.85, B = -.09, p = .065. When predicting our DVs as a function of victim status (neutral = 0, victim = 1), narrative type (0 = third-person, 1 = third-person), and their interaction, we did not observe a significant interaction for morality, b = .12 [-.28, .52], t = .59, B = .04, p = .552, but we did for trustworthiness, b = .63 [.22, 1.04], t = 3.02, B = .18, p = .003, n = 803. Thus, in Experiment 5, the Virtuous Victim effect did not extend to first-person narratives.

In Experiment 6, we again observed the Virtuous Victim effect within the third-person condition (n = 403) for both morality, b = .39 [.13, .66], t = 2.93, B = .14, p = .004, and trustworthiness, b = .48 [.22, .75], t = 3.58, B = .18, p < .001. But we also observed the Virtuous Victim effect within the first-person condition (n = 399) for both morality, b = .39 [.08, .70], t = 2.46, B = .12, p = .014, and trustworthiness, b = .36 [.07, .66], t = 2.45, B = .12, p = .015. And we did not observe a significant interaction between victim status and narrative type for either morality, b = .01 [-.40, .41], t = .04, B = .002, p = .970, or trustworthiness, b = .12 [-.27, .51], t = .59, B = .04, p = .554, n = 802. Thus, in Experiment 6, the Virtuous Victim effect did extend to first-person narratives.

2.4. Experiment 8

In the main text, per our pre-registration, we report analyses of Experiment 8 that restrict to subjects who passed three attention checks (n = 437). Here, we demonstrate that the conclusions of Experiment 8 hold in secondary analyses that do not exclude subjects on the basis of attention check performance (n = 503). Indeed, analyses of all subjects reveal that, relative to neutral targets, victims were seen as significantly more moral, b = .97 [.72, 1.23], t = 7.51, B = .32, p < .001, and trustworthy, b = 1.07 [.81, 1.33], t = 8.06, B = .34, p < .001, n = 503. Thus, in analyses of all subjects, we again find a Virtuous Victim effect in Experiment 8.

2.5. Experiment 9

As described in the main text, in Experiment 9 we investigated whether the Virtuous Victim effect is moderated by target race and/or gender. To this end, we manipulated victim status via our iPad theft vignette, but modified our basic design by providing a photograph of the target in all conditions. We used these photographs to manipulate target race (specifically, by contrasting White vs. Black targets) and gender. In particular, each subject was assigned to one race/gender condition (i.e., *White male*, *White female*, *Black male*, or *Black female*) and then was randomly presented with one of three potential target photographs corresponding to their condition.

As described in Section 5.9 of this document, all photographs were selected from the Chicago Face Database (Ma et al., 2015). We selected photographs depicting a closed mouth smile, and of models who were roughly college-aged (given that the iPad theft vignette is about

college students). We also selected photographs that were reasonably well-matched across race/gender conditions on perceived age, attractiveness, and trustworthiness (using norming data from the Chicago Face Database). In Fig. S3, we show the results of Experiment 9 along with example photographs corresponding to each race/gender condition, and in Table S5, we report relevant norming data for each photograph (and each race/gender condition on average).

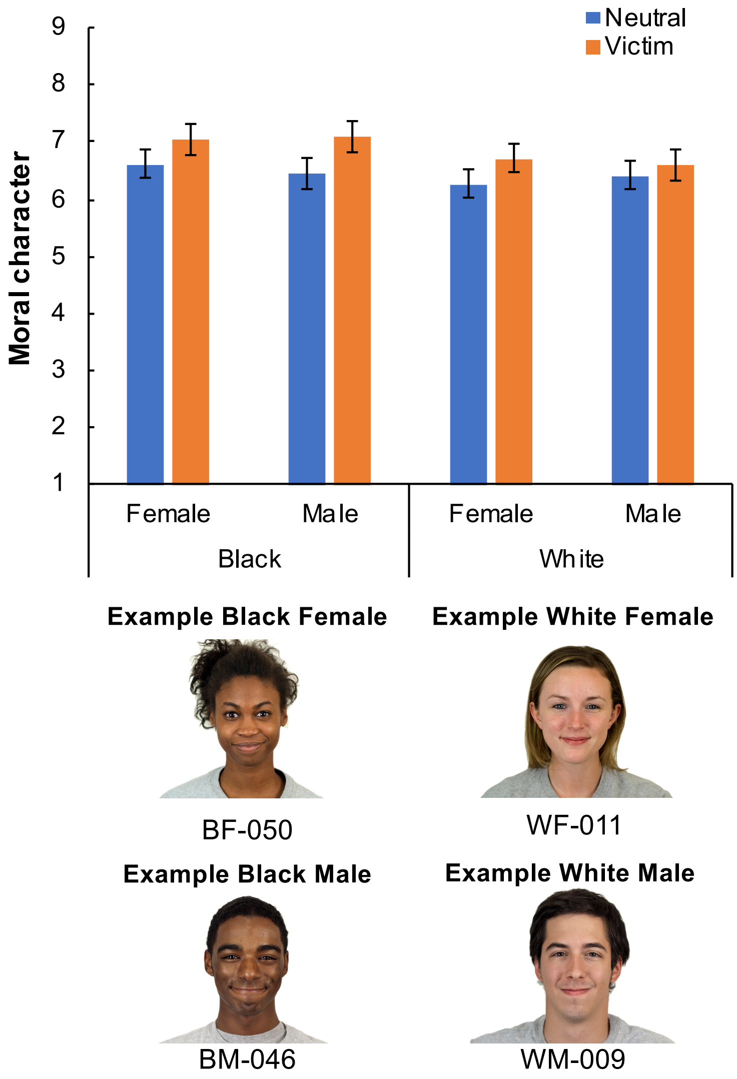

Fig. S3. We plot the results from Experiment 9 (among subjects who passed all attention checks, n = 904), along with the set of target photographs used across race and gender conditions. Photo Credit: The Chicago Face Database, University of Chicago, Center for Decision Research.

Race/gender condition	Model	Perceived age (years)	Percieved attractiveness (1-7 Likert scale)	Perceived trustworthiness (1-7 Likert scale)	Rater N
Black female	BF-028	22.62	3.73	4.08	93
	BF-049	20.83	4.45	4.11	94
	BF-050	22.38	3.79	3.43	96
	**BF Average**	**21.94**	**3.99**	**3.87**	
Black male	BM-026	26.99	3.90	3.67	93
	BM-037	20.89	4.04	3.49	90
	BM-046	22.06	3.77	3.49	89
	**BM Average**	**23.31**	**3.91**	**3.55**	
White female	WF-011	24.04	4.13	3.70	95
	WF-021	25.69	3.93	3.49	91
	WF-031	23.24	4.12	3.73	85
	**WF Average**	**24.32**	**4.06**	**3.64**	
White male	WM-003	23.35	3.68	3.58	83
	WM-009	23.70	4.08	3.51	92
	WM-033	26.59	3.85	3.58	91
	**WM Average**	**24.54**	**3.87**	**3.56**	

Table S5. Norming data from the Chicago Face Database for each of the photographs used in Experiment 9. We note that the labels for each model correspond to the labels used in the Chicago Face Database.

In the main text, per our pre-registration, we report analyses of Experiment 9 that restrict to subjects who passed three attention checks (n = 904). Here, we demonstrate that the conclusions of Experiment 9 hold in secondary analyses that do not exclude subjects on the basis of attention check performance (n = 999). To this end, we repeat our main analysis approach for Experiment 9 by predicting target morality, and trustworthiness, as a function of victim status (-.5 = neutral, .5 = victim), target race (-.5 = White, .5 = Black), target gender (-.5 = male, .5 = female), all two-way interactions, and the three-way interaction.

Predicting morality, we find a main effect of victim status (b = .43 [.24, .61], t = 4.54, B = .14, p < .001), and no significant interaction between victim status and target race (b = .17 [-.20, .54], t = .93, B = .03, p = .355), victim status and target gender (b = -.12 [-.49, .25], t = -.66, B = -.02, p = .509), or victim status, target race, and target gender (b = -.30 [-1.04, .44], t = -.79, B = -.02, p = .432). Predicting trustworthiness, we find a main effect of victim status (b = .42 [.23, .61], t = 4.30, B = .14, p < .001) that did not interact with race (b = .29 [-.10, .67], t = 1.47, B = .05, p = .141), gender (b = .09 [-.29, .48], t = .46, B = .01, p = .645), or race and gender (b = -.34 [-1.11, .43], t = -.87, B = -.03, p = .383), n = 999. Thus, in analyses of all subjects, we again find a Virtuous Victim effect that is not significantly moderated by target race and/or gender.

2.6. Aggregate analysis of target gender

In the main text, we reported an aggregate analysis of our set of experiments that manipulated target gender. Specifically, we reported that, across this set of experiments, (i) the Virtuous Victim effect was not moderated by target gender and (ii) the Virtuous Victim effect held among both male and female targets.

As described in the main text, Experiments 1-7, 9, and 13-15 all both manipulated victim status and manipulated target gender (although note that Experiment 4 specifically manipulated gender in the iPad theft but not verbal attack or sexual aggression vignettes). Within these experiments, our aggregate gender analysis specifically takes data from our "standard set of neutral vs. victim conditions". This label refers to the set of data that we analyze in Fig. S1 and Table S3: the standard victim and neutral conditions of all experiments that included both of these conditions, with the exceptions of (i) the first-person conditions of Experiments 5 and 6, and (ii) the "disincentives" conditions of Experiment 11a. (As described in Section 2.1 of this document, these exceptions reflect that we expected that these conditions might meaningfully change the effects of our standard victim manipulation, and our results supported this expectation.) Note that we also draw on this standard set of neutral vs. victim conditions in our aggregate analyses of two secondary dependent variables (sympathy and exploitability), reported in Section 4.2 of this document.

2.7. Experiment 10

In the main text, we use Experiment 10 to show that when a victim of wrongdoing is described as more morally virtuous, subjects are more willing to help the victim and to punish the perpetrator. Furthermore, our main text also briefly mentions that Experiment 10 included an additional condition, in which we described the target as particularly *competent* (rather than moral). In total, then, Experiment 10 featured three conditions: *control* (in which the target was described as relatively less moral and relatively less competent), *moral* (in which the target was described as relatively more moral and relatively less competent), and *competent* (in which the target was described as relatively less moral and relatively more competent).

Here, we report results from the competent condition. Relative to the neutral condition, subjects in the competent condition perceived the target as more competent, $b = 1.87$ [1.58, 2.16], $t = 12.79$, $B = .55$, $p < .001$, and were more willing to help her, $b = .46$ [.07, .85], $t = 2.31$, $B = .12$, $p = .021$, but were not significantly more willing to punish on her behalf, $b = .08$ [-.32, .48], $t = .39$, $B = .02$, $p = .698$, $n = 386$. And relative to the competent condition, subjects in the moral condition were more willing to help the target, $b = 2.56$ [2.23, 2.90], $t = 15.07$, $B = .60$, $p < .001$, and to punish on her behalf, $b = .48$ [.11, .86], $t = 2.52$, $B = .12$, $p = .012$, $n = 409$.

Thus, we find some evidence that describing victims as *morally* virtuous may be especially effective at motivating justice-restorative action. However, as we also mention in the main text, this evidence is merely suggestive because our morality manipulation (unexpectedly) turned out to be stronger than our competence manipulation. Specifically, the effect of the morality manipulation on perceived morality (as reported in the main text, $b = 3.55$ [3.30, 3.80], $t = 27.80$, $B = .81$, $p < .001$) was larger than the effect of the competence manipulation on perceived competence (as reported above, $b = 1.87$ [1.58, 2.16], $t = 12.79$, $B = .55$, $p < .001$).

We do note that the discrepancy between our manipulations appears to be relatively larger when considering our dependent variables (i.e., willingness to help and punish) than these traits (i.e., perceived morality and competence). But this comparison is nonetheless difficult to interpret, because the relationships between these traits and our dependent variables may not be linear.

2.8. Experiment 12

In the main text, we presented two sets of results from Experiment 12. First, we used Experiment 12 to support the claim that the Virtuous Victim effect is not merely a simple

contrast effect. To this end, we reported that subjects in the standard victim condition rated the target as having better moral character than subjects in the other victim condition. Second, we used Experiment 12 to support the claim that the Virtuous Victim effect does not extend to behavior predictions. To this end, we reported that, relative to subjects in the neutral condition, subjects in the standard victim condition did not predict that victims would return more money in a hypothetical economic Trust Game.

Here, we report more complete Experiment 12 results. In Fig. S4, we plot values, by condition, of each of the four DVs that Experiment 12 measured. Specifically, in Panel A, we plot our Trust Game DVs: the amount sent to target (as a proportion of starting endowment) and the predicted amount returned by target (as a proportion of amount sent). Then, in Panel B, we plot ratings of target morality and trustworthiness. In Table S6, we report results from linear regressions comparing the victim vs. neutral conditions, and victim vs. other victim conditions, on each of these four DVs.

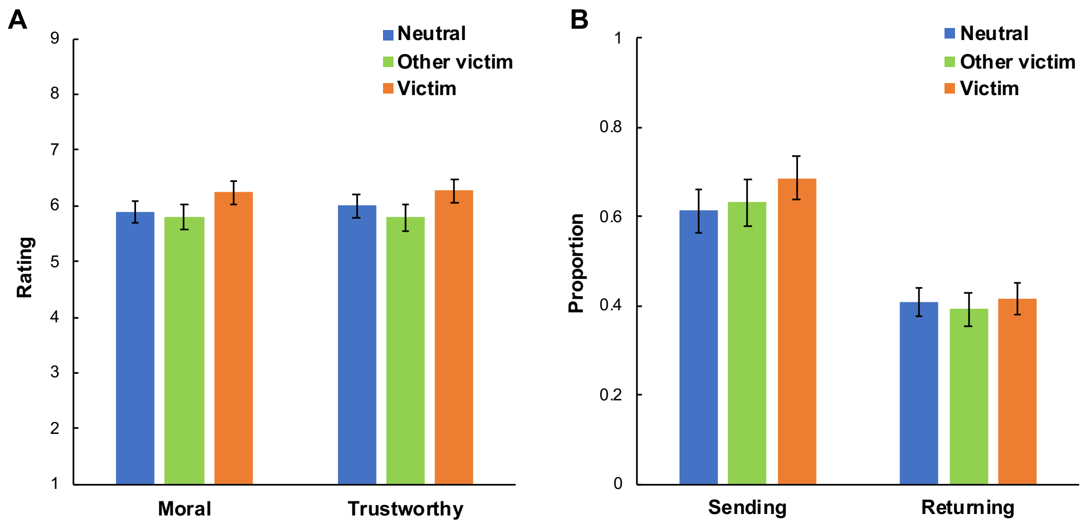

Fig. S4. Dependent variables across conditions in Experiment 12.

Variable	Victim vs. Neutral (n = 400)	Victim vs. Other victim (n = 402)
Amount sent to target in Trust Game	Neutral = 0.61, Victim = 0.69, **b = 0.07, t = 2.14, p = .033**	Other victim = 0.63, Victim = 0.69, **b = 0.06, t = 1.56, p = .119**
Predicted amount returned by target in Trust Game	Neutral = 0.41, Victim = 0.42, **b = 0.01, t = 0.29, p = .775**	Other victim = 0.39, Victim = 0.42, **b = 0.02, t = 0.90, p = .368**
Rating of target morality	Neutral = 5.88, Victim = 6.24, **b = 0.35, t = 2.43, p = .015**	Other victim = 5.81, Victim = 6.24, **b = 0.43, t = 2.82, p = .005**
Rating of target trustworthiness	Neutral = 6.00, Victim = 6.26, **b = 0.26, t = 1.73, p = .085**	Other victim = 5.79, Victim = 6.26, **b = 0.48, t = 2.96, p = .003**

Table S6. Effects of the victim condition on all dependent variables in Experiment 12.

Together, these results support the two key claims mentioned above. First, we find that people see victims as morally virtuous, and are willing to send them more money in a

hypothetical Trust Game—despite not predicting that they will return more money. These results show that the Virtuous Victim effect extends to moral character (and that people are motivated to share money with victims), but that people do not expect victims to behave more morally.

Second, we find that the Virtuous Victim effect does not merely reflect a simple contrast effect with the perpetrator. The effects of victim status on morality, trustworthiness, and Trust Game sending do not extend equally to the other victim condition (in which subjects read about a perpetrator mistreating a character other than the target).

2.9. Analyses of behavior predictions

In the main text, we reported that across all of our experiments measuring behavior predictions, we found no significant positive effects of our victim manipulation on moral behavior, or significant negative effects on immoral behavior. Here, we support this claim by reporting more extensive analyses of behavior predictions across our experiments.

2.9.1. Discussion of upsetness hypothesis

First, however, we provide further discussion of one aim of these experiments, briefly mentioned in the main text. Specifically, one aim of these experiments was to investigate the hypothesis that the Virtuous Victim effect does not extend to behavior predictions because people expect victims to be upset following their victimization, and believe that upset individuals are inclined to behave thus less morally.

According to this "upsetness" hypothesis, the observed null effects of victim status on behavior predictions actually reflect two countervailing effects. The upsetness hypothesis proposes that, on the one hand, people expect victims to behave *more* morally because people see victims as having elevated moral character. Yet on the other hand, people expect victims to behave *less* morally because they are upset, resulting in no net effect of victim status on predicted moral behavior. Bolstering the credibility of this hypothesis, we note that the victim conditions of almost all of our vignettes ended by stating the target was very upset.

We sought to address this upsetness hypothesis in two ways. First, in Experiment 14 we modified our vignette text to omit the sentence stating that the victim was upset. We anticipated that this omission would reduce the salience of the victim's upsetness (although subjects could of course still reason that the victim was probably upset).

Second, and more substantively, in Experiment 15 we sought to systematically investigate the upsetness hypothesis. The upsetness hypothesis makes a testable prediction: insofar as there are moral behaviors that people do *not* think are less likely among upset actors (but *do* think are more likely among actors with superior moral character), there should be some contexts in which people *do* expect victims to behave more morally. Thus, in Experiment 15, we investigated the effect of our victim manipulation on predictions regarding four distinct moral behaviors. A pre-test revealed that subjects expected two of these behaviors *to* be less likely among upset actors, and two of them *not* to be less likely among upset actors (but all four to be more likely among actors with superior moral character). For the behaviors that pre-test subjects did *not* see as less likely among upset actors, the upsetness hypothesis predicts a positive effect of victim status on predicted moral behavior.

2.9.2. Discussion of Experiment 15 pre-test

Here, we provide a design overview of our Experiment 15 pre-test, which we used to identify the behaviors that we used in Experiment 15, and report the pre-test results.

In our Experiment 15 pre-test, we generated a set of 13 candidate moral behaviors. We then employed a two-by-two, between-subject design, in which we directly manipulated target morality (immoral vs. moral) and upsetness (not upset vs. upset), and then asked each subject to rate the likelihood that the target would engage in a random subset of the candidate moral behaviors (resulting in a slightly different *n* for each behavior). For more detail, including the full text description of each behavior, see the "full experimental details" section of this document.

In Table S7, we report two sets of analyses for each behavior included in our pre-test. First, we report the effect of target upsetness on the perceived likelihood of engaging in the behavior, within the "moral" condition. This comparison allows us to ask: if a target is a victim, and thus is seen as having relatively good moral character, will perceived upsetness make them seem less likely to engage in the relevant moral behavior?

Second, we report the effect of target morality on perceived likelihood of engaging in the behavior, within the "upset" condition. This comparison allows us to ask: if a target is a victim, and thus is seen as being upset, will perceived moral character make them seem more likely to engage in the relevant moral behavior?

Behavior	Effect of upset if moral	Effect of moral if upset
Select local and charitable coffee shop over Starbucks	Not upset = 6.03, Upset = 5.85,<br>**b = -0.18, t = -0.76, p = .447, n = 200**	Immoral = 3.12, Moral = 6.03,<br>**b = 2.50, t = 10.00, p < .001, n = 189**
**Consent for waitress to give leftovers to homeless**	Not upset = 7.33, Upset = 7.21,<br>**b = -0.12, t = -0.59, p = .555, n = 194**	Immoral = 4.20, Moral = 7.33,<br>**b = 2.71, t = 10.05, p < .001, n = 200**
Make a habit of donating old clothes to Goodwill	Not upset = 5.90, Upset = 5.64,<br>**b = -0.26, t = -1.07, p = .286, n = 207**	Immoral = 2.94, Moral = 5.90,<br>**b = 2.66, t = 10.54, p < .001, n = 204**
Make a habit of tipping 20% at reastaurants	Not upset = 5.81, Upset = 5.40,<br>**b = -0.41, t = -1.76, p = .079, n = 218**	Immoral = 2.59, Moral = 5.81,<br>**b = 2.52, t = 9.93, p < .001, n = 209**
**Make a habit of recycling**	Not upset = 5.70, Upset = 5.65,<br>**b = -0.05, t = -0.21, p = .831, n = 204**	Immoral = 2.93, Moral = 5.70,<br>**b = 2.51, t = 10.06, p < .001, n = 205**
Always thanks driver before exiting bus/Uber	Not upset = 6.51, Upset = 6.21,<br>**b = -0.30, t = -1.28, p = .201, n = 195**	Immoral = 3.47, Moral = 6.51,<br>**b = 3.06, t = 12.14, p < .001, n = 211**
Always polite and gracious to people working in service	Not upset = 6.63, Upset = 6.38,<br>**b = -0.26, t = -1.19, p = .235, n = 183**	Immoral = 3.27, Moral = 6.63,<br>**b = 3.21, t = 12.60, p < .001, n = 202**
Put efforting into planning surprise birthday party	Not upset = 5.50, Upset = 5.20,<br>**b = -0.30, t = -1.32, p = .189, n = 220**	Immoral = 3.88, Moral = 5.50,<br>**b = 1.61, t = 6.33, p < .001, n = 218**
Volunteer to help habitat for humanity	Not upset = 5.10, Upset = 4.98,<br>**b = -0.12, t = -0.48, p = .629, n = 190**	Immoral = 2.44, Moral = 5.10,<br>**b = 2.24, t = 8.98, p < .001, n = 188**
**Offer to loan money to a friend in need**	Not upset = 4.48, Upset = 3.99,<br>**b = -0.49, t = -1.89, p = .060, n = 206**	Immoral = 2.45, Moral = 4.48,<br>**b = 1.54, t = 6.21, p < .001, n = 202**
**Surprise mom with gift**	Not upset = 6.13, Upset = 5.00,<br>**b = -1.13, t = -4.23, p < .001, n = 188**	Immoral = 3.37, Moral = 6.13,<br>**b = 1.78, t = 6.52, p < .001, n = 191**
Stop patronizing racist sandwich shop	Not upset = 5.42, Upset = 5.05,<br>**b = -0.37, t = -1.12, p = .263, n = 195**	Immoral = 3.96, Moral = 5.42,<br>**b = 1.11, t = 3.28, p = .001, n = 205**

Table S7. Experiment 15 pre-test results.

Table S7 reveals that for all behaviors, we found a significant positive effect of morality (among upset targets) on the perceived likelihood of engaging in the behavior. In contrast, we only found a significant or marginally significant negative effect of upsetness (among moral targets) for two behaviors. The four behaviors we selected to use in Experiment 15 are bolded. The top two are the behaviors we selected that subjects did *not* expect to be less likely among upset targets: consenting to a waitress giving one's leftover food to a homeless person, and making a habit of recycling. The bottom two are the behaviors we selected that subjects *did*

expect to be less likely among moral targets: offering to loan money to a friend in need and surprising one's mother with a gift.

2.9.3. Full behavior prediction results

In Table S8, we report the full set of results from each experiment that measured behavior predictions. We include both our set of victim manipulation experiments that measured behavior predictions (Experiments 1, 7, 12, 14, and 15 and our direct morality manipulation experiment (Experiment 16)). For each experiment, we report the effect of our manipulation (for our victim manipulation experiments, we compare the neutral and victim conditions, and for our direct morality manipulation experiment, we compare the control and moral conditions) on the perceived likelihood of engaging in each moral and immoral behavior included in the experiment. We also plot these comparisons in Fig. S5.

	**Effects of victim manipulation on moral behavior**	**Effects of victim manipulation on immoral behavior**
**Experiment 1 (n = 403)**	**Behaved morally over the past year:** Neutral = 4.22, Victim = 4.02, b = -0.20, t = -0.88, p = .382	**Behaved immorally over the past year:** Neutral = 4.20, Victim = 4.33, b = 0.13, t = 0.60, p = .549
	**Will behave morally over the next year:** Neutral = 4.19, Victim = 3.84, b = -0.35, t = -1.62, p = .106	**Will behave immorally over the next year:** Neutral = 4.25, Victim = 4.36, b = 0.11, t = 0.51, p = .609
	**Volunteered to help others over the past year:** Neutral = 4.14, Victim = 4.02, b = -0.12, t = -0.53, p = .594	**Lied to others over the past year:** Neutral = 4.09, Victim = 4.15, b = 0.06, t = 0.27, p = .785
	**Will volunteer to help others over the next year:** Neutral = 4.24, Victim = 3.77, b = -0.47, t = -2.21, p = .028	**Will lie to others over the next year:** Neutral = 4.21, Victim = 4.16, b = -0.05, t = -0.22, p = .829
**Experiment 7 (n = 401)**	**Donate to charity:** Neutral = 6.52, Victim = 6.58, b = 0.06, t = 0.40, p = .691	**Flirt with others while married:** Neutral = 2.73, Victim = 2.74, b = 0.01, t = 0.05, p = .963
	**Help coworker:** Neutral = 5.97, Victim = 6.19, b = 0.22, t = 1.33, p = .184	**Intentionally forget to pay friend back:** Neutral = 2.59, Victim = 2.66, b = 0.07, t = 0.38, p = .705
	**Plan birthday surprise for spouse:** Neutral = 6.04, Victim = 6.24, b = 0.21, t = 1.23, p = .220	**Harshly scold coworker for honest mistake:** Neutral = 3.30, Victim = 3.32, b = 0.03, t = 0.13, p = .895
	**Buy dinner for friend:** Neutral = 6.07, Victim = 6.19, b = 0.13, t = 0.85, p = .397	**Spread mean gossip:** Neutral = 2.78, Victim = 2.74, b = -0.04, t = -0.22, p = .824
**Experiment 12 (n = 400)**	**Return money in economic Trust Game:** Neutral = 0.41, Victim = 0.42, b = 0.01, t = 0.29, p = .775	
**Experiment 14 (n = 403)**	**Donate to charity:** Neutral = 4.69, Victim = 4.93, b = 0.24, t = 1.33, p = .185	**Flirt with others while in relationship:** Neutral = 2.82, Victim = 2.73, b = -0.08, t = -0.49, p = .627
	**Help friend move:** Neutral = 5.84, Victim = 5.70, b = -0.14, t = -0.84, p = .402	**Intentionally forget to pay friend back:** Neutral = 2.46, Victim = 2.61, b = 0.16, t = 0.88, p = .380
	**Volunteer to tutor:** Neutral = 4.52, Victim = 4.80, b = 0.28, t = 1.56, p = .119	**Exclude or betray friend to fit in:** Neutral = 2.60, Victim = 2.64, b = 0.04, t = 0.24, p = .813
	**Give leftovers to homeless:** Neutral = 4.92, Victim = 5.08, b = 0.17, t = 0.96, p = .339	**Spread mean gossip:** Neutral = 2.62, Victim = 2.80, b = 0.18, t = 0.95, p = .344
**Experiment 15 (n = 401)**	**Consent for waitress to give leftovers to homeless:** Neutral = 6.80, Victim = 6.28, b = -0.52, t = -3.12, p = .002	
	**Make a habit of recycling:** Neutral = 4.95, Victim = 4.93, b = -0.02, t = -0.16, p = .877	
	**Offer to loan money to a friend:** Neutral = 3.69, Victim = 3.78, b = 0.08, t = 0.43, p = .668	
	**Surprise mom with gift:** Neutral = 5.34, Victim = 5.31, b = -0.03, t = -0.16, p = .870	
**Experiment 16: Direct morality manipulation (n = 201)**	**Donate to charity:** Control = 4.97, Moral = 5.58, b = 0.61, t = 2.65, p = .009	**Flirt with others while in relationship:** Control = 3.08, Moral = 2.66, b = -0.41, t = -1.67, p = .096
	**Help friend move:** Control = 5.98, Moral = 6.49, b = 0.51, t = 2.19, p = .030	**Intentionally forget to pay friend back:** Control = 2.78, Moral = 2.45, b = -0.33, t = -1.37, p = .173
	**Volunteer to tutor:** Control = 4.87, Moral = 5.53, b = 0.66, t = 2.77, p = .006	**Exclude or betray friend to fit in:** Control = 2.66, Moral = 2.52, b = -0.14, t = -0.58, p = .562
	**Give leftovers to homeless:** Control = 5.31, Moral = 5.71, b = 0.40, t = 1.74, p = .084	**Spread mean gossip:** Control = 2.78, Moral = 2.54, b = -0.24, t = -0.97, p = .331

Table S8. Effects of our victim manipulations (in Experiments 1,7,12,14,15) and our direct morality manipulation (in Experiment 16) on behavior predictions.

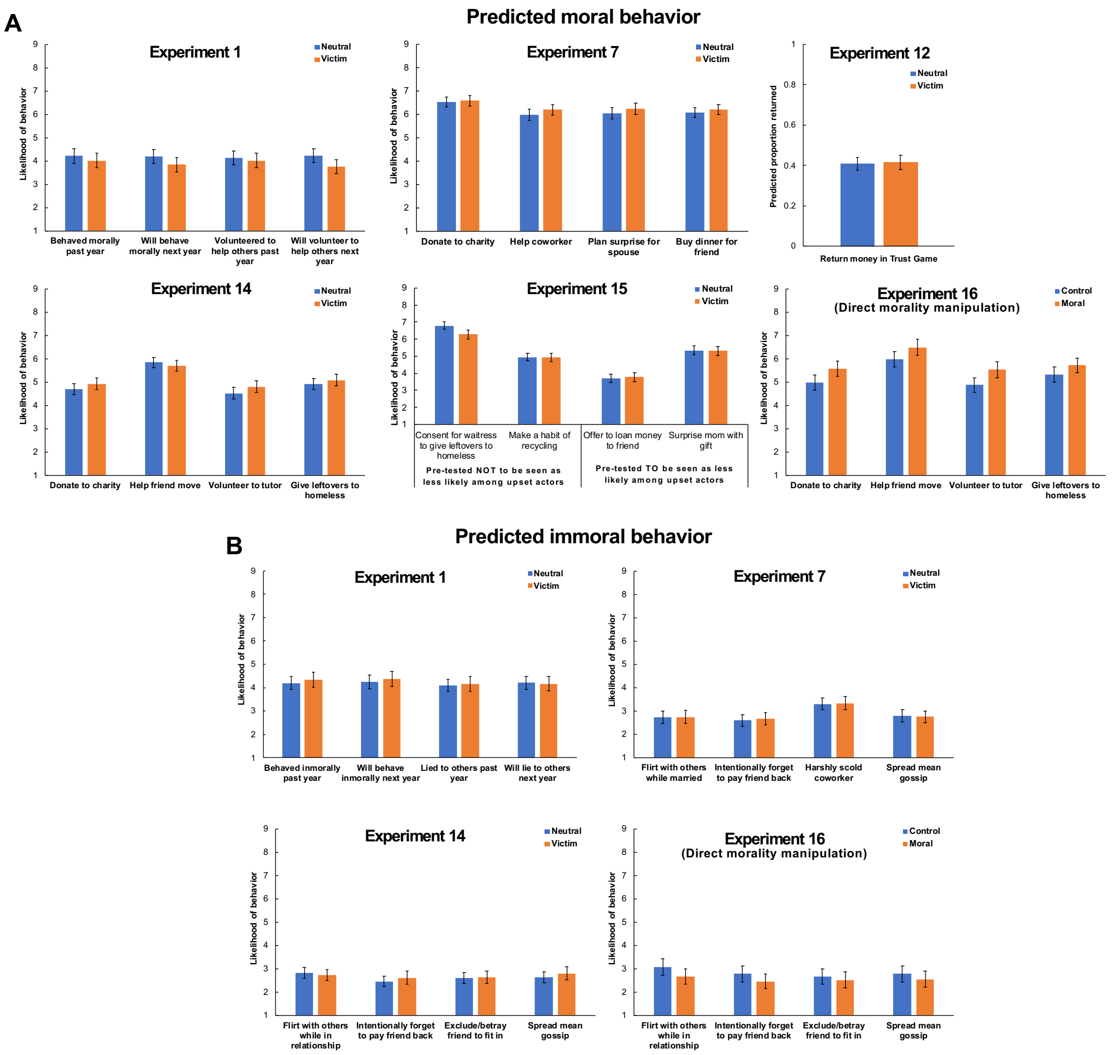

Fig. S5. Effects of our victim manipulations (in Experiments 1,7,12,14,15) and our direct morality manipulation (in Experiment 16) on behavior predictions.

These results reveal no significant positive effects of victim status on any moral behaviors, and no significant negative effects of victim status on any immoral behaviors. This pattern very robustly supports our claim that while subjects see victims as having elevated moral character, they do *not* see victims as more likely to behave morally or less likely to behave immorally. In contrast, in the context of our direct morality manipulation, which used the same set of behaviors as Experiment 14, we find a significant positive effect of the manipulation on three of the four moral behaviors, and a marginally significant positive effect on the fourth. We also find a marginally significant negative effect of the manipulation on one of the four immoral behaviors. These results suggest that the null effects of our victim manipulation on predicted moral behavior do not simply reflect that is too difficult to manipulate predicted behavior.

Our results also negate the hypothesis that the null effects of victim status on behavior predictions reflect that people expect victims to be upset following their victimization, and believe that upset individuals behave less morally. In Experiment 14, we did not directly inform subjects in the victim condition that the target was upset, and yet subjects still did not expect victims to behave more morally than non-victims. And in Experiment 15, we measured

predictions regarding two behaviors that, according to our pre-test, subjects did *not* see as less likely among upset actors, but did see as more likely among moral actors (e.g., habitually recycling). Even for these behaviors, we found no positive effects of our victim manipulation. Together, these data suggest that the expectation that victims will be upset does *not* explain the observed discrepancy between the effect of victim status on perceived moral character and predicted moral behavior.

3. Discussion of pre-registered analysis plans

As described in the main text, we individually pre-registered 15 of our 17 experiments. However, because we conducted a large number of experiments employing similar designs, we chose not to individually report the results for each experiment. Instead, we structure our main text around a series of claims, and when many experiments are relevant to a particular claim, we report aggregate analyses to support that claim. This approach both facilitates brevity and allows us to provide statistical estimates that are less noisy and more precise. However, it also lead us to deviate from our pre-registered analysis plans.

In the interest of transparency, we have made all of our pre-registered analysis plans available (see Table S1 for links). Overall, the analyses we present in the main text (and the above elaborations of these analyses) adhere very closely to the primary analyses that we pre-registered, despite some deviations or additional tests that were not pre-registered. Furthermore, in cases where we do not report the exact pre-registered analyses, we generally do still address the set of questions the pre-registered analyses sought to investigate (e.g., via analyses aggregating relevant data across experiments). However, we note that that the labeling of conditions and variables in our paper deviates somewhat from the labeling used in our pre-registrations (reflecting that our conceptual framework for this work, and thus the language we found most clear, evolved throughout the project).

Furthermore, for three of our experiments, there are pre-registered primary analyses investigating substantive questions that we do *not* address in our main text analyses or the above elaborations of them. In the "supplemental analyses" section of this document, below, we go through each of these "primary" deviations and explain the conceptual basis for the deviation, and report relevant analyses as appropriate.

We also note that our pre-registered analysis plans also involved several secondary and/or exploratory analyses investigating questions that we chose not to address in our main text analyses (or in the above elaborations of these analyses) For reasons of brevity, we do not discuss each of these "secondary" deviations in this document. However, in the "supplemental analyses" section of this document, below, we report aggregate analyses of two secondary dependent variables that we collected across multiple experiments (sympathy and exploitability). We also note that our data, including all secondary variables, are publicly available at https://osf.io/ckxb3/.

Finally, we note that in general, despite pre-registering several analyses involving mediation (mostly as secondary and/or exploratory analyses, but also as primary analyses in two cases, discussed in more detail below), we chose not to report the results of any mediation analyses in our main text or SM. We made this choice both for reasons of brevity (given the large number of experiments conducted and potential mediating variables collected), and also because mediation analyses are correlational, and thus do not allow us to make strong causal claims about mechanism (or discriminate between different causal models that predict the same

correlation structure). For these reasons, we ultimately chose to support claims about mechanism only by considering the causal effects of our experimental manipulations.

4. Supplemental analyses

4.1. Analyses pertaining to primary deviations from pre-registrations

4.1.1. Experiment 4

As discussed in the main text, the Justice Restoration Hypothesis predicts that the Virtuous Victim effect should only occur when people perceive incentives for justice-restorative action. We provide direct evidence for this prediction in Experiment 11, in which we crossed our victim manipulation with a manipulation of disincentives for justice-restorative action. In Experiment 4, we also tried to provide evidence for this prediction—not by manipulating incentives for justice-restorative action, but instead by measuring a potential index of such incentives.

Specifically, in Experiment 4 we measured a set of ideology variables, and then employed two vignettes describing transgressions for which we anticipated that ideology would shape incentives for justice-restorative action: the verbal attack of a gun owner and sexual aggression. We predicted that in the context of our verbal attack vignette, individuals with conservative ideology (in general, and in particular with respect to the issue of gun control) would be more likely to show the Virtuous Victim effect, because they would perceive stronger incentives to punish the perpetrator and/or help the victim. And in the context of our sexual aggression vignette, we predicted that individuals with liberal ideology (in general, in particular with respect to the issue of sexual harassment) would be more likely to show the Virtuous Victim effect, because they would perceive stronger incentives for justice-restorative action.

However, we ended up finding that for both our verbal attack and sexual aggression vignettes, ideology predicted evaluations of target moral character even in the *neutral* condition (in which no transgression occurred). This finding suggests that ideology does not provide a clean index of incentives for justice-restorative action (to treat as a potential moderator of the Virtuous Victim effect), because ideology is associated with our dependent variable even when no transgression has occurred (and thus justice-restorative action is not relevant). Thus, in Experiment 11, we sought to provide a more informative test of the Justice Restoration Hypothesis by using a manipulation to introduce disincentives for justice-restorative action (rather than measuring an index of incentives for justice-restorative action).

For this reason, in the main text, we simply report the main effect of our victim manipulation on target moral character for each Experiment 4 vignette, and do not report analyses of moderation by ideology. Here, however, we do report these analyses.

As described in detail in the "full experimental methods" section of this document, we began Experiment 4 by measuring a set of ideology variables. In particular, we measured ideology (via three-item scales) with respect two specific moral issues that were related to vignettes included in Experiment 4: gun control (related to our verbal attack vignette, in which a gun owner is verbally attacked for his gun ownership) and sexual harassment (related to our sexual aggression vignette). We also measured ideology with respect to two "distractor" moral issues (racism by police officers and climate change), as well as general political ideology (via three questions about political party affiliation, social conservativism, and fiscal conservativism).

We then used these measures to create three composite ideology variables, which coded for liberal ideology with respect to gun control, sexual harassment, and general political ideology, by averaging across the three items relevant to each construct. In Fig. S6, we plot the effect of our victim manipulation on target moral character for our verbal assault (Panel A) and sexual aggression (Panel B) vignettes, as a function of the relevant specific ideology composite variable (i.e., gun control ideology for our verbal assault vignette and sexual assault ideology for our sexual aggression vignette). To do so, we bin responses to our composite ideology variables (which range in value from 1-7) into six categories (corresponding to each of the six intervals between the integers 1-7).)

In Table S9, we report the results from a series of regressions predicting target moral character (i) as a function of liberal ideology in the neutral condition, (ii) as a function of liberal ideology in the victim condition, and (iii) as a function of liberal ideology, our victim manipulation (0 = neutral, 1 = victim), and their interaction (across both conditions). For our verbal attack and sexual aggression vignettes, we report these results both for the relevant specific ideology composite (gun control for our verbal attack vignette, and sexual harassment for our sexual aggression vignette) and for our general ideology composite. For our iPad theft vignette, we report these results for our general ideology composite only.

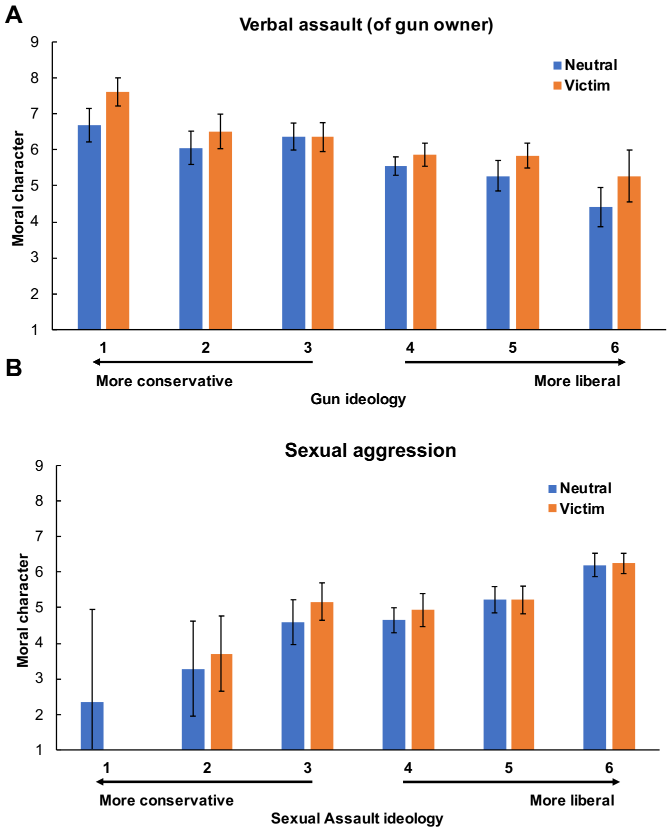

Fig. S6. Effects of our victim manipulation on target moral character as a function of specific ideology, for our verbal assault and sexual aggression vignettes in Experiment 4.

	Verbal attack (on gun owner)	Sexual aggression	iPad theft
**Specific ideology**			
Neutral (n = 255)	b = -0.43, t = -7.62, p < .001	b = 0.64, t = 8.45, p < .001	
Victim (n = 255)	b = -0.42, t = -7.51, p < .001	b = 0.48, t = 6.19, p < .001	
Interaction (n = 510)	b = 0.01, t = 0.12, p = .903	b = -0.16, t = -1.46, p = .145	
**General ideology**			
Neutral (n = 255)	b = -0.20, t = -3.62, p < .001	b = 0.39, t = 6.33, p < .001	b = 0.01, t = 0.25, p = .801
Victim (n = 255)	b = -0.34, t = -5.77, p < .001	b = 0.29, t = 4.38, p < .001	b = -0.06, t = -1.01, p = .316
Interaction (n = 510)	b = -0.14, t = -1.72, p = .087	b = -0.10, t = -1.09, p = .277	b = -0.08, t = -0.93, p = .354

Table S9. Associations between specific and general liberal ideology and target moral character, as a function of our victim manipulation, across vignettes in Experiment 4.

As discussed above, we originally predicted that the Virtuous Victim effect would be stronger among conservatives for the verbal attack vignette, and stronger among liberals for the sexual aggression vignette. As predicted by this proposal, in the victim condition, conservative ideology is consistently negatively associated with target character ratings for the verbal attack vignette, and consistently positively associated with target character ratings for the sexual aggression vignette. Yet these ideology associations also held in the neutral condition (in which no transgression occurred). And contrary to our original prediction, we found no significant interaction between ideology and victim status for either vignette or ideology measure.

We note that we did find a marginally significant interaction in the predicted direction for the verbal attack vignette and our general ideology variable, reflecting that conservatives showed a somewhat larger Virtuous Victim effect for this vignette. However, looking to this general ideology variable, we see that conservatives showed a directionally larger Virtuous Victim effect for all three of our vignettes, suggesting that this trend should not be interpreted as providing support for our prediction.

These patterns reveal, as noted above, that in the context of our verbal attack and sexual aggression vignettes, ideology is associated with subjects' evaluations of target moral character even in the absence of a transgression. And as argued above, they therefore complicate the interpretation of ideology as an index of incentives to for justice-restorative action (and potential moderator of the Virtuous Victim effect). Thus, we argue that Experiment 4 does not provide a clearly interpretable test of the Justice Restoration Hypothesis.

4.1.2. Experiment 11a

Recall that in Experiment 11a, we crossed a manipulation of disincentives for justice-restorative action with our standard victim manipulation in the context of our idea theft vignette, and found that the Virtuous Victim effect was eliminated in the "disincentives" condition. In addition to collecting our primary DVs (moral evaluations of the target), Experiment 11a also measured a set of secondary variables.

Specifically, before measuring our primary DVs, we measured, from subjects in all conditions, moral evaluations of the "other person" (who was a neutral character in the neutral conditions, and the perpetrator of idea theft in the victim conditions). And after collecting our primary DV, we measured, from subjects in all conditions, perceived reputation-based incentives to punish the other person, willingness to punish the other person, perceived reputation-based incentives to help the target, willingness to help the target, and sympathy for the target. (We note that punishment of the other person was described specifically as "giving [the other person] the cold shoulder.") Finally, for subjects in the victim conditions only, we also measured anger

towards and blame of the other person. Sympathy, anger, and blame were each measured via three-item scales; we report analyses of composite variables computed by averaging these three items.

We show the effects of our manipulations on each of these secondary variables (as well as our primary dependent variables, which measure moral evaluations of the target) in Fig. S7 (which plots each variable by condition) and Table S10 (which reports the effect of an "disincentives" dummy separately for the neutral and victim conditions, as well as the interaction between a "disincentives" dummy and a "victim" dummy).

Our Experiment 11a pre-registration also included several specific analyses of our secondary variables. However, our conceptual framework for our work has shifted since we wrote our pre-registration. Thus, in the following sections we interpret our secondary variable results in light of our current conceptual understanding, and then close by describing our set of pre-registered analyses.

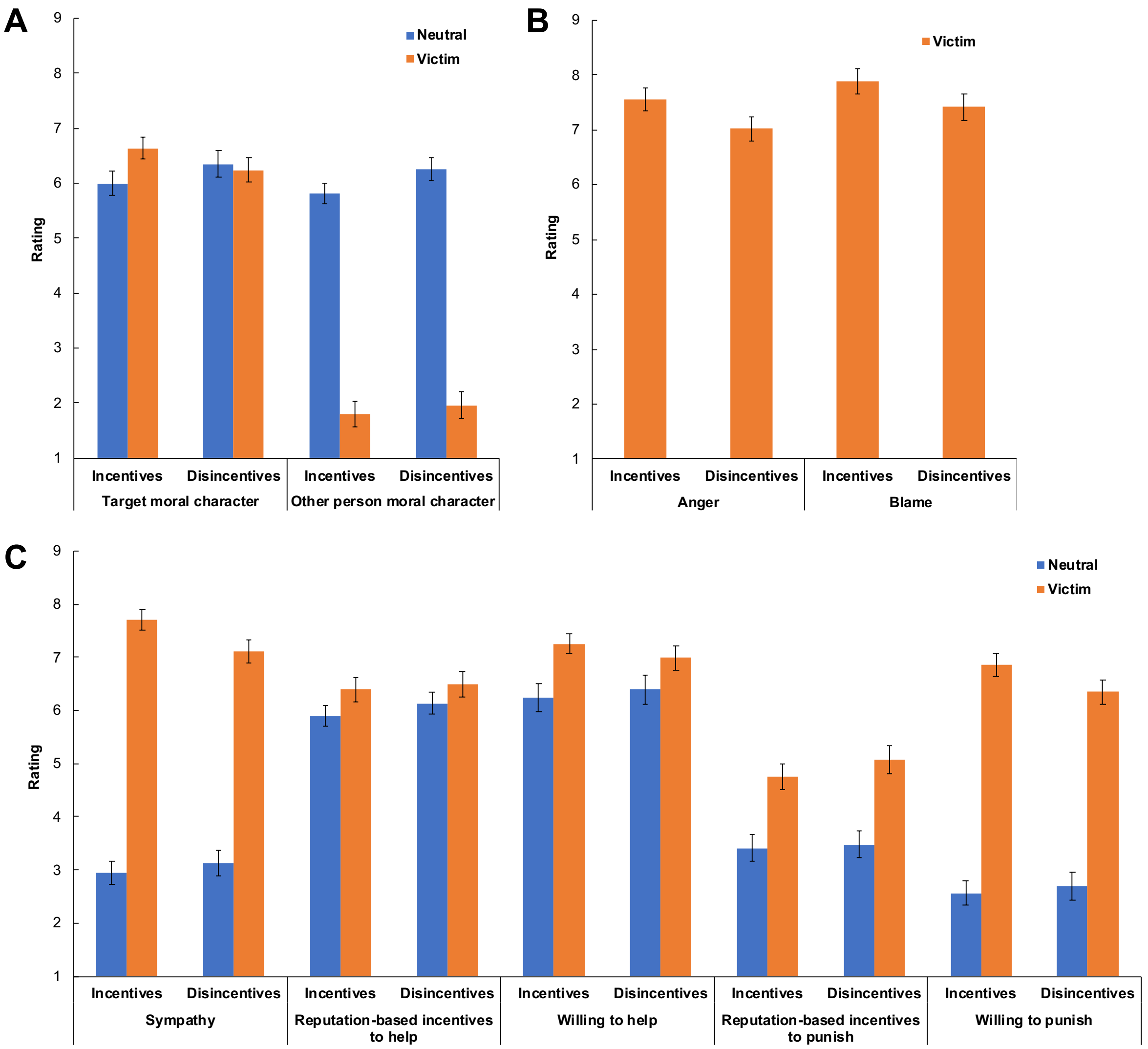

Fig. S7. Dependent variables across conditions in Experiment 11a.

Variable	Effect of Disncentives in Neutral (n = 401)	Effect of Disincentives in Victim (n = 400)	Disincentives X Victim interaction
Anger towards other person		Incentives = 7.56, Disincentives = 7.02, b = -0.54, t = -3.38, p = .001	
Blame of other person		Incentives = 7.88, Disincentives = 7.41, b = -0.47, t = -3.28, p = .001	
Morality of target	Incentives = 6.00, Disincentives = 6.39, b = 0.39, t = 2.31, p = .021	Incentives = 6.58, Disincentives = 6.23, b = -0.35, t = -2.21, p = .027	**b = -0.74, t = -3.20, p = .001**
Trustworthiness of target	Incentives = 6.00, Disincentives = 6.30, b = 0.30, t = 1.76, p = .080	Incentives = 6.69, Disincentives = 6.26, b = -0.43, t = -2.73, p = .007	**b = -0.73, t = -3.15, p = .002**
Morality of other person	Incentives = 5.83, Disincentives = 6.23, b = 0.40, t = 2.69, p = .007	Incentives = 1.85, Disincentives = 2.01, b = 0.16, t = 0.89, p = .372	**b = -0.24, t = -1.06, p = .290**
Trustworthiness of other person	Incentives = 5.80, Disincentives = 6.27, b = 0.47, t = 3.04, p = .003	Incentives = 1.74, Disincentives = 1.91, b = 0.18, t = 1.01, p = .315	**b = -0.29, t = -1.25, p = .210**
Sympathy towards target	Incentives = 2.94, Disincentives = 3.13, b = 0.19, t = 0.96, p = .338	Incentives = 7.70, Disincentives = 7.10, b = -0.60, t = -4.04, p < .001	**b = -0.79, t = -3.20, p = .001**
Reputaton-based incentives to help target	Incentives = 5.90, Disincentives = 6.13, b = 0.23, t = 1.32, p = .189	Incentives = 6.40, Disincentives = 6.49, b = 0.10, t = 0.54, p = .591	**b = -0.14, t = -0.54, p = .589**
Willingness to help target	Incentives = 6.24, Disincentives = 6.39, b = 0.15, t = 0.86, p = .391	Incentives = 7.25, Disincentives = 6.99, b = -0.26, t = -1.57, p = .116	**b = -0.41, t = -1.70, p = .090**
Reputaton-based incentives to punish other person	Incentives = 3.42, Disincentives = 3.48, b = 0.06, t = 0.33, p = .744	Incentives = 4.75, Disincentives = 5.08, b = 0.34, t = 1.78, p = .075	**b = 0.27, t = 1.02, p = .310**
Willingness to punish other person	Incentives = 2.57, Disincentives = 2.70, b = 0.13, t = 0.60, p = .551	Incentives = 6.86, Disincentives = 6.34, b = -0.51, t = -2.47, p = .014	**b = -0.64, t = -2.16, p = .031**

Table S10. Condition effects on all dependent variables in Experiment 11a.

Evaluating the success of our disincentives manipulation. As discussed in the main text, we conducted Experiment 11b to serve as a post-hoc manipulation check for Experiment 11a. In particular, we used Experiment 11b to evaluate the success of our disincentives manipulation in two key ways. First, we sought to confirm that people actually perceive weaker incentives for justice-restorative action in the scenario described by our "disincentives" (vs. "incentives") condition. Second, we sought to confirm that taking on the perspective described by our "disincentives" condition does not prevent people from seeing idea theft as a genuine transgression that creates genuine victims. And indeed, Experiment 11b provides clear evidence for both of these claims, suggesting that our disincentives manipulation was successful.

In addition to this evidence, however, the secondary variables collected within Experiment 11a can also shed some light on the success of our disincentives manipulation. Here, we interpret the secondary variables in this light.

To begin, the secondary variables provide further evidence that subjects in the victim version of the "disincentives" condition of Experiment 11a really did see idea theft as a genuine transgression. These subjects rated the "other person" (who, in the victim conditions, was the idea theft perpetrator) as very immoral and untrustworthy. In fact, their moral evaluations of the perpetrator did not differ significantly from their counterparts in the "incentives" condition. Furthermore, in absolute terms, they also reported substantial anger towards, blame of, and willingness to punish the perpetrator, as well as sympathy towards the victim.

(We also briefly note that within the *neutral* conditions of our experiment, where no transgression occurred, we see an unexpected positive effect of the "disincentives" condition on moral evaluations of the other person, and also on moral evaluations of the target. One plausible explanation for these unexpected results is that in the "disincentives" condition, subjects were asked to imagine believing that idea theft is less common; for this reason, they may have had higher baseline moral evaluations of people at the advertising agency. Regardless, we see these

results as unrelated to the question of how our disincentives manipulation influenced moral evaluations of the perpetrator in the *victim* conditions of our experiment, where theft occurred.)

Thus, our secondary DVs bolster the conclusion that subjects in the “disincentives” condition of Experiment 11a *did* see idea theft as a genuine transgression. And they consequentially bolster our conclusion that the “disincentives” condition really did eliminate the Virtuous Victim effect by reducing perceived incentives for justice-restorative action—and not simply by preventing subjects from seeing idea theft as morally wrong (and thus seeing the target as a true victim).

Notably, however, the “disincentives” condition did *not* reduce reported reputation-based incentives to punish or reputation-based incentives to help. Thus, our secondary DVs from Experiment 11a do *not* provide further evidence that our “disincentives” condition really did reduce perceived incentives for justice-restorative action. Yet we see our secondary DVs as providing a less informative measure of incentives than Experiment 11b, for three reasons.

First, our secondary DVs in Experiment 11a asked specifically about *reputation-based* incentives to help and punish, whereas Experiment 11b asked about incentives more generally. And it is plausible that the “disincentives” condition reduced perceived *non-reputational* incentives for justice-restorative action. In the “incentives” condition, subjects imagined disapproving of the brainstorming system that made idea theft possible. Thus, they may have perceived (non-reputational) incentives to draw attention to idea theft via justice-restorative action, in order to achieve their goal of dismantling the brainstorming system. In contrast, subjects in the “disincentives” condition imagined *valuing* the brainstorming system, and correspondingly may *not* have perceived such incentives.

Second, subjects in Experiment 11a were asked to imagine a situation, and then at the end of the study were asked to report the incentives that they would face in that situation. In contrast, subjects in Experiment 11b were asked to evaluate, from an objective third-party perspective, the incentives that subjects in Experiment 11a imagined facing. And we see this objective third-party perspective as providing a “purer” measure of incentives.

Finally, when designing our incentives measures in Experiment 11b, we provided subjects with better definitions of helping and punishment. In particular, our secondary DVs in Experiment 11a simply described helping with the phrase “*If you helped Sarah out*”, and described punishment with the phrase “*If you gave Gabrielle the cold shoulder*”. In contrast, Experiment 11b described helping by saying “*Imagine that James is considering helping Sarah. For example, James is considering helping Sarah to brainstorm new ideas, being especially warm and friendly towards Sarah when he sees her at work, making sure to praise Sarah for her work in front of others, or taking other similar action*” and described punishment by saying “*Imagine that James is considering "punishing" Gabrielle for presenting Sarah's idea as her own. For example, James is considering telling other people what Gabrielle did to Sarah and expressing his disapproval of Gabrielle's behavior, giving Gabrielle the cold shoulder when he sees her at work, reporting Gabrielle's behavior formally, or taking some other similar action*”.

For these reasons, we see Experiment 11b as providing better insight into whether our disincentives manipulation actually influenced perceived incentives for justice-restorative action. And thus we believe that there is good reason to believe that our disincentives manipulation was successful in this goal, despite the fact that the “disincentives” condition did not reduce reported reputation-based incentives to punish or help in Experiment 11a.

Analyses of motivation for justice-restorative action. In the main text, we show that the "disincentives" condition of Experiment 11a caused the Virtuous Victim effect to disappear. Our secondary DVs also show that it reduced motivation for justice-restorative action.

To support this claim, we first considered our set of secondary DVs that can be seen as measures of motivation for punishment: anger towards, blame of, and willingness to punish the other person. We only measured anger and blame in victim conditions (in which a transgression occurred); within these conditions, we find that subjects in the "disincentives" condition reported less anger towards and blame of the other person (i.e., the idea theft perpetrator). We also measured willingness to punish in all conditions, and find that (i) within the victim conditions, subjects in the "disincentives" condition reported less willingness to punish, (ii) this effect is specific to the victim conditions (in which a transgression occurred), as reflected by a significant interaction between incentives and victim status.

Thus, subjects in the "disincentives" version of the victim conditions reported less motivation to punish the perpetrator, as measured by blame, anger, and willingness to punish. We also found some evidence that they felt less motivation to help the victim, as measured by sympathy for and willingness to help the target. Both of these variables were measured in all conditions, and show the same pattern of results as our measure of willingness to punish—although the results are highly significant for sympathy, but marginal for willingness to help.

Together, then, our set of Experiment 11a results suggest that subjects in the "disincentives" condition saw idea theft as a genuine transgression. Yet they did *not* morally elevate the victim, and they reported less motivation to engage in justice-restorative action. These results are consistent with the Justice Restoration Hypothesis, and its proposal that introducing disincentives for justice-restorative action should serve to eliminate the Virtuous Victim effect—and consequently prevent people from feel especially motivated to engage in justice-restorative action.

Pre-registered analysis plan. Finally, we describe the specific analyses of our secondary variables that we pre-registered.

In our Experiment 11a pre-registration, we planned to analyze moral evaluations of the other person, blame, and anger as manipulation checks. We predicted that within the victim conditions, we might observe positive effects of the "incentives" (vs. "disincentives") condition on blame and anger, and/or negative effects on moral evaluations of the other person. Furthermore, we predicted that if we did find negative effects on other person character, that they would not hold or be weaker in the "neutral" condition (leading to a negative interaction between the "incentives" and "victim" conditions on other person character). Above, we discuss the effectiveness of our manipulation; however, with our current conceptual framework, we see a somewhat different set of variables as the most relevant to this discussion.

We also planned to treat the rest of our secondary variables (i.e., perceived reputational incentives to punish, willingness to punish, perceived reputational incentives to help, willingness to help, and sympathy) as additional dependent variables, and planned for each variable to investigate (i) the effects of victim status, our "incentives" condition, and their interaction, as well as (ii) the simple effect of victim status in the "incentives" condition. For all variables, we predicted that there might be positive interactions between our "incentives" and "victim" conditions, and that the simple effect of victim status in the "incentives" condition would be positive. We also planned, as secondary analyses, to conduct exploratory moderated mediation analyses treating these variables, as well as moral evaluations of the other person, as candidate mediators of the interaction between incentives and victim status on moral character.

Above, we discuss the effects of our manipulation on all of these variables. We do not conduct the planned moderation mediation analyses because, as described above, we generally do not include mediation analyses in our paper (both for reasons of brevity and in order to focus on causal claims that we can support more confidently). However, here we briefly discuss another point, which is specific to these particular mediation analyses: they were designed to test a causal model that differs somewhat from the causal model we focus on in the main text.

When pre-registering these mediation analyses, we imagined a causal model in which, within our victim conditions (i.e., when a transgression occurs), (i) our "incentives" condition increases motivation for justice-restorative action and (ii) this in turn causes people to see victims as having elevated moral character. According to this causal model, it is appropriate to treat motivation for justice-restorative action as a mediator of the interaction between our "incentives" condition and victim status on moral character. Yet our as theorizing developed, we shifted our focus to a different causal model. Specifically, our paper ultimately proposes a causal model in which (i) incentives for justice-restorative action cause people to elevate morality of victims and (ii) this in turn increases motivation for justice-restorative action. According to *this* causal model, it is not appropriate to treat motivation for justice-restorative action as a mediator of the interaction between our "incentives" condition and victim status on moral character.

In fact, our set of results is actually consistent with either model. In other words, while we propose in our paper that the moral elevation of victims serves to adaptively boost motivation for justice-restorative action, our data are also compatible with the proposal that adaptive motivation for justice-restorative action causes the moral elevation of victims. Critically, both models support our overarching theoretical proposal that incentives for justice-restorative action give rise to the Virtuous Victim effect and motivation for justice-restorative action. Future research should nonetheless attempt to discriminate empirically between the two models; however, our Experiment 11a data do not allow us to do so (or to discriminate them from other models that predict the same correlation structure between variables).

4.1.3. Experiment 13

As discussed in the main text, Experiment 13 reveals that subjects report stronger (reputation-based) incentives to help victims of immorality—both as compared to neutral targets and victims of accidental misfortune. In addition to measuring incentives to help the target (as well as target morality and trustworthiness), Experiment 13 measured three other variables that we do not analyze in the main text.

First, we measured *willingness* to help the target, in order to test the hypothesis that people are particularly motivated to help victims of immorality (in line with the perception that doing so has particularly large reputational benefits). Second, we measured (reputation-based) incentives, and willingness, to *affiliate with* the target (described to subjects as "hanging out" with the target). We collected these affiliation measures to investigate the possibility that people generally perceive elevated incentives to (and are thus more willing to) interact with victims of immorality, in a way that is not specific to helping. However, in the main text we focus specifically on *incentives* to *help* the target (and not willingness to help or either affiliation variable) because we see incentives to help as most relevant to our theoretical claims.

We measured incentives and willingness to help and affiliate with the target all on the same page (and randomized the order of these four variables). In our pre-registration, we planned to use these four variables to create two composite variables: one averaging the two *incentives* items (i.e., incentives to help and affiliate with the target), and one averaging the two *willingness*

items (i.e., willingness to help and affiliate with the target). Yet we found that our victim manipulation had a strong effect on our two *helping* items, but an inconsistent effect on our two *affiliation* items. On this basis, we chose to deviate from our plan to create composite variables.

Here, we report analyses of all four individual variables. In Fig. S8, we plot both of our helping and affiliation variables by condition in Experiment 13. And in Table S11, we report results of linear regressions comparing each pair of conditions (victim vs. neutral, accident vs. neutral, and victim vs. accident) for both of our helping and affiliation variables.

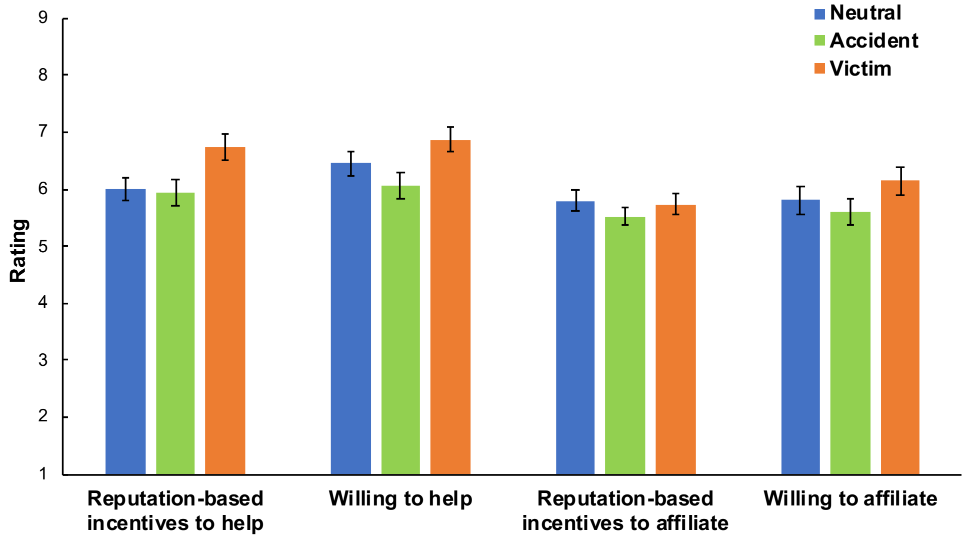

Fig. S8. Helping and affiliation variables across conditions in Experiment 13.

Variable	Victim vs. Neutral (n = 400)	Accident vs. Neutral (n = 400)	Victim vs. Accident (n = 400)
Reputation-based incentives to help	Neutral = 6.00, Victim = 6.74, **b = 0.74, t = 4.73, p < .001**	Neutral = 6.00, Accident = 5.94, **b = -0.06, t = -0.42, p = .677**	Accident = 5.94, Victim = 6.74, **b = 0.81, t = 4.90, p < .001**
Willing to help	Neutral = 6.45, Victim = 6.88, **b = 0.42, t = 2.72, p = .007**	Neutral = 6.45, Accident = 6.06, **b = -0.39, t = -2.50, p = .013**	Accident = 6.06, Victim = 6.88, **b = 0.82, t = 4.96, p < .001**
Reputation-based incentives to affiliate	Neutral = 5.80, Victim = 5.73, **b = -0.07, t = -0.52, p = .603**	Neutral = 5.80, Accident = 5.52, **b = -0.27, t = -2.25, p = .025**	Accident = 5.52, Victim = 5.73, **b = 0.21, t = 1.65, p = .099**
Willing to affiliate	Neutral = 5.81, Victim = 6.14, **b = 0.33, t = 1.87, p = .062**	Neutral = 5.81, Accident = 5.60, **b = -0.21, t = -1.23, p = .220**	Accident = 5.60, Victim = 6.14, **b = 0.54, t = 3.12, p = .002**

Table S11. Analyses of helping and affiliation variables in Experiment 13.

We find that our victim manipulation had a positive effect, relative to both other conditions, on both helping variables. In contrast, effects on our affiliation variables were smaller and less consistent. We do observe a positive effect of the victim condition on willingness to affiliate (that is marginally significant in contrast to the neutral condition, and significant in contrast to the accident condition), but it is smaller than the effect of the victim condition on willingness to help. And when looking at incentives to affiliate, we find no significant effect of the victim condition as compared to the neutral condition (with a directionally negative

coefficient), but a marginally significant positive effect of the victim condition as compared to the accident condition.

Thus, while the effects of our victim manipulation on our affiliation variables mostly go in the same direction as the effects on our helping variables, they are less consistent and weaker. These results suggest that subjects perceive stronger incentives to, and are more willing to, help (as compared to affiliate with) victims. This finding is interesting, and is potentially consistent with the proposal, outlined in the main text, that people perceive elevated incentives to help victims because helping victims is an effective way to signal that one disapproves of the transgression in question (and is thus unlikely to transgress oneself). It makes sense that helping a victim may be a more effective way to signal disapproval of a transgression than generally affiliating with the victim, insofar as helping is perceived as a specific response to the transgression (but general affiliation is not).

Our Experiment 13 pre-registration also included two mediation analyses as primary analyses. Specifically, we planned to test for significant indirect effects of the victim condition on target morality and trustworthiness, using our two helping/affiliation composite variables as mediators. We do not conduct these analyses because, as explained previously, we do not report mediation analyses in our paper. Furthermore, like we noted above in the context of Experiment 11a, the decision to treat willingness to help (a measure of motivation for justice-restorative action) as a mediator of the Virtuous Victim effect assumes a causal model that differs somewhat from the causal model we ultimately propose in our paper. Our paper proposes that the Virtuous Victim effect serves to adaptively boost motivation for justice-restorative action; according to this proposal, motivation for justice-restorative action is a consequence (not a cause) of the Virtuous Victim effect, and thus should not be treated as a mediator.

Finally, we note one last feature of our Experiment 13 pre-registration. It incorrectly states that we measured evaluations of target moral character before measuring our set of helping and affiliation variables; in fact, we measured our set of helping and affiliation variables first.

4.2. Aggregate analyses of secondary variables

The previous section concludes our discussion of "primary" deviations from our pre-registered analysis plans. However, as mentioned above, our pre-registered analysis plans also involved several secondary and/or exploratory analyses that, for reasons of brevity, we do not discuss individually in this document. Instead, here we report aggregate analyses of two secondary dependent variables that we collected across multiple experiments (ratings of sympathy towards, and exploitability of, the target).

In these aggregate analyses, we focus on the basic effect of our victim manipulation, as compared to our neutral condition. To do so, we analyze our "standard set of neutral vs. victim conditions". As described in Section 2.6 of this document, this label refers to the set of data that we analyze in Fig. S1 and Table S3: the standard victim and neutral conditions of all experiments that included both of these conditions, with the exceptions of (i) the first-person conditions of Experiments 5 and 6, or (ii) the "disincentives" conditions of Experiment 11a. (As described in Section 2.1 of this document, these exceptions reflect that we expected that these conditions might meaningfully change the effects of our standard victim manipulation, and our results supported this expectation.)

4.2.1. Sympathy

We begin by discussing our first secondary dependent measure: sympathy towards the target, which we measured in Experiments 1-7, 11a, and 13-14 (using a three-item scale; we report analyses of composite sympathy ratings computed by averaging these three items).

In the main text, we consider the hypothesis that the Virtuous Victim effect occurs because people feel sympathy for victims, and are thus driven to evaluate them positively. According to this hypothesis, the Virtuous Victim effect should be quite general: it should extend to all sorts of positive traits, and apply to anybody who has suffered. Yet we instead find that the Virtuous Victim effect is specific to victims of *moral* transgressions (i.e., it does not extend to victims of accidental misfortune) and to *moral* virtue (i.e., it does not extend equally to positive but nonmoral traits). These results suggest that the effect does not simply reflect that people are generally compelled to positively evaluate those who have suffered (and who thus elicit sympathy).

Importantly, however, these results do *not* rule out the possibility that sympathy is an important part of the psychology surrounding the Virtuous Victim effect. In fact, as mentioned in the main text, analyses of sympathy ratings are consistent with the possibility that sympathy is a proximate cause, or consequence, of the Virtuous Victim effect. Here, we provide these analyses, which show that ratings of sympathy for the target track with the Virtuous Victim effect.

Specifically, recall that subjects see victims of immorality as more moral than neutral targets or victims of accidental misfortune. When looking to sympathy, we find that (i) subjects reported more sympathy for immorality victims, both relative to neutral targets (b = 4.21 [4.10, 4.32], t = 74.43, B = .75, p < .001, n = 4,949 observations across 3,929 unique subjects; data from our standard set of neutral vs. victim conditions, among experiments that measured sympathy) and accident victims (b = 1.36 [1.13, 1.59], t = 11.44, B = .34, p < .001, n = 1,004; data from our two experiments including accident conditions, both of which measured sympathy), and (ii) reported sympathy is positively correlated with ratings of target moral character (B = .30, p < .001, n = 4,949 observations across 3,929 unique subjects; data from our standard set of neutral vs. victim conditions, among experiments that measured sympathy).

Thus, sympathy ratings track with the Virtuous Victim effect—suggesting that sympathy could be an important part of the psychology surrounding the effect. Yet we also find that accident victims receive much more sympathy than neutral targets (b = 3.46 [3.23, 3.69], t = 29.61, B = .69, p < .001, n = 1,006) despite not being rated as having better moral character than neutral targets (b = .03 [-.13, .19], t = .36, B = .01, p = .722, n = 1,006) (data from our two experiments including accident conditions, both of which measured sympathy). This pattern bolsters our argument that the Virtuous Victim effect does *not* simply reflect that people elevate the morality of anybody that they feel sorry for.

Why, then, does sympathy track with the Virtuous Victim effect? One possibility is that seeing immorality victims as virtuous serves to boost our sympathy for them. This proposal is consistent with the Justice Restoration Hypothesis, insofar as feeling elevated sympathy for immorality victims bolsters our motivation to help them and/or punish the perpetrators who have harmed them.

4.2.2. Exploitability

Next, we discuss another secondary dependent measure: ratings of the target's "exploitability".

In the main text, we consider the hypothesis that the Virtuous Victim effect reflects that subjects hold a genuine belief that victims tend to be people who behave morally. If this were true, subjects should rate victims as more likely to behave virtuously (and/or less likely to behave unethically). Yet we instead find that subjects see victims as possessing elevated moral *traits* (i.e., as being more moral and trustworthy people), but do *not* expect victims to behave more morally. These results suggest that the effect does not simply reflect that a genuine belief about the typical conduct of victims.

While we see these results as quite decisive, some of our experiments also tested the "genuine belief" account of the Virtuous Victim effect in another way. In particular, they tested the hypothesis that subjects (i) see victims as people who are easy to exploit, and (ii) believe that exploitable individuals tend to be moral actors. To test this hypothesis, Experiments 1-7 and 14 included a three-item exploitability scale measuring the extent to which subjects saw the target as a person who is easily exploited by others. Additionally, in several of these experiments (specifically, Experiments 2,3,5-7, and 14), we added an item to the scale measuring a related construct: the extent to which subjects saw the target as a trusting person.

When we conduct aggregate exploitability analyses, we find that subjects indeed see victims of immorality as relatively exploitable, both as compared to neutral targets (b = .91[.80, 1.02], t = 15.96, B = .27, p < .001, n = 4,144 observations across 3,124 unique subjects; data from our standard set of neutral vs. victim conditions, among experiments that measured exploitability) and accident victims (b = 1.49 [1.22, 1.76], t = 10.92, B = .41, p < .001, n = 601; data from Experiment 3, our one accident experiment that measured exploitability). And looking to our trusting item, we see the same pattern: subjects see victims of immorality as more trusting, both as compared to neutral targets (b = .38 [.25, .51], t = 5.64, B = .12, p < .001, n = 2,211; data from our standard set of neutral vs. victim conditions, among experiments that included our trusting item) and accident victims (b = .53 [.26, .81], t = 3.84, B = .15, p < .001, n = 601; data from Experiment 3, our one accident experiment that included our trusting item).

Thus, subjects do believe that victims of immorality are exploitable and trusting people. Does this explain why they see victims as moral? Interestingly, we do *not* find a positive correlation between our three-item exploitability scale and ratings of moral character (B = .01, p = .774, n = 4,144 observations across 3,124 unique subjects; data from our standard set of neutral vs. victim conditions, among experiments that measured exploitability and character). We do, however, find a positive correlation between our trusting item and ratings of moral character (B = .46, p < .001, n = 2,211; data from our standard set of neutral vs. victim conditions, among experiments that included our trusting item and measured character). Thus, our analyses of our trusting item are thus consistent with the hypothesis that the Virtuous Victim effect reflects that people infer that victims are more trusting, and believe that trusting individuals tend to be morally virtuous.

Yet we argue that this explanation for the Virtuous Victim effect is nonetheless implausible, given that the Virtuous Victim effect does not extend to predictions about moral behavior. And consistent with this intuition, just like we observe a positive correlation between our trusting item and ratings of moral character, we observe a similarly-sized positive correlation between our trusting item and predicted moral behavior, B = .40, p < .001, n = 804 (as well as a relatively weaker negative correlation between our trusting item and predicted immoral behavior, B = -.15, p < .001, n = 804) (data from our standard set of neutral vs. victim conditions, among experiments that included our trusting item and measured behavior predictions). This finding suggests that people expect trusting individuals to have more positive moral character *and* to

behave more morally. And it thus bolsters the argument that if the Virtuous Victim effect were caused by the belief that victims tend to be trusting people, the effect should have extended to predicted moral behavior.

5. Full experimental methods

Here, we provide detailed design information about each of our experiments, including the pre-test for Experiment 15.

All of our experiments began similarly. In our online experiments, Mturk worker subjects began by entering their Mturk ID, completing a consent form, and learning that they would be reading a story and then answering questions about their impression of characters in the story. In our lab experiment, student subjects began by completing a consent form, entering their participation ID, learning that their session would involve a number of "short, unrelated parts" and then learning that they would begin by reading a story and then answering questions about their impression of characters in the story.

In the subsequent sections, we explain how each experiment continued from this starting point. For each experiment, we provide a design overview, include the full texts of all versions of our vignettes, and report all measures collected. Many of our experiments randomized the gender of the target character (along with all other characters in the vignette); for these experiments, we (i) illustrate our stimuli in the context of the *female* version of the vignette and (ii) specify the alternative names provided in the male version of the vignette.

5.1. Experiment 1

Design overview

Experiment 1 employed a four-condition, between-subject design in which subjects were assigned one of the following conditions: *neutral* (in which the target was a neutral character), *standard victim* (in which the target behaved identically, but was victimized by a perpetrator), *other victim* (in which the target behaved identically and was not victimized, but another character was victimized), or *minimal narrative victim* (in which the target behaved identically and was victimized, but less detail was provided about the perpetrator). We also randomized between-subjects whether the target character (and all other characters in the vignette) was male or female. Experiment 1 used our iPad vignette, in which the relevant transgression is the theft of an iPad.

Vignette text

In all conditions, the vignette began as follows: ***Sarah*** *is a politically moderate college student. Last Wednesday, she and a few of her classmates were studying for a big upcoming exam in the common room of Sarah's dorm suite. At one point, one of Sarah's classmates, Gabrielle, asked to use Sarah's iPad to look something up. Later that day, Sarah relaxed by watching a TV show on her iPad, and then went out for the evening.* This was the entire vignette in the *neutral* condition.

In the *standard victim* condition, the vignette continued: *That evening, while Sarah was out, Gabrielle figured out how to enter her suite. After entering, Gabrielle stole Sarah's iPad from her room. When Sarah noticed that it was missing, she was very upset.*

In the *other victim* condition, the vignette continued: *That evening, Gabrielle figured out how to enter the suite of another classmate, Rachel, who Gabrielle knew also owned an iPad.*

After entering, Gabrielle stole Rachel's iPad from her room. When Rachel noticed that it was missing, she was very upset.

In the *minimal narrative victim* condition, the vignette continued: *That evening, while Sarah was out, Sarah's iPad was stolen from her room. When Sarah noticed that it was missing, she was very upset.*

In the *male* version of the vignette, the names "Sarah", "Gabrielle", and "Rachel" were replaced with "Sam", "Gordon", and "Rob" (and male pronouns were used).

Measures

Subjects then answered a series of questions about characters in the vignette, spread out across several pages. All questions were answered on 1-9 Likert scales (Anchors: 1 – Not at all, 2, 3 – A little bit, 4, 5 – Moderately, 6, 7 – Quite a lot, 8, 9 – Extremely). On all pages, the (identical) vignette was presented before the questions. The first presentation of the vignette was paired with the first set of questions.

Key dependent measures. We began by measuring our key dependent measures: (i) ratings of moral character (morality and trustworthiness), and (ii) a set of behavior predictions (predicted past/future, specific/general, moral/immoral behavior).

Moral character. On the first evaluation page, subjects rated the moral character of the target character, Sarah, across two questions. First, they rated Sarah's *morality* ("How moral of a person is Sarah?") and second, they rated Sarah's *trustworthiness* ("How trustworthy of a person is Sarah?").

Behavior predictions. On the next two evaluation pages, subjects made a set of eight predictions about the target's moral behavior. One page elicited four predictions about past behavior, and one page elicited four predictions about future behavior; the order of these pages was randomized between-subjects. Within each page, subjects always first made two predictions about general behavior (both moral and immoral), and then made two predictions about specific moral behavior (both moral and immoral); we randomized between-subjects whether the questions about moral vs. immoral behavior were presented first.

The questions about past behavior asked subjects: "How frequently do you think Sarah engaged in moral behaviors that benefited others over the past year?" (Past, general, moral), "How frequently do you think Sarah engaged in immoral behaviors that benefited others over the past year?" (Past, general, immoral), "How frequently do you think Sarah volunteered to help others in her life over the past year?" (Past, specific, moral), and "How frequently do you think Sarah lied to others to get she wanted over the past year?" (Past, specific, immoral). The questions about future behavior were identical, except that they were directed towards the next year (e.g., "How frequently do you think Sarah will engage in moral behaviors that benefit others over the next year?" (Future, general, moral)).

Secondary dependent measures. Next, we collected our secondary dependent measures: (i) sympathy towards the target, (ii) exploitability of the target, and (iii) (in all conditions besides *neutral*) blame of the perpetrator. Specifically, across three pages, we collected these three measures; the order of these pages was randomized between-subjects.

Sympathy. The set of sympathy question asked subjects in random order: "How sympathetic do you feel towards Sarah?", "How bad do you feel for Sarah?", and "How sorry for Sarah are you?".

Exploitability. The set of exploitability question asked subjects in random order: "How easy do you think that it would be to exploit Sarah for one's personal gain?", "How naïve of a

person do you think Sarah is?", and "How likely do you think that people are to try to harm Sarah in the future?".

Blame. In the *standard victim* condition, the set of blame question asked subjects in random order: "How bad of a person is Gabrielle for stealing Sarah's iPad?", "How immoral was Gabrielle's choice to steal Sarah's iPad?", and "How blameworthy is Gabrielle for stealing Sarah's iPad?". In the *other victim* condition, these questions were identical, except that instead of referring to Sarah's iPad, they referred to Rachel's iPad. In the *minimal narrative victim* condition, these questions were identical, except that we specified upfront in the instructions that subjects would be answering questions "about the person who stole Sarah's iPad", and then instead of referring to Gabrielle, our questions referred to "this person".

Post-experimental survey

Finally, subjects completed a post-experimental survey in which they (i) answered a simple analogy question and wrote three sentences about their activities and plans for their day (questions that were designed to test English comprehension), and then (ii) answered a set of demographic and survey questions. In particular, we measured age, gender, level of education, income, country of residence, generalized trust in others, belief in a just world, political party affiliation, and social and fiscal conservativism. To measure belief in a just world, we asked subjects to rate their agreement, on 1-7 Likert scales, with two statements: "I feel that people get what they deserve" and "In general, I find society to be fair".

5.2. Experiment 2

Design overview

Experiment 2 employed a two-condition, between-subject design in which subjects were assigned one of the following conditions: *neutral* (in which the target was a neutral character) and *standard victim* (in which the target behaved identically, but was victimized by a perpetrator). We also randomized between-subjects whether the target character (and all other characters in the vignette) was male or female. Experiment 2 used our iPad vignette, in which the relevant transgression is the theft of an iPad.

Vignette text

In all conditions, the vignette began as follows: ***Sarah*** *is a politically moderate college student. Last Wednesday, she and a few of her classmates were studying for a big upcoming exam in the common room of Sarah's dorm suite. At one point, one of Sarah's classmates, Gabrielle, asked to use Sarah's iPad to look something up. Later that day, Sarah relaxed by watching a TV show on her iPad, and then went out for the evening.* This was the entire vignette in the *neutral* condition.

In the *standard victim* condition, the vignette continued: *That evening, while Sarah was out, Gabrielle figured out how to enter her suite. After entering, Gabrielle stole Sarah's iPad from her room. When Sarah noticed that it was missing, she was very upset.*

In the *male* version of the vignette, the names "Sarah" and "Gabrielle" were replaced with "Sam" and "Gordon" (and male pronouns were used).

Measures

Subjects then answered a series of questions about characters in the vignette, spread out across several pages. All questions were answered on 1-9 Likert scales (Anchors: 1 – Not at all, 2, 3 – A little bit, 4, 5 – Moderately, 6, 7 – Quite a lot, 8, 9 – Extremely). On all pages, the (identical) vignette was presented before the questions. The first presentation of the vignette was paired with the first set of questions.

Key dependent measures. We began by measuring our key dependent measures: ratings of moral character (morality and trustworthiness).

Moral character. On the first evaluation page, subjects rated the moral character of the target character, Sarah, across two questions. First, they rated Sarah's *morality* ("How moral of a person is Sarah?") and second, they rated Sarah's *trustworthiness* ("How trustworthy of a person is Sarah?").

Secondary dependent measures. Next, we collected our secondary dependent measures: (i) sympathy towards the target and (ii) exploitability of the target. Specifically, across two pages, we collected these two measures; the order of these pages was randomized between-subjects.

Sympathy. The set of sympathy question asked subjects in random order: "How sympathetic do you feel towards Sarah?", "How bad do you feel for Sarah?", and "How sorry for Sarah are you?".

Exploitability. The set of exploitability question asked subjects in random order: "How trusting do you think Sarah is of others?", "How easy do you think that it would be to exploit Sarah for one's personal gain?", "How naïve of a person do you think Sarah is?", and "How likely do you think that people are to try to harm Sarah in the future?".

Post-experimental survey

After completing these measures, subjects completed a survey in which they answered a set of demographic and survey questions. In particular, subjects reported their age, gender, student status, income, two items measuring their belief in a just world, political party affiliation, the extent to which they are socially, and fiscally, liberal vs. conservative, and if English is their native language. To measure belief in a just world, we asked subjects to rate their agreement, on 1-7 Likert scales, with two statements: "I feel that people get what they deserve" and "In general, I find society to be fair". After completing this set of questions, subjects were directed to complete other parts of the lab experiments (that were used for other research purposes, and are not relevant to or discussed in this paper).

5.3. Experiment 3

Design overview

Experiment 3 employed a four-condition, between-subject design in which subjects were assigned one of the following conditions: *neutral* (in which the target was a neutral character), *standard victim* (in which the target behaved identically, but was victimized by a perpetrator), *accident victim: earthquake* (in which the target behaved identically, but suffered accidental misfortune due to an earthquake), or *accident victim: cat* (in which the target behaved identically, but suffered accidental misfortune due to a stray cat). We also randomized between-subjects whether the target character (and all other characters in the vignette) was male or

female. Experiment 3 used our iPad vignette, in which the relevant transgression is the theft of an iPad.

Vignette text

In all conditions, the vignette began as follows: ***Sarah*** *is a politically moderate college student. Last Wednesday, she and a few of her classmates were studying for a big upcoming exam in the common room of Sarah's dorm suite. Two of these classmates were named Rachel and Gabrielle. At one point, Gabrielle asked to use Sarah's iPad to look something up. Later that day, Sarah relaxed by watching a TV show on her iPad, and then went out for the evening.* This was the entire vignette in the *neutral* condition.

In the *standard victim* condition, the vignette continued: *That evening, while Sarah was out, Gabrielle figured out how to enter her suite. After entering, Gabrielle stole Sarah's iPad from her room. When Sarah noticed that it was missing, she was very upset.*

In the *accident victim: earthquake* condition, the vignette continued: *That evening, while Sarah was out, there was a major and unexpected earthquake. The earthquake knocked Sarah's iPad off the shelf, damaging it irreparably. When Sarah noticed that it was broken, she was very upset*.

In the *accident victim: cat* condition, the vignette continued: *That evening, while Sarah was out, a stray cat managed to find its way into her suite. The cat jumped up on the shelf and knocked Sarah's iPad off, damaging it irreparably. When Sarah noticed that it was broken, she was very upset.*

In the *male* version of the vignette, the names "Sarah", "Gabrielle", and "Rachel" were replaced with "Sam", "Gordon", and "Rob" (and male pronouns were used).

Measures

Subjects then answered a series of questions about characters in the vignette, spread out across several pages. All questions were answered on 1-9 Likert scales (Anchors: 1 – Not at all, 2, 3 – A little bit, 4, 5 – Moderately, 6, 7 – Quite a lot, 8, 9 – Extremely). On all pages, the (identical) vignette was presented before the questions. The first presentation of the vignette was paired with the first set of questions.

Key dependent measures. We began by measuring our key dependent measures: ratings of moral character (morality and trustworthiness) as well as four nonmoral traits (sociability, intelligence, funniness, and athleticism).

Moral character and nonmoral traits. On the first evaluation page, subjects evaluated the target character, Sarah, on six traits in random order. Subjects rated Sarah's *morality* ("How moral of a person is Sarah?"), *trustworthiness* ("How trustworthy of a person is Sarah?"), *sociability* ("How social of a person is Sarah?"), *intelligence* ("How intelligent of a person is Sarah?"), *funniness* ("How funny of a person is Sarah?"), and *athleticism* ("How athletic of a person is Sarah?").

Secondary dependent measures. Next, we collected our secondary dependent measures: (i) sympathy towards the target and (ii) exploitability of the target. Specifically, across two pages, we collected these two measures; the order of these pages was randomized between-subjects.

Sympathy. The set of sympathy question asked subjects in random order: "How sympathetic do you feel towards Sarah?", "How bad do you feel for Sarah?", and "How sorry for Sarah are you?".

Exploitability. The set of exploitability question asked subjects in random order: "How trusting do you think Sarah is of others?", "How easy do you think that it would be to exploit Sarah for one's personal gain?", "How naïve of a person do you think Sarah is?", and "How likely do you think that people are to try to harm Sarah in the future?".

Post-experimental survey

Finally, subjects completed a post-experimental survey in which they (i) answered a simple analogy question and wrote three sentences about their activities and plans for their day (questions that were designed to test English comprehension), and then (ii) answered a set of demographic and survey questions. In particular, subjects reported their age, gender, level of education, income, and country of residence.

5.4. Experiment 4

Design overview

Experiment 4 employed a two-condition, between-subject design in which subjects were assigned one of the following conditions: *neutral* (in which the target was a neutral character), *standard victim* (in which the target behaved identically, but was victimized by a perpetrator).

Experiment 4 differed from all other experiments in that it employed more than one vignette. In Experiment 4, all subjects were assigned to evaluate three different vignettes: specifically, our iPad vignette (in which the relevant transgression is the theft of an iPad), our verbal attack vignette (in which the relevant transgression is the verbal attack of a gun owner), and our sexual aggression vignette (in which the relevant transgression is aggression during a sexual encounter). Subjects were assigned to just one experimental condition (i.e., neutral or standard victim) and thus all three vignettes matched this single condition. We randomized between-subjects the order in which the three vignettes were presented. Furthermore, for our iPad vignette only, we also randomized between-subjects whether the target character (and all other characters in the vignette) was male or female.

Before any vignettes were presented, subjects completed (i) a demographic questionnaire and (ii) a set of survey questions assessing their ideology with respect to various social issues.

Demographic questionnaire

In the demographic questionnaire, we measured subjects' age, gender, level of education, income, country of residence, political party affiliation, social and fiscal conservativism, and belief in a just world. To measure belief in a just world, we asked subjects to rate their agreement, on 1-7 Likert scales, with two statements: "I feel that people get what they deserve" and "In general, I find society to be fair".

Ideology Assessment

In the ideology assessment, we measured subjects' agreement with four sets of statements. These four sets of statements were designed to assess ideology with respect to four social issues: gun control, sexual harassment, climate change, and racism by police officers. Each set involved three statements.

For each statement, we categorized whether agreement reflected a liberal or conservative ideology with respect to the relevant issue. We then reverse-coded statements reflecting liberal ideology to compute a composite measure of conservative ideology for each set of statements.

Below, we list each set of statements, and for each statement indicate whether we categorized agreement as liberal or conservative.

Gun control: “We need increased gun regulation in America” (liberal), “It is morally wrong to own guns” (liberal), and “The right to bear arms is extremely important and must be fiercely protected” (conservative).

Sexual harassment: “All else equal, I usually believe women who say that they’ve been sexually assaulted” (liberal), “I strongly support the #MeToo movement against sexual harassment and sexual assault” (liberal), and “Alleged acts of sexual assault and harassment are not always the man’s fault, if the woman sent mixed signals” (conservative).

Climate change: “Global warming is an enormous threat to our society that is caused by human behavior” (liberal), “It is morally wrong to drive a gas-guzzling SUV, given the threat of global warming” (liberal), and “The scientific evidence for climate change has serious limitations” (conservative).

Racism by police officers: “Racism among police officers is a huge problem in society” (liberal), “All else equal, when a police officer is violent towards an unarmed black person, I blame the police officer” (liberal), and “If police officers are more violent towards black people, it is simply because they engage in more criminal activity” (conservative).

All twelve statements were presented on one page in random order. For each statement, subjects rated their level of agreement on the following scale: (i) strongly disagree, (ii) disagree, (iii) somewhat disagree, (iv) neither agree nor disagree, (v) somewhat agree, (vi) agree, or (vii) strongly agree.

Vignette texts

iPad vignette. In all conditions, the vignette began as follows: *Sarah is a politically moderate college student. Last Wednesday, she and a few of her classmates were studying for a big upcoming exam in the common room of Sarah’s dorm suite. At one point, one of Sarah’s classmates, Gabrielle, asked to use Sarah’s iPad to look something up. Later that day, Sarah relaxed by watching a TV show on her iPad, and then went out for the evening*. This was the entire vignette in the *neutral* condition.

In the *standard victim* condition, the vignette continued: *While she was out, Gabrielle figured out how to enter her suite and stole her iPad. When Sarah noticed that it was missing, she was very upset.*

In the *male* version of the moderate vignette, the names “Sarah” and “Gabrielle” were replaced with “Sam” and “Gordon” (and male pronouns were used).

Verbal attack vignette. In all conditions, the vignette began as follows: *Josh is a politically moderate college student. Growing up, his family kept guns in the home for protection and recreational shooting, and Josh owns a few guns. He doesn’t bring them to campus with him, but some of his classmates know about his guns because Josh often talks about them, and has told stories about using them when he goes home for school vacations. Last Wednesday, one of Josh’s classmates, Chelsea, saw him at a party.*

In the *neutral* condition, the vignette continued: *She started talking with Josh and brought up his and his family's gun ownership*.

In the *standard victim* condition, the vignette continued: *Unprovoked, Chelsea started aggressively criticizing Josh for his and his family’s gun ownership, calling him harsh names in front of everyone and saying that gun ownership is a horrible force in society. Josh was very upset.*

Sexual aggression vignette. In all conditions, the vignette began as follows: *Elizabeth is a politically moderate college student. Last Saturday night, she and a few of her close friends went out to a party at a campus fraternity. While she was there, Elizabeth had a few drinks, and chatted with several guys she knew at the frat. Towards the end of the night, she flirted a bunch with a guy named Chris. Later that night, Chris hooked up with Elizabeth in his room.*

In the *neutral* condition, the vignette continued: *Part way through their encounter, Elizabeth told Chris she wanted to stop hooking up, so they stopped.*

In the *standard victim* condition, the vignette continued: *Part way through their encounter, Elizabeth told Chris she wanted to stop hooking up, but he repeatedly kept trying things. Elizabeth was very upset.*

Measures

Subjects answered a series of questions after each of the three vignettes. All questions were answered on 1-9 Likert scales (Anchors: 1 – Not at all, 2, 3 – A little bit, 4, 5 – Moderately, 6, 7 – Quite a lot, 8, 9 – Extremely). On all pages, the (identical) vignette was presented before the questions. The first presentation of the vignette was paired with the first set of questions.

Below, we print the text of all questions. Several questions that were identical across vignettes, with the exception that the target character's name varied across vignettes; for those questions, in the below text we use "[target]" to stand in for the target character's actual name.

Key dependent measures. We began by measuring our key dependent measures: ratings of moral character (morality and trustworthiness).

Moral character. On the first evaluation page, subjects rated the moral character of the target character across two questions. First, they rated the target character's *morality* ("How moral of a person is [target]?") and second, they rated target character's *trustworthiness* ("How trustworthy of a person is [target]?").

Secondary dependent measures. Next, we collected our secondary dependent measures: (i) sympathy towards the target, (ii) exploitability of the target, and (iii) blame towards the perpetrator (in the *standard victim* condition). Specifically, across three pages, we collected these three measures; the order of these pages was randomized between-subjects.

Sympathy. The set of sympathy question asked subjects in random order: "How sympathetic do you feel towards [target]?", "How bad do you feel for [target]?", and "How sorry for [target] are you?".

Exploitability. The set of exploitability question asked subjects in random order: "How easy do you think that it would be to exploit [target] for one's personal gain?", "How naïve of a person do you think [target] is?", and "How likely do you think that people are to try to harm [target] in the future?".

Blame. In the *iPad* vignette, the set of blame question asked subjects in random order: "How bad of a person is Gabrielle for stealing Sarah's iPad?", "How immoral was Gabrielle's choice to steal Sarah's iPad?", and "How blameworthy is Gabrielle for stealing Sarah's iPad?".

In the *verbal attack* vignette, the set of blame question asked subjects in random order: "How bad of a person is Chelsea for aggressively criticizing Josh?", "How immoral was Chelsea's choice to aggressively criticize Josh?", and "How blameworthy is Chelsea for aggressively criticizing Josh?".

In the *sexual aggression* vignette, the set of blame question asked subjects in random order: "How bad of a person is Chris for repeatedly trying things after Elizabeth said that she wanted to stop?", "How immoral was Chris choice to repeatedly try things after Elizabeth said

that she wanted to stop?", and "How blameworthy is Chris for repeatedly trying things after Elizabeth said that she wanted to stop?".

Post-experimental survey

Finally, subjects completed a post-experimental survey in which they (i) answered a simple analogy question and wrote three sentences about their activities and plans for their day (questions that were designed to test English comprehension), and then (ii) answered a set of survey questions. In particular, subjects answered the following questions: "Do you consider gun control to be a moral issue?", "Do you consider sexual assault and harassment to be a moral issue?", and "Do you consider stealing to be a moral issue?". For each of these questions, subjects responded on the following scale: (i) Not at all, (ii) Slightly, (iii) Moderately, (iv) Much, or (v) Very much.

5.5. Experiment 5

Design overview

Experiment 5 employed a two-by-two, between-subject design. As our first factor, we manipulated whether subjects were assigned to the *neutral* condition (in which the target was a neutral character) or the *standard victim* condition (in which the target behaved identically, but was victimized by a perpetrator). As our second factor, we manipulated whether subjects were assigned to the *first-person* condition (in which the vignette was presented in first-person) or the *third-person* condition (in which the vignette was presented in third-person). Subjects were randomly assigned to one of the four possible conditions that resulted from crossing these manipulations. We also randomized between-subjects whether the target character (and all other characters in the vignette) was male or female. Experiment 5 used our iPad vignette, in which the relevant transgression is the theft of an iPad.

Vignette text

In all conditions, the vignette began with some third-person background information, as follows: ***Sarah*** *is a politically moderate college student.*

In both of the *third-person* conditions, the vignette continued: *Last Wednesday, she and a few of her classmates were studying for a big upcoming exam in the common room of Sarah's dorm suite. At one point, one of Sarah's classmates, Gabrielle, asked to use Sarah's iPad to look something up. Later that day, Sarah relaxed by watching a TV show on her iPad, and then went out for the evening.* This was the entire vignette in the *neutral*, *third-person* condition.

In the *standard victim*, *third-person* condition, the vignette additionally continued: *That evening, while Sarah was out, Gabrielle figured out how to enter her suite. After entering, Gabrielle stole Sarah's iPad from her room. When Sarah noticed that it was missing, she was very upset.*

In both of the *first-person* conditions, the vignette continued: *Recently, Sarah told the following story to a friend: "Last Wednesday, a few of my classmates and I were studying for a big upcoming exam in the common room of my dorm suite. At one point, one of my classmates, Gabrielle, asked to use my iPad to look something up. Later that day, I relaxed by watching a TV show on my iPad, and then went out for the evening."* This was the entire vignette in the *neutral, first-person* condition.

In the *standard victim, first-person* condition, the vignette additionally continued: *"That evening, while I was out, Gabrielle figured out how to enter my suite. After entering, Gabrielle stole my iPad from my room. When I noticed that it was missing, I was very upset."*

In the *male* version of the vignette, the names "Sarah" and "Gabrielle" were replaced with "Sam" and "Gordon" (and male pronouns were used).

Measures

Subjects then answered a series of questions about characters in the vignette, spread out across several pages. All questions were answered on 1-9 Likert scales (Anchors: 1 – Not at all, 2, 3 – A little bit, 4, 5 – Moderately, 6, 7 – Quite a lot, 8, 9 – Extremely). On all pages, the (identical) vignette was presented before the questions. The first presentation of the vignette was paired with the first set of questions.

Key dependent measures. We began by measuring our key dependent measures: ratings of moral character (morality and trustworthiness) as well as two nonmoral traits (intelligence and athleticism).

Moral character and nonmoral traits. On the first evaluation page, subjects evaluated the target character, Sarah, on four traits in random order. Subjects rated Sarah's *morality* ("How moral of a person is Sarah?"), *trustworthiness* ("How trustworthy of a person is Sarah?"), *intelligence* ("How intelligent of a person is Sarah?"), and *athleticism* ("How athletic of a person is Sarah?").

Secondary dependent measures. Next, we collected our secondary dependent measures: (i) sympathy towards the target and (ii) exploitability of the target. Specifically, across two pages, we collected these two measures; the order of these pages was randomized between-subjects.

Sympathy. The set of sympathy question asked subjects in random order: "How sympathetic do you feel towards Sarah?", "How bad do you feel for Sarah?", and "How sorry for Sarah are you?".

Exploitability. The set of exploitability question asked subjects in random order: "How trusting do you think Sarah is of others?", "How easy do you think that it would be to exploit Sarah for one's personal gain?", "How naïve of a person do you think Sarah is?", and "How likely do you think that people are to try to harm Sarah in the future?".

Post-experimental survey

Finally, subjects completed a post-experimental survey in which they (i) answered a simple analogy question and wrote three sentences about their activities and plans for their day (questions that were designed to test English comprehension), and then (ii) answered a set of demographic and survey questions. In particular, subjects reported their age, gender, level of education, income, and country of residence.

5.6. Experiment 6

Design overview

Experiment 6 employed a two-by-two, between-subject design. As our first factor, we manipulated whether subjects were assigned to the *neutral* condition (in which the target was a neutral character) or the *standard victim* condition (in which the target behaved identically, but was victimized by a perpetrator). As our second factor, we manipulated whether subjects were

assigned to the *first-person* condition (in which the vignette was presented in first-person) or the *third-person* condition (in which the vignette was presented in third-person). Subjects were randomly assigned to one of the four possible conditions that resulted from crossing these manipulations. We also randomized between-subjects whether the target character (and all other characters in the vignette) was male or female. Experiment 6 used our idea theft vignette, in which the relevant transgression is the theft of an idea by a co-worker.

Vignette text

In all conditions, the vignette began with some third-person background information, as follows: ***Sarah*** *is a 26-year-old woman who works at an advertising agency. Her job involves working with co-workers to come up with clever advertising slogans. While she and her co-workers often brainstorm in teams, they get individual credit for their ideas and are promoted or fired based on the quality of their individual work. Last week, Sarah was brainstorming slogans for a product with her manager, Gabrielle. Previous advertisements for this product had not been very effective, so it was important to come up with something substantially better. During their brainstorming session, Sarah came up with an idea that she was very excited about, and shared it with Gabrielle, who agreed that it was very promising.*

In both of the *third-person* conditions, the vignette continued: *The day after Sarah came up with her idea, Gabrielle and Sarah both met with the director of their advertising agency, who asked if they had come up with anything. Sarah was about to describe her idea when another co-worker came in to the room to return Sarah's iPhone charger to her*. This was the entire vignette in the *neutral*, *third-person* condition.

In the *standard victim*, *third-person* condition, the vignette additionally continued: *When Sarah briefly stood up to grab the charger, Gabrielle jumped in to tell the director that she had come up with an idea she was really proud of, and then presented Sarah's idea as her own. Sarah was very upset.*

In both of the *first-person* conditions, the vignette continued: *Recently, Sarah told the following story to a friend: "The day after I came up with my idea, Gabrielle and I both met with the director of our advertising agency, who asked if we had come up with anything. I was about to describe my idea when another co-worker came in to the room to return my iPhone charger to me."* This was the entire vignette in the *neutral, first-person* condition.

In the *standard victim, first-person* condition, the vignette additionally continued: *"When I briefly stood up to grab the charger, Gabrielle jumped in to tell the director that she had come up with an idea she was really proud of, and then presented my idea as her own. I was very upset."*

In the *male* version of the vignette, the names "Sarah" and "Gabrielle" were replaced with "Sam" and "Gordon" (and male pronouns were used).

Measures

Subjects then answered a series of questions about characters in the vignette, spread out across several pages. All questions were answered on 1-9 Likert scales (Anchors: 1 – Not at all, 2, 3 – A little bit, 4, 5 – Moderately, 6, 7 – Quite a lot, 8, 9 – Extremely). On all pages, the (identical) vignette was presented before the questions. The first presentation of the vignette was paired with the first set of questions.

Key dependent measures. We began by measuring our key dependent measures: ratings of moral character (morality and trustworthiness) as well as two nonmoral traits (intelligence and athleticism).

Moral character and nonmoral traits. On the first evaluation page, subjects evaluated the target character, Sarah, on four traits in random order. Subjects rated Sarah's *morality* ("How moral of a person is Sarah?"), *trustworthiness* ("How trustworthy of a person is Sarah?"), *intelligence* ("How intelligent of a person is Sarah?"), and *athleticism* ("How athletic of a person is Sarah?").

Secondary dependent measures. Next, we collected our secondary dependent measures: (i) sympathy towards the target, (ii) exploitability of the target, (iii) evaluation of target as angry, unforgiving, negative, and entitled, and (iv) subjects' self-reported belief that events described in vignette were true. Specifically, across four pages, we collected these four measures. On the first two pages, we collected measures (i) and (ii) in a random order, and then on the last two pages we collected measures (iii) and (iv) in a random order.

Sympathy. The set of sympathy questions asked subjects in random order: "How sympathetic do you feel towards Sarah?", "How bad do you feel for Sarah?", and "How sorry for Sarah are you?".

Exploitability. The set of exploitability questions asked subjects in random order: "How trusting do you think Sarah is of others?", "How easy do you think that it would be to exploit Sarah for one's personal gain?", "How naïve of a person do you think Sarah is?", and "How likely do you think that people are to try to harm Sarah in the future?".

Evaluation of target as angry, unforgiving, negative, and entitled. This set of questions asked subjects in random order: "How angry of a person do you think that Sarah is?", "To what extent do you think Sarah is a negative person who likes to complain?", "To what extent do you think Sarah tends to feel entitled to a lot from others?", "How unforgiving of a person do you think Sarah is?".

Belief. We asked subjects one question about their belief that the events described in the vignette were true: "When you read the story about Sarah's meeting with the director, how much did you believe that the events described were true?"

Post-experimental survey

Finally, subjects completed a post-experimental survey in which they (i) answered a simple analogy question and wrote three sentences about their activities and plans for their day (questions that were designed to test English comprehension), and then (ii) answered a set of demographic and survey questions. In particular, subjects reported their age, gender, level of education, income, and country of residence.

5.7. Experiment 7

Design overview

Experiment 7 employed a two-condition, between-subject design in which subjects were assigned one of the following conditions: *neutral* (in which the target was a neutral character) and *standard victim* (in which the target behaved identically, but was victimized by a perpetrator). We also randomized between-subjects whether the target character (and all other characters in the vignette) was male or female. Experiment 7 used our corrupt doctor vignette, in

which (i) the relevant transgression is a doctor harming a patient through corruption, and (ii) the vignette provides morally relevant background information about the target character.

Vignette text

In all conditions, the vignette began as follows: ***Sarah*** *is a 34-year-old who works for United Airlines and lives in the Chicago area. She has been married to her husband, Chris, for a few years, and the two of them are thinking about having their first child soon. Sarah is politically moderate and somewhat religious. In her free time, Sarah likes to run, watch movies, drink wine, and attend social events in her church community. She also enjoys traveling, especially because she gets great flight benefits through United.*

Sarah is described her friends as generally being fun to be around, and having a good sense of humor. On the other hand, they think that she can be a bit self-focused when having a conversation, and can get defensive when criticized, but they do say that she usually comes through when you need her to do a favor.

Sarah was recently diagnosed with Crohn's disease. Last week, she went to the doctor to discuss treatment strategies with Dr. Wilson, who has been her doctor for about three years. She and Dr. Wilson discussed a few potential drug options, and the likelihood of each of the working and causing different side effects, given Sarah's medical history and risk profile. This was the entire vignette in the *neutral* condition.

In the *standard victim* condition, the vignette continued: *One of these drugs is called Prednisone, and Dr. Wilson has a longstanding relationship with the drug company that sells it. Given her medical history, Dr. Wilson suspected that Prednisone would be unlikely to work for Sarah, and also likely to cause her some substantial side effects (such as nausea, vomiting, increased sweating or acne, or trouble sleeping).*

However, because of his history with the drug company, Dr. Wilson lied to Sarah and said that he thought Prednisone would be the best treatment option. Sarah decided to take Prednisone as a result, and ended up finding that the drug did not work, and caused considerable side effects. These side effects were very distressing to Sarah.

In the *male* version of the vignette, the names "Sarah" and "Chris" were replaced with "Sam" and "Christina" respectively.

Measures

Subjects then answered a series of questions about characters in the vignette, spread out across several pages. All questions were answered on 1-9 Likert scales (Anchors: 1 – Not at all, 2, 3 – A little bit, 4, 5 – Moderately, 6, 7 – Quite a lot, 8, 9 – Extremely). On all pages, the (identical) vignette was presented before the questions. The first presentation of the vignette was paired with the first set of questions.

Key dependent measures. We began by measuring our key dependent measures: (i) ratings of moral character (morality and trustworthiness) as well as two nonmoral traits (intelligence and athleticism), and (ii) a set of behavior predictions (for four moral and four immoral behaviors).

Moral character and nonmoral traits. On the first evaluation page, subjects evaluated the target character, Sarah, on four traits in random order. Subjects rated Sarah's *morality* ("How moral of a person is Sarah?"), *trustworthiness* ("How trustworthy of a person is Sarah?"), *intelligence* ("How intelligent of a person is Sarah?"), and *athleticism* ("How athletic of a person is Sarah?").

Behavior predictions. On the next evaluation page, subjects made a set of eight predictions about the target's behavior: four predictions about *moral* behavior and four predictions about *immoral* behavior; the order of these predictions was randomized between-subjects.

The questions about moral behavior asked subjects: "How likely do you think Sarah is to donate to charity at her church?", "How likely do you think Sarah is to volunteer to help a new coworker out at United?", "How likely do you think Sarah is to put a lot of effort into planning a birthday surprise for her husband?", and "How likely do you think Sarah is to pay for dinner when a friend from out of town comes to visit her?".

The questions about immoral behavior asked subjects: "How likely do you think Sarah is to flirt with other men at the gym, despite being married?", "How likely do you think Sarah is to intentionally 'forget' to pay a friend back?", "How likely do you think Sarah is to harshly scold her United coworkers when they make honest mistakes?", and "How likely do you think Sarah is to spread mean gossip about members of her church community?".

Secondary dependent measures. Next, we collected our secondary dependent measures: (i) sympathy towards the target and (ii) exploitability of the target. The order of these two pages was randomized between-subjects.

Sympathy. The set of sympathy question asked subjects in random order: "How sympathetic do you feel towards Sarah?", "How bad do you feel for Sarah?", and "How sorry for Sarah are you?".

Exploitability. The set of exploitability question asked subjects in random order: "How trusting do you think Sarah is of others?", "How easy do you think that it would be to exploit Sarah for one's personal gain?", "How naïve of a person do you think Sarah is?", and "How likely do you think that people are to try to harm Sarah in the future?".

Post-experimental survey

Finally, subjects completed a post-experimental survey in which they (i) answered a simple analogy question and wrote three sentences about their activities and plans for their day (questions that were designed to test English comprehension), and then (ii) answered a set of demographic and survey questions. In particular, subjects reported their age, gender, level of education, income, and country of residence.

5.8 Experiment 8

Design overview

Experiment 8 employed a two-condition, between-subject design in which subjects were assigned one of the following conditions: *neutral* (in which the target was a neutral character) and *standard victim* (in which the target behaved identically, but was victimized by a perpetrator). Experiment 8 used our rape vignette, in which the relevant transgression is a sexual assault. The victim condition of this vignette was sourced from Niemi & Young, 2016, and we adapted the vignette to create a neutral condition.

Vignette text

In all conditions, the vignette began as follows: *Kelly was walking home from the grocery store one night when she ran into her cousin's friend from college, Dave, who was home for the summer and lived in the neighborhood. Kelly was carrying many grocery bags and was right*

outside her apartment. Although they didn't know each other well, Dave recognized Kelly and since she was struggling to carry her many grocery bags, he offered to help her carry them up to the apartment. One of the bags was about to tear, and she gladly allowed him to help.

In *standard victim* condition, the vignette continued: *Once in the apartment, however, Dave closed and locked the door behind him and pushed Kelly against a wall forcefully. She tried pushing him off her but wasn't strong enough. When she tried to scream Dave covered her mouth and threatened her, and shortly after he forced sexual intercourse with her.*

Measures

On the same page as the vignette was presented, subjects rated the moral character of the target character, Kelly, across two questions. First, they rated Kelly's *morality* ("How moral of a person is Kelly?") and second, they rated Kelly's *trustworthiness* ("How trustworthy of a person is Kelly?"). Both questions were answered on 1-9 Likert scales (Anchors: 1 – Not at all, 2, 3 – A little bit, 4, 5 – Moderately, 6, 7 – Quite a lot, 8, 9 – Extremely).

Attention checks

After completing these measures and advancing the page, subjects completed two short attention checks. In the first, subjects read a short vignette about a grocery store employee (*Olivia works at a local grocery store. At the store, Olivia's job is to serve as the cashier. Normally, Olivia works Monday-Friday but does not work weekends. However, last week Olivia's coworker Ben asked her to cover his Saturday shift. So this Saturday, Olivia has to work a 7-hour shift.*). Then, on the next page, we asked subjects to identify, via a multiple-choice question, the employee's job at the grocery store (answer choices: manager, cashier, stocker, customer service, it was not specified in the story; correct answer: cashier).

In the second attention check, subjects were shown a photograph of a meal. Then, on the same page, we asked subjects to identify, via a multiple-choice question, a food item that is probably not included in the meal (answer choices: olives, onion, mustard, relish, ketchup, bread, hotdog; correct answer: olives).

Finally, subjects completed a third attention check at the end of the post-experimental survey described below. This attention check involved completing a simple analogy question (Kitten is to cat as puppy is to _____; correct answer: dog). We note that while Experiments 8 and 9 were the only experiments to include these first two attention checks, many other experiments employed a similar analogy question. However, only in Experiments 8 and 9 did our pre-registered plans for our primary analyses involve excluding subjects who answered this question incorrectly.

Post-experimental survey

After completing the attention checks, subjects completed a brief post-experimental survey, in which they (i) reported their age, gender, race, level of education, income, political party affiliation, and social and fiscal conservativism, and then (ii) answered the aforementioned simple analogy question.

5.9 Experiment 9

Design overview

Experiment 9 employed a two-by-two-by-two, between-subject design. As our first factor, we manipulated whether subjects were assigned to the *neutral* condition (in which the target was a neutral character) or the *standard victim* condition (in which the target behaved identically, but was victimized by a perpetrator). To this end, we used a lightly modified version our iPad theft vignette, in which the relevant transgression is the theft of an iPad.

As our second and third factors, we manipulated the target's race (*White* vs. *Black*) and gender (*female* vs. *male*) via a photograph of the target's face that we paired with the vignette in all conditions. More specifically, each subject was assigned to one race/gender condition (i.e., *White male*, *White female*, *Black male*, or *Black female*) and then was randomly presented with one of three potential target photographs corresponding to their condition. The photograph was presented at the top of the screen, with the vignette underneath.

All photographs were selected from the Chicago Face Database (Ma et al., 2015). We selected photographs depicting a closed mouth smile, and of models who were roughly college-aged (given that the iPad theft vignette is about college students). We also selected photographs that were reasonably well-matched across race/gender conditions on perceived age, attractiveness, and trustworthiness (using norming data from the Chicago Face Database); see Fig. S2 and Table S4 for the photographs and corresponding norming data.

As noted above, we lightly modified our iPad theft vignette for use in Experiment 9. In particular, we attempted to select names for the vignette characters that would sound racially neutral (both in the male and female versions of the vignette). Additionally, because Experiment 9 was conducted during the COVID-19 pandemic, we modified the scenario to avoid any reference to indoor gathering.

We also note also that Experiment 9 included three attention checks and, per our pre-registration, our primary analyses restrict to subjects who passed all three checks.

Vignette text

In all conditions, the vignette began as follows: ***Maya*** *is a politically moderate college student, and her photo is shown above. Last Wednesday,* ***Maya*** *and a few of her classmates met up outside to study for a big upcoming exam. At one point, one of Maya's classmates, Jackie, asked to use Maya's iPad to look something up. Later that day, Maya relaxed by watching a TV show on her iPad, and then went out for the evening.* This was the entire vignette in the *neutral* condition.

In *standard victim* condition, the vignette continued: *That evening, while Maya was out, Jackie figured out how to enter her dorm suite. After entering, Jackie stole Maya's iPad from her room. When Maya noticed that it was missing, she was very upset.*

In the *male* version of the vignette, the names "Maya" and "Jackie" were replaced with "Kevin" and "Michael" (and male pronouns were used).

Measures

On the same page as the vignette was presented, subjects rated the moral character of the target character, Maya, across two questions. First, they rated Maya's *morality* ("How moral of a person is Maya?") and second, they rated Maya's *trustworthiness* ("How trustworthy of a person

is Maya?”). Both questions were answered on 1-9 Likert scales (Anchors: 1 – Not at all, 2, 3 – A little bit, 4, 5 – Moderately, 6, 7 – Quite a lot, 8, 9 – Extremely).

Attention checks

After completing these measures and advancing the page, subjects completed two short attention checks. In the first, subjects read a short vignette about a grocery store employee (*Olivia works at a local grocery store. At the store, Olivia's job is to serve as the cashier. Normally, Olivia works Monday-Friday but does not work weekends. However, last week Olivia's coworker Ben asked her to cover his Saturday shift. So this Saturday, Olivia has to work a 7-hour shift.*). Then, on the next page, we asked subjects to identify, via a multiple-choice question, the employee’s job at the grocery store (answer choices: manager, cashier, stocker, customer service, it was not specified in the story; correct answer: cashier).

In the second attention check, subjects were shown a photograph of a meal. Then, on the same page, we asked subjects to identify, via a multiple-choice question, a food item that is probably not included in the meal (answer choices: olives, onion, mustard, relish, ketchup, bread, hotdog; correct answer: olives).

Finally, subjects completed a third attention check at the end of the post-experimental survey described below. This attention check involved completing a simple analogy question (Kitten is to cat as puppy is to _____; correct answer: dog). We note that while Experiments 8 and 9 were the only experiments to include these first two attention checks, many other experiments employed a similar analogy question; however, only in Experiments 8 and 9 did our pre-registered plans for our primary analyses involve excluding subjects who answered this question incorrectly.

Post-experimental survey

After completing the attention checks, subjects completed a brief post-experimental survey, in which they (i) reported their age, gender, race, level of education, income, political party affiliation, and social and fiscal conservativism, and then (ii) answered the aforementioned simple analogy question.

5.10 Experiment 10

Design overview

Experiment 10 employed a three-condition, between-subjects design in which subjects were assigned to one of the following conditions: *Control* (in which the target was described as relatively less moral and relatively less competent), *moral* (in which the target was described as relatively more moral and relatively less competent), and *competent* (in which the target was described as relatively less moral and relatively more competent). After subjects read about the target’s morality and competence, they were always assigned to the *victim* condition of our idea theft vignette, in which the relevant transgression is the theft of an idea.

Vignette text

In all conditions, the vignette began by describing the target as follows: *Sarah is a 26-year-old woman who works at an advertising agency.*

In the *control* and *competent* conditions (in which the target is described as relatively less moral), the vignette continued: *Sarah is a fun-loving person who enjoys spending time with*

friends and family, although people around her sometimes find her to be self-centered and arrogant. For example, Sarah pretty much never volunteers her time or donates any money to charity, and she tends to talk about herself more than she listens to others. Recently, when Sarah's friend was laid off, Sarah offered to help him with his job search, bragging about her relevant expertise. But when he followed up with a few specific requests, Sarah decided not to respond, reasoning that helping would take up time that she'd rather spend doing something more fun. That said, Sarah's friends and family do still often enjoy her company.

In the *moral* condition (in which the target is described as relatively more moral), the second paragraph of the vignette instead read: *Sarah is a good listener, frequently volunteers to help others out, and often plans thoughtful surprises for friends and family. Recently, when Sarah's friend was laid off, Sarah offered to help him with his job search and spent hours providing him with emotional support. She also surprised him by taking him out to an outdoor dinner at his favorite restaurant and paying for appetizers, dinner, drinks, and dessert. Sarah also regularly donates money to several different charitable organizations and finds way to volunteer her time. For example, she has recently been quite involved in her church group's efforts to fundraise for people in her community who have been negatively affected by coronavirus.*

In the *control* and *moral* conditions (in which the target is described as relatively less competent), the third paragraph of the vignette read: *In her free time, two of Sarah's hobbies are cooking and running, although she is not great at either activity. Even after several attempts making the same dish, the dishes that Sarah cooks often wind up bland or over-cooked. Additionally, Sarah runs fairly slowly and is unable to complete long distance runs.*

In the *competent* condition (in which the target is described as relatively more competent), the third paragraph of the vignette instead read: *In her free time, two of Sarah's hobbies are cooking and running. Sarah is exceptionally skilled at both of these activities. Sarah frequently cooks very complex dishes and pretty much everything that she makes turns out delicious. Despite the fact that Sarah has zero professional training, many of her dishes are easily restaurant-quality. Additionally, Sarah is a very fast runner who regularly runs in half-marathons, and has actually placed in some major races in the past.*

After reading this description of the target, subjects advanced to a new page. Then, all subjects read the *victim* version of our idea theft vignette, spread across two pages.

On the first page, subjects read: *At work, Sarah's job involves working with co-workers to come up with clever advertising slogans. While she and her co-workers often brainstorm in teams, they get individual credit for their ideas and are promoted or fired based on the quality of their individual work. Last week, Sarah was brainstorming slogans for a product with her manager, Gabrielle. Previous advertisements for this product had not been very effective, so it was important to come up with something substantially better. During their brainstorming session, Sarah came up with an idea that she was very excited about, and shared it with Gabrielle, who agreed that it was very promising.*

On the second page, subjects read: *The day after Sarah came up with her idea, Gabrielle and Sarah both met with the director of their advertising agency, who asked if they had come up with anything. Sarah was about to describe her idea when another co-worker came in to the room to return Sarah's iPhone charger to her. When Sarah briefly stood up to grab the charger, Gabrielle jumped in to tell the director that she had come up with an idea she was really proud of, and then presented Sarah's idea as her own. Sarah was very upset.*

Measures

Next, subjects again advanced to a new page and began answering a series of questions about the vignette, spread out across several pages. All questions were answered on 1-9 Likert scales (Anchors: 1 – Not at all, 2, 3 – A little bit, 4, 5 – Moderately, 6, 7 – Quite a lot, 8, 9 – Extremely). On all pages, the full (identical) vignette was presented before the questions (including the background information about the target's morality and competence).

Morality and competence. On the first evaluation page, subjects evaluated the victim character, Sarah, on two traits in random order; these measures served as manipulation checks. Subjects rated Sarah's *morality* ("How moral of a person is Sarah?") and Sarah's *competence* ("How competent of a person is Sarah?").

Willingness to punish. On the second evaluation page, we measured our first key dependent variable: willingness to punish. Subjects were presented with the following question: "Imagine that you work at the advertising agency with Sarah and Gabrielle. How inclined would you be to "punish" Gabrielle (e.g., by expressing your disapproval of Gabrielle's behavior to others, giving Gabrielle the cold shoulder at work, reporting Gabrielle's behavior formally, or taking some other similar action)?"

Willingness to help. On the third evaluation page, we measured our second key dependent variable: willingness to help. Subjects were presented with the following question: "Imagine that you work at the advertising agency with Sarah and Gabrielle. How willing would you be to help Sarah (e.g., by helping Sarah to brainstorm new ideas, being especially warm and friendly towards Sarah at work, making sure to praise Sarah to others for her work, or taking other similar action)?"

Post-experimental survey

Finally, subjects completed a post-experimental survey in which they (i) answered a simple analogy question (that was designed to test English comprehension), and then (ii) answered a set of demographic and survey questions (in which they reported their age, gender, level of education, income, and political party affiliation).

5.11. Experiment 11a

Design overview

Experiment 11a employed a two-by-two, between-subject design. As our first factor, we manipulated whether subjects were assigned to the *neutral* condition (in which the target was a neutral character) or the *standard victim* condition (in which the target behaved identically, but was victimized by a perpetrator). Experiment 11a used our idea theft vignette, in which the relevant transgression is the theft of an idea by a co-worker.

As our second factor, we manipulated whether subjects were assigned to the *incentives* condition (in which, before reading the vignette, subjects were asked to imagine being in a situation that created incentives for justice-restorative action) or the *disincentives* condition (in which subjects were asked to imagine being in a situation that created *disincentives* for justice-restorative action). Subjects were randomly assigned to one of the four possible conditions that resulted from crossing these manipulations.

Imagination exercise

We began the experiment by asking subjects to engage in an imagination exercise, which we used to manipulate the presence of incentives versus disincentives for justice-restorative action.

In all conditions, subjects were given the following instructions: *Imagine that you work at an advertising agency. At the agency, you and your co-workers work to come up with clever advertising slogans. While you and your co-workers often brainstorm in teams, everyone gets individual credit for their ideas and are promoted or fired based on the quality of their individual work.*

In the *incentives* conditions, the instructions continued: *In your experience, one problem with this system is that it creates the potential for people to steal each other's ideas. When this happens, the wrong person gets credit for an idea, which you think is really unfair and wrong. While some people at work agree with you that this is a major issue, other colleagues of yours tend to dismiss it as not being a big concern. They acknowledge that people do sometimes steal ideas, leading to an unjust outcome. However, they argue that idea stealing is extremely rare. They also argue that the rest of the time, the system works really well for creating productive brainstorming sessions and generating excellent ideas. You, however, strongly disagree. You think that ideas are frequently stolen, and that the system needs major reform. At work, you've been very vocal about your views on this issue, and have publicly argued for your position to colleagues on several occasions.*

In the *disincentives* conditions, the instructions continued: *One problem that some people at work have with this system is that it creates the potential for people to steal each other's ideas. When this happens, the wrong person gets credit for an idea, which they think is really unfair and wrong. While some people at work think this is a major issue, you (and some other colleagues of yours) strongly disagree. You acknowledge that people do sometimes steal ideas, leading to an unjust outcome. However, in your experience, idea stealing is extremely rare. And the rest of the time, the system works really well for creating productive brainstorming sessions and generating excellent ideas. Since you don't think that ideas are frequently stolen, you don't think that the system needs reform. At work, you've been very vocal about your views on this issue, and have publicly argued for your position to colleagues on several occasions.*

Next, subjects in both conditions were presented with the following prompt: *Imagine that a co-worker asked you whether you think that idea stealing is a big concern with the current system at work. In your own words, what would you tell him or her?* Subjects were asked to write two sentences explaining their position, and were provided with a free text box to do so.

Vignette text

After subjects completed this writing exercise, they advanced to a new page. On this page, we presented subjects with the vignette.

In all conditions, the vignette began as follows: *One of your co-workers is a 26-year-old woman named* ***Sarah***. *Last week, Sarah was brainstorming slogans for a product with her manager, Gabrielle. Previous advertisements for this product had not been very effective, so it was important to come up with something substantially better. During their brainstorming session, Sarah came up with an idea that she was very excited about, and shared it with Gabrielle, who agreed that it was very promising.*

In the *neutral* conditions, the vignette continued: *Right after Sarah came up with her idea, Sarah ran into the director of the advertising agency, who asked if she and Gabrielle had come up with anything. Sarah told her director about her idea.*

In the *standard victim* conditions, the vignette continued: *Right after Sarah came up with her idea, Gabrielle and Sarah both ran into the director of the advertising agency, who asked if they had come up with anything. Before Sarah could say anything, Gabrielle jumped in to tell the director that she had come up with an idea she was really proud of, and then presented Sarah's idea as her own. Sarah was very upset.*

Measures

Next, subjects again advanced to a new page and began answering a series of questions about characters in the vignette, spread out across several pages. With one exception (noted below), all questions were answered on 1-9 Likert scales (Anchors: 1 – Not at all, 2, 3 – A little bit, 4, 5 – Moderately, 6, 7 – Quite a lot, 8, 9 – Extremely). On all pages, the (identical) vignette was presented before the questions (not including the imagination exercise).

Initial secondary dependent measure. We began by measuring one of our secondary dependent measures: ratings of moral character (morality and trustworthiness) of the "other person", Gabrielle (who was a neutral other character in the *neutral* condition, and the perpetrator in the *standard victim* condition).

"Other person" moral character. On the first evaluation page, subjects rated Gabrielle's character across two questions in random order. They rated Gabrielle's *morality* ("How moral of a person is Gabrielle?") and *trustworthiness* ("How trustworthy of a person is Gabrielle?").

Key dependent measures. Next, we measured measuring our key dependent measures: ratings of moral character (morality and trustworthiness) of the target, Sarah.

Target moral character. On the second evaluation page, subjects rated Sarah's character across two questions in random order. They rated Sarah's *morality* ("How moral of a person is Sarah?") and *trustworthiness* ("How trustworthy of a person is Sarah?").

Other secondary dependent measures. Next, we collected the rest of our secondary dependent measures, which involved questions concerning: (i) sympathy towards the target, (ii) helping the target, (iii) punishing the other person, (iv) blame of the other person, and (v) anger towards the other person. Each measure was assessed on a separate page. We first presented measures (i), (ii), and (iii) in a random order to all subjects. Then, we presented measures (iv) and (v) in a random order to subjects in the *standard victim* conditions only.

Sympathy. The set of sympathy questions asked subjects in random order: "How sympathetic do you feel towards Sarah?", "How bad do you feel for Sarah?", and "How sorry for Sarah are you?".

Helping the target. The set of "helping the target" questions asked subjects in random order: "If Sarah needed help at work, how willing would you be to help her out?" and "If you helped Sarah out, how positively would this be seen by your co-workers?".

Punishing the other person. The set of "punishing the other person" questions asked subjects in random order: "How inclined would you be to give Gabrielle the cold shoulder?" and "If you gave Gabrielle the cold shoulder, how would this influence your reputation in the eyes of your co-workers?". For this second question, the anchors on the 1-9 Likert scale differed from the anchors for all other questions. Specifically, they read: 1 – It would HURT your reputation a lot, 2, 3 – It would HURT your reputation a little, 4, 5 – It would have no effect on your

reputation, 6, 7 – It would HELP your reputation a little, 8, 9 – It would HELP your reputation a lot.

Blame of the other person. The set of "blame of the other person" questions asked subjects in random order: "How bad of a person is Gabrielle for her behavior towards Sarah?", "How bad was Gabrielle's behavior towards Sarah?", and "How blameworthy is Gabrielle for her behavior towards Sarah?".

Anger towards the other person. The set of "anger towards the other person" questions asked subjects in random order: "How angry do you feel towards Gabrielle for her behavior towards Sarah?", "How much of an outrage is Gabrielle's behavior?", and "How upset are you with Gabrielle?".

Post-experimental survey

Finally, subjects completed a post-experimental survey in which they (i) answered a set of demographic and survey questions (in which they reported their age, gender, level of education, income, political party affiliation, and social and fiscal conservativism), and then (ii) answered a simple analogy question (that was designed to test English comprehension).

5.12. Experiment 11b

Design overview

Experiment 11b employed a two-condition, between-subject design in which subjects were assigned to one of the following conditions: *incentives* (in which subjects read about the situation described in the "incentives" condition of Experiment 11a) and *disincentives* (in which subjects read about the situation described in the "disincentives" condition of Experiment 11a). After subjects read about the relevant scenario, they were always assigned to the *victim* condition of our idea theft vignette, in which the relevant transgression is the theft of an idea.

Imagination exercise

Like in Experiment 11a, we began by asking subjects to engage in an imagination exercise. However, in contrast to Experiment 11a, the focal actor in the imagined situation was not the subject, but rather a person named James.

In all conditions, subjects were given the following instructions: *Imagine that a man named James works at an advertising agency. At the agency, James and his co-workers work to come up with clever advertising slogans. While James and his co-workers often brainstorm in teams, everyone gets individual credit for their ideas and are promoted or fired based on the quality of their individual work.*

In the *incentives* condition, the instructions continued: *In James's experience, one problem with this system is that it creates the potential for people to steal each other's ideas. When this happens, the wrong person gets credit for an idea, which James thinks is really unfair and wrong. While some people at work agree with James that this is a major issue, other colleagues of his tend to dismiss it as not being a big concern. They acknowledge that people do sometimes steal ideas, leading to an unjust outcome. However, they argue that idea stealing is extremely rare. They also argue that the rest of the time, the system works really well for creating productive brainstorming sessions and generating excellent ideas. James, however, strongly disagrees. He thinks that ideas are frequently stolen, and that the system needs major*

reform. At work, James has been very vocal about his views on this issue, and has publicly argued for his position to colleagues on several occasions.

In the *disincentives* condition, the instructions continued: *One problem that some people at work have with this system is that it creates the potential for people to steal each other's ideas. When this happens, the wrong person gets credit for an idea, which they think is really unfair and wrong. While some people at work think this is a major issue, James (and some other colleagues of his) strongly disagree. James acknowledges that people do sometimes steal ideas, leading to an unjust outcome. However, in his experience, idea stealing is extremely rare. And the rest of the time, the system works really well for creating productive brainstorming sessions and generating excellent ideas. Since James doesn't think that ideas are frequently stolen, he doesn't think that the system needs reform. At work, James has been very vocal about his views on this issue, and has publicly argued for his position to colleagues on several occasions.*

Next, subjects in both conditions were presented with the following prompt: *Imagine that a co-worker asks James whether he thinks that idea stealing is a big concern with the current system at work. What do you think James would you tell this co-worker?* Subjects were asked to write two sentences explaining their position, and were provided with a free text box to do so.

Vignette text

After subjects completed this writing exercise, they advanced to a new page. On this page, we presented subjects with the *victim* version of the idea theft vignette.

The vignette read: *One of James's co-workers is a 26-year-old woman named Sarah. Last week, Sarah was brainstorming slogans for a product with her manager, Gabrielle. Previous advertisements for this product had not been very effective, so it was important to come up with something substantially better. During their brainstorming session, Sarah came up with an idea that she was very excited about, and shared it with Gabrielle, who agreed that it was very promising. Right after Sarah came up with her idea, Gabrielle and Sarah both ran into the director of the advertising agency, who asked if they had come up with anything. Before Sarah could say anything, Gabrielle jumped in to tell the director that she had come up with an idea she was really proud of, and then presented Sarah's idea as her own. Sarah was very upset.*

Measures

Next, subjects again advanced to a new page and began answering a series of questions about characters in the vignette. Across three different pages, we collected three key dependent variables: (i) incentives to punish, (i) incentives to help, and (iii) victim ratings. We both randomized (i) whether victim ratings were measured first or last, and (ii) whether incentives to punish were measured before or after incentives to help. On all pages, the full (identical) vignette was presented before the questions (including the imagination exercise). All questions were answered on 1-9 Likert scales.

Incentives to punish. To measure subjects' evaluations of James' incentives to punish the perpetrator, we presented subjects with the following preamble: "Now, imagine that James is considering "punishing" Gabrielle for presenting Sarah's idea as her own. For example, James is considering telling other people what Gabrielle did to Sarah and expressing his disapproval of Gabrielle's behavior, giving Gabrielle the cold shoulder when he sees her at work, reporting Gabrielle's behavior formally, or taking some other similar action. To what extent would punishing Gabrielle..."

This preamble was followed by two questions, in the following fixed order: (i) "...help James to achieve his goals?" (1 – It would strongly hurt his goals, 2, 3, 4, 5 – It would be neutral, 6, 7, 8, 9 – It would strongly help his goals); (ii) "...be in James's self interest?" (1 – It would strongly be against his interests, 2, 3, 4, 5 – It would be neutral, 6, 7, 8, 9 – It would strongly be in his interests).

Incentives to help. To measure subjects' evaluations of James' incentives to help the victim, we presented subjects with the following preamble: "Now, imagine that James is considering helping Sarah. For example, James is considering helping Sarah to brainstorm new ideas, being especially warm and friendly towards Sarah when he sees her at work, making sure to praise Sarah for her work in front of others, or taking other similar action. To what extent would helping Sarah..." This preamble was followed by the same two questions used to measure incentives to punish.

Victim rating. To measure the extent to which subjects, taking James' perspective, saw the target of idea theft as a victim, we presented subjects with the one question. In the *incentives* condition, it read: "Imagine that James is correct in his assessment that idea theft occurs frequently, such that the brainstorming system needs major reform. To what extent do you think Sarah is a victim of wrongdoing?" (1 – Sarah is not at all a victim, 2, 3, 4, 5 – Sarah is somewhat a victim, 6, 7, 8, 9 – Sarah is very much a victim).

In the *disincentives* condition, the question used identical anchors but instead read: "Imagine that James is correct in his assessment that idea theft is extremely rare, and the brainstorming system otherwise works really well. To what extent do you think Sarah is a victim of wrongdoing?"

Post-experimental survey

Finally, subjects completed a post-experimental survey in which they (i) answered a set of demographic and survey questions (in which they reported their age, gender, level of education, income, political party affiliation, and social and fiscal conservativism), and then (ii) answered a simple analogy question (that was designed to test English comprehension).

5.13. Experiment 12

Design overview

Experiment 12 employed a three-condition, between-subject design in which subjects were assigned one of the following conditions: *neutral* (in which the target was a neutral character), *standard victim* (in which the target behaved identically, but was victimized by a perpetrator), or *other victim* (in which the target behaved identically and was not victimized, but another character was victimized). Experiment 12 used our iPad vignette, in which the relevant transgression is the theft of an iPad.

Experiment 12 differed from all other experiments in that it took place in the context of a hypothetical economic "Trust Game". Specifically, we began the experiment by asking subjects to imagine participating in a Trust Game with another Mturk worker, in the role of the first-mover (who must decide how much money to entrust the second-mover with). We then presented our iPad vignette (in the relevant condition), and framed it as an opportunity for the subject to learn more about the Trust Game second-mover. Specifically, we told subjects that the target character from the vignette was the Trust Game second-mover. And then in addition to asking subjects to rate the moral character of (and answer other questions about) the target

character (like in other experiments), in Experiment 12 we also asked subjects to decide how much to send the target, and to predict how much the target would return, in the Trust Game.

Trust Game

We began Experiment 12 by informing subjects that they would be playing a hypothetical interactive game with another Mturk worker. We asked subjects to imagine that the game was real and was being played for real money, and informed subjects that on subsequent screens we would describe the game as such (despite the game actually being hypothetical).

Next, we described an economic Trust Game to subjects. We told subjects that they would receive an endowment of money and decide how much (if anything) to send to another Mturk worker. Any money sent would be tripled before being delivered to this other Mturk worker, who would then decide how much (if anything) to return to the subject. Specifically, we provided the following instructions to subjects:

> In addition to the payment you will receive for completing this HIT, you have the opportunity to earn more money as bonus, as follows:
>
> You will participate in an interactive game with another Mturk worker. This game involves two players: the Sender and the Receiver.
>
> You will be the "Sender." This other person will be the "Receiver."
>
> In this game:
>
> • You (the Sender) start with 30 cents.
> • You then choose how many cents, if any, to send to the Receiver.
> • Any money you send to the Receiver is tripled: for every 1 cent you send, the Receiver will receive 3 cents.
> • The Receiver then chooses how many cents, if any, to return to you. The Receiver can return nothing, any intermediate amount, or everything.
>
> If you send all 30 cents, the Receiver will receive 90 cents. If the Receiver returns half of the 90 cents, you and the Receiver will both earn 45 cents, and will both have more than you started with.
>
> But if the Receiver returns nothing, he or she will earn 90 cents, while you will earn nothing.
>
> So, you can gain money or lose money by sending money to the Receiver, depending on how much the Receiver returns.
>
> Please answer the following questions, to make sure you understand the second game. You MUST answer ALL questions correctly to receive your bonus!

Next, we asked subjects to answer a set of comprehension questions about the payoff structure of the economic Trust Game:

Imagine that you are deciding how much to send to the Receiver.

Which decision will result in **you** earning the highest payoff?

- ○ You deciding to send nothing
- ○ You deciding to send everything
- ○ It depends on how much the Receiver decides to return to you

Imagine that you are deciding how much to send to the Receiver.

Which decision will result in **the Receiver** earning the highest payoff?

- ○ You deciding to send nothing
- ○ You deciding to send everything
- ○ It depends on how much the Receiver decides to return to you

Imagine that the Receiver is deciding how much to return to you.

Which decision will result in **the Receiver** earning the highest payoff?

- ○ The Receiver deciding to return nothing
- ○ The Receiver deciding to return everything
- ○ It depends on how much you sent to the Receiver

Imagine that the Receiver is deciding how much to return to you.

Which decision will result in **you** earning the highest payoff?

- ○ The Receiver deciding to return nothing
- ○ The Receiver deciding to return everything
- ○ It depends on how much you sent to the Receiver

>>

Correct answers: It depends on how much the Receiver decides to return to you; You deciding to send everything; The Receiver deciding to return nothing; The Receiver deciding to return everything.

Vignette text

Next, we informed subjects that before deciding how much money to send the Receiver in the Trust Game, they would have the opportunity to learn more about them. We informed subjects that Receiver is another Mturk worker named Sarah, and then presented our vignette.

In the *neutral* and *standard victim* conditions, the vignette began as follows: ***Sarah*** *is a politically moderate college student. A few weeks ago, she and a few of her classmates were studying for a big upcoming exam in the common room of Sarah's dorm suite. Two of these classmates were named Rachel and Gabrielle.*

In the *neutral* condition, the vignette continued: *At one point, Gabrielle asked to use Sarah's iPad to look something up. Later that day, Sarah relaxed by watching a TV show on her iPad, and then went out for the evening.*

In the *standard victim* condition, the vignette continued: *At one point, Gabrielle asked to use Sarah's iPad to look something up. Later that day, Sarah relaxed by watching a TV show on her iPad, and then went out for the evening. That evening, while Sarah was out, Gabrielle*

figured out how to enter her suite. After entering, Gabrielle stole Sarah's iPad from her room. When Sarah noticed that it was missing, she was very upset.

In the *other victim* condition, the vignette continued: *At one point, Gabrielle asked to look at Rachel's wristwatch to check the time. Later that day, Sarah relaxed by watching a TV show on her iPad, and then went out for the evening. That evening, Gabrielle figured out how to enter Rachel's suite. After entering, Gabrielle stole Rachel's jewelry box, filled with many expensive items, from her room. When Rachel noticed that it was missing, she was very upset.*

Measures

Key dependent measures. We began by measuring our three key dependent measures, in the following order: (i) money sent to the target in the Trust Game, (ii) predicted money returned by the target in the Trust Game, and (iii) ratings of moral character (morality and trustworthiness). We collected these three measures across three pages. The (identical) vignette was presented on each page before the measure. The first presentation of the vignette was paired with the first measure.

Money sent to the target. First, we asked subjects to decide how much to send to Sarah in the Trust Game. We briefly reminded subjects of their game decision (reiterating that they had received 30 cents, and that for every 1 cent they sent, Sarah would receive 3 cents) and then asked subjects how many cents they would like to send. We provided subjects with choices ranging from 0 to 30 cents in 5-cent increments.

Predicted money returned by the target. Second, we asked subjects to predict how much Sarah would return to them in the Trust Game. We informed subjects that we would ask Sarah to decide what percentage to return of whatever money she receives, and that we would require Sarah to decide on this percentage before learning how much she had been sent. We then asked subjects what percentage they expected Sarah to choose to return. We provided choices ranging from 0% to 100% in 10-point increments.

Moral character. Third, we asked subjects to rate Sarah's moral character, across two questions. First, they rated Sarah's *morality* ("How moral of a person is Sarah?") and second, they rated Sarah's *trustworthiness* ("How trustworthy of a person is Sarah?"). These questions were answered on 1-9 Likert scales (Anchors: 1 – Not at all, 2, 3 – A little bit, 4, 5 – Moderately, 6, 7 – Quite a lot, 8, 9 – Extremely)

Post-experimental survey

Finally, subjects completed a post-experimental survey in which they (i) answered a simple analogy question and wrote three sentences about their activities and plans for their day (questions that were designed to test English comprehension), and then (ii) answered a set of demographic and survey questions. In particular, subjects reported their age, gender, level of education, income, and country of residence.

5.14. Experiment 13

Design overview

Experiment 13 employed a three-condition, between-subject design in which subjects were assigned one of the following conditions: *neutral* (in which the target was a neutral character), *standard victim* (in which the target behaved identically, but was victimized by a perpetrator), or *accident victim: earthquake* (in which the target behaved identically, but suffered

accidental misfortune due to an earthquake). We also randomized between-subjects whether the target character (and all other characters in the vignette) was male or female. Experiment 13 used our iPad vignette, in which the relevant transgression is the theft of an iPad.

Vignette text

In all conditions, the vignette began as follows: ***Sarah*** *is a politically moderate college student. Last Wednesday, she and a few of her classmates were studying for a big upcoming exam in the common room of Sarah's dorm suite. Two of these classmates were named Rachel and Gabrielle. At one point, Gabrielle asked to use Sarah's iPad to look something up. Later that day, Sarah relaxed by watching a TV show on her iPad, and then went out for the evening*. This was the entire vignette in the *neutral* condition.

In the *standard victim* condition, the vignette continued: *That evening, while Sarah was out, Gabrielle figured out how to enter her suite. After entering, Gabrielle stole Sarah's iPad from her room. When Sarah noticed that it was missing, she was very upset.*

In the *accident victim: earthquake* condition, the vignette continued: *That evening, while Sarah was out, there was a major and unexpected earthquake. The earthquake knocked Sarah's iPad off the shelf, damaging it irreparably. When Sarah noticed that it was broken, she was very upset.*

In the *male* version of the vignette, the names "Sarah" and "Gabrielle" were replaced with "Sam" and "Gordon" (and male pronouns were used).

Measures

Subjects then answered a series of questions about characters in the vignette, spread out across several pages. All questions were answered on 1-9 Likert scales (Anchors: 1 – Not at all, 2, 3 – A little bit, 4, 5 – Moderately, 6, 7 – Quite a lot, 8, 9 – Extremely). On all pages, the (identical) vignette was presented before the questions. The first presentation of the vignette was paired with the first set of questions.

Key dependent measures and first secondary dependent measure. We began by measuring our key dependent measures: (i) a set of questions about helping the target and (ii) ratings of target moral character (morality and trustworthiness). And while presenting our set of questions about helping the target, we also presented a set of questions about affiliating with the target. This set of questions constituted our first secondary dependent measure.

Helping and affiliation questions. On the first evaluation page, subjects answered a set of questions about helping and affiliating with the target. Specifically, we asked the following four questions in random order: "If Sarah needed help, how willing would you be to help her out?", "If you helped Sarah out, how positively would this be seen by others?", "How inclined would you be to hang out with Sarah?", and "How would hanging out with Sarah influence your reputation in the eyes of others?".

Moral character. On the next evaluation page, subjects rated the moral character of the target character, Sarah, across two questions in random order. They rated Sarah's *morality* ("How moral of a person is Sarah?") and Sarah's *trustworthiness* ("How trustworthy of a person is Sarah?").

Other secondary dependent measure. Next, we collected our other secondary dependent measure: sympathy towards the target.

Sympathy. The set of sympathy question asked subjects in random order: “How sympathetic do you feel towards Sarah?”, “How bad do you feel for Sarah?”, and “How sorry for Sarah are you?”.

Post-experimental survey

Finally, subjects completed a post-experimental survey in which they (i) answered a simple analogy question and wrote three sentences about their activities and plans for their day (questions that were designed to test English comprehension), and then (ii) answered a set of demographic and survey questions. In particular, subjects reported their age, gender, level of education, income, and country of residence.

5.15. Experiment 14

Design overview

Experiment 14 employed a two-condition, between-subject design in which subjects were assigned one of the following conditions: *neutral* (in which the target was a neutral character) and *standard victim* (in which the target behaved identically, but was victimized by a perpetrator). We also randomized between-subjects whether the target character (and all other characters in the vignette) was male or female. Experiment 14 used our iPad vignette, in which the relevant transgression is the theft of an iPad, but modified the “victim” condition of the vignette to avoid stating that the target was upset following their victimization.

Vignette text

In all conditions, the vignette began as follows: ***Sarah*** *is a politically moderate college student. Last Wednesday, she and a few of her classmates were studying for a big upcoming exam in the common room of Sarah’s dorm suite. At one point, one of Sarah’s classmates, Gabrielle, asked to use Sarah’s iPad to look something up. Later that day, Sarah relaxed by watching a TV show on her iPad, and then went out for the evening.*

This was the entire vignette in the *neutral* condition. In the *standard victim* condition, the vignette continued: *While Sarah was out, Gabrielle figured out how to enter her suite, and stole her iPad.*

In the *male* version of the vignette, the names “Sarah” and “Gabrielle” were replaced with “Sam” and “Gordon” respectively.

Measures

Subjects then answered a series of questions about characters in the vignette, spread out across several pages. All questions were answered on 1-9 Likert scales (Anchors: 1 – Not at all, 2, 3 – A little bit, 4, 5 – Moderately, 6, 7 – Quite a lot, 8, 9 – Extremely). On all pages, the (identical) vignette was presented before the questions. The first presentation of the vignette was paired with the first set of questions.

Key dependent measures. We began by measuring our key dependent measures: (i) a set of behavior predictions (for four moral and four immoral behaviors) and (ii) ratings of moral character (morality and trustworthiness) as well as two nonmoral traits (intelligence and athleticism).

Behavior predictions. Across the first two evaluation pages, subjects made a set of eight predictions about the target’s moral behavior: four predictions about *moral* behavior (on one

page) and four predictions about *immoral* behavior (on another page). The order of these pages was randomized between-subjects, as was the order of predictions within each page.

The questions about moral behavior asked subjects: “How likely do you think Sarah is to donate to charity?”, “How likely do you think Sarah is to volunteer to tutor low-income middle school students for free?”, “How likely do you think Sarah is to help a friend move to an off-campus apartment?”, and “How likely do you think Sarah is to give leftover food to a homeless person?”.

The questions about immoral behavior asked subjects: “How likely do you think Sarah is to flirt with others while in a committed relationship?”, “How likely do you think Sarah is to intentionally ‘forget’ to pay a friend back?”, “How likely do you think Sarah is to exclude or betray a friend in order to fit in with popular peers?”, and “How likely do you think Sarah is to spread private or mean gossip about others?”. The order of the questions was randomized between-subjects.

Moral character and nonmoral traits. On the next evaluation page, subjects evaluated the target character, Sarah, on four traits in random order. Subjects rated Sarah’s *morality* (“How moral of a person is Sarah?”), *trustworthiness* (“How trustworthy of a person is Sarah?”), *intelligence* (“How intelligent of a person is Sarah?”), and *athleticism* (“How athletic of a person is Sarah?”).

Secondary dependent measures. Next, we collected our secondary dependent measures: (i) sympathy towards the target and (ii) exploitability of the target. The order of these two pages was randomized between-subjects.

Sympathy. The set of sympathy question asked subjects in random order: “How sympathetic do you feel towards Sarah?”, “How bad do you feel for Sarah?”, and “How sorry for Sarah are you?”.

Exploitability. The set of exploitability question asked subjects in random order: “How trusting do you think Sarah is of others?”, “How easy do you think that it would be to exploit Sarah for one’s personal gain?”, “How naïve of a person do you think Sarah is?”, and “How likely do you think that people are to try to harm Sarah in the future?”.

Post-experimental survey

Finally, subjects completed a post-experimental survey in which they (i) answered a simple analogy question and wrote three sentences about their activities and plans for their day (questions that were designed to test English comprehension), and then (ii) answered a set of demographic and survey questions. In particular, subjects reported their age, gender, level of education, income, and country of residence.

5.16. Experiment 15 Pre-test

Design overview

Our Experiment 15 pre-test employed a two-by-two, between-subject design. As our first factor, we manipulated whether subjects were assigned to the *immoral* condition (in which the target was described as immoral) or the *moral* condition (in which the target was described as moral). As our second factor, we manipulated whether subjects were assigned to the *not upset* condition (in which no information was provided about whether the target was upset) or the *upset* condition (in which the target was described as being upset). We then randomly assigned subjects to one of the four possible conditions that resulted from crossing these manipulations,

and asked subjects to rate the likelihood that the target would engage in a set of candidate behaviors. We also randomized between-subjects whether the target character was male or female. Our Experiment 15 pre-test did not employ one of our standard vignettes, and in no conditions involved any kind of transgression; instead, we created a very brief and novel vignette for our Experiment 15 pre-test, reported below.

Vignette text

In the *moral, not upset* condition, the vignette read as follows: ***Sarah*** *is a politically moderate college student. In general, she is a pretty moral and trustworthy person.*

In the *moral, upset* condition, the vignette read as follows: ***Sarah*** *is a politically moderate college student. In general, she is a pretty moral and trustworthy person. Recently, something quite bad happened to her in her life that made her very upset.*

In the *immoral, not upset* condition, the vignette read as follows: ***Sarah*** *is a politically moderate college student. In general, she is not a very moral and trustworthy person.*

In the *immoral, upset* condition, the vignette read as follows: ***Sarah*** *is a politically moderate college student. In general, she is not a very moral and trustworthy person. Recently, something quite bad happened to her in her life that made her very upset.*

In the *male* version of the vignette, the name "Sarah" was replaced with "Sam" (and male pronouns were used).

Key dependent measures

On the page presenting this vignette, subjects made a series of behavior predictions about Sarah. Specifically, we presented each subject with a random set of six behavioral prediction questions (in random order) from the complete set of twelve questions listed below. All questions were answered on 1-9 Likert scales (Anchors: 1 – Not at all, 2, 3 – A little bit, 4, 5 – Moderately, 6, 7 – Quite a lot, 8, 9 – Extremely).

1. Sarah wants to buy a cup of coffee, and she could go to Starbucks. Alternatively, she could go to a local shop with similar prices and similar quality coffee, that donates a portion of its proceeds to charity. How likely is she to go to the local shop?
2. Sarah doesn't finish a meal she ordered at a restaurant, and doesn't intend to take the leftovers home with her. The waitress asks for her consent to give them to a homeless man who often comes by the restaurant. How likely is Sarah to say yes?
3. How likely is it that Sarah makes a habit of donating old clothes to goodwill instead of throwing them out?
4. How likely is it that Sarah makes a habit of always tipping 20% at restaurants?
5. How likely is it that Sarah makes a habit of always recycling?
6. How likely is it that Sarah always thanks the driver before getting off a bus or out of an uber?
7. How likely is that Sarah is always polite and gracious to people working in service (e.g., bartenders and waitstaff, salespeople, etc)?
8. Sarah's best friend Alicia's birthday is coming up soon. How likely is Sarah to put a ton of effort into planning a surprise party for Alicia?
9. Sarah reads in a newsletter that a local chapter of habitat for humanity was just founded, and they are looking for volunteers to help get things organized. How likely is Sarah to call them to sign up as a volunteer?

10. Sarah and her group of friends are chatting about their upcoming plans for spring break, and one girl complains that she can't afford a plane ticket home. How likely is Sarah to pull her aside later and offer to loan her money for a plane ticket?
11. Sarah is on the phone with her mom, who complains to her that her cooking thermometer recently broke. How likely is Sarah to surprise her mom by shipping her a new one from Amazon?
12. Sarah recently read an article about how the owner of her favorite sandwich shop is racist and discriminates when hiring. How likely is Sarah to stop going there for lunch?

Post-experimental survey

Finally, subjects completed a post-experimental survey in which they (i) answered a simple analogy question and wrote three sentences about their activities and plans for their day (questions that were designed to test English comprehension), and then (ii) answered a set of demographic and survey questions. In particular, subjects reported their age, gender, level of education, income, and country of residence.

5.17. Experiment 15

Design overview

Experiment 15 employed a two-condition, between-subject design in which subjects were assigned one of the following conditions: *neutral* (in which the target was a neutral character) and *standard victim* (in which the target behaved identically, but was victimized by a perpetrator). We also randomized between-subjects whether the target character (and all other characters in the vignette) was male or female. Experiment 15 used our iPad vignette, in which the relevant transgression is the theft of an iPad.

Vignette text

In all conditions, the vignette began as follows: ***Sarah*** *is a politically moderate college student. Last Wednesday, she and a few of her classmates were studying for a big upcoming exam in the common room of Sarah's dorm suite. At one point, one of Sarah's classmates, Gabrielle, asked to use Sarah's iPad to look something up. Later that day, Sarah relaxed by watching a TV show on her iPad, and then went out for the evening.*

This was the entire vignette in the *neutral* condition. In the *standard victim* condition, the vignette continued: *While Sarah was out, Gabrielle figured out how to enter her suite, and stole her iPad. When Sarah noticed that it was missing, she was very upset.*

In the *male* version of the vignette, the names "Sarah" and "Gabrielle" were replaced with "Sam" and "Gordon" respectively.

Measures

Subjects then answered a series of questions about characters in the vignette, spread out across several pages. All questions were answered on 1-9 Likert scales (Anchors: 1 – Not at all, 2, 3 – A little bit, 4, 5 – Moderately, 6, 7 – Quite a lot, 8, 9 – Extremely). On all pages, the (identical) vignette was presented before the questions. The first presentation of the vignette was paired with the first set of questions.

First key dependent measures. We began by measuring our first set of key dependent measures: a set of behavior predictions (four moral behaviors, two of which were pre-tested to be, and two of which were pre-tested not to be, less likely among upset actors).

Behavior predictions. Across the first two evaluation pages, subjects made a set of four predictions about the target's moral behavior. On the first page, subjects made two predictions about moral behaviors that were pre-tested *not* to be less likely among upset actors. On the second page, subjects made two predictions about moral behaviors that were pre-tested *to* be less likely among upset actors (on a second page page). We randomized the order of questions within pages.

The questions about moral behaviors that were pre-tested *not* to be less likely among upset actors asked subjects: "How likely is it that Sarah makes a habit of always recycling?" and "Sarah doesn't finish a meal she ordered at a restaurant, and doesn't intend to take the leftovers home with her. The waitress asks for her consent to give them to a homeless man who often comes by the restaurant. How likely is Sarah to say yes?".

The questions about moral behaviors that were pre-tested *to* be less likely among upset actors asked subjects: "Sarah is on the phone with her mom, who complains to her that her cooking thermometer recently broke. How likely is Sarah to surprise her mom by shipping her a new one from Amazon?" and "Sarah and her group of friends are chatting about their upcoming plans for spring break, and one girl complains that she can't afford a plane ticket home. How likely is Sarah to pull her aside later and offer to loan her money for a plane ticket?".

Other key dependent measure, and secondary dependent measure. Next, we collected our other key dependent measure (ratings of moral character), as well as our secondary dependent measure (ratings of the extent to which the target was upset). The order of these two pages was randomized between-subjects.

Moral character. On the page measuring moral character, subjects rated the target on two traits in random order. Subjects rated Sarah's *morality* ("How moral of a person is Sarah?") and *trustworthiness* ("How trustworthy of a person is Sarah?").

Upsetness. On the page measuring upsetness, subjects evaluated the extent to which the target was upset. Subjects answered two questions in random order: "To what extent do you think Sarah is currently feeling down about life, as if things aren't going her way?" and "How upset do you think Sarah is?".

Post-experimental survey

Finally, subjects completed a post-experimental survey in which they (i) answered a simple analogy question and wrote three sentences about their activities and plans for their day (questions that were designed to test English comprehension), and then (ii) answered a set of demographic and survey questions. In particular, subjects reported their age, gender, level of education, income, and country of residence.

5.18. Experiment 16

Design overview

Experiment 16 employed a two-condition, between-subject design in which subjects were assigned to one of the following conditions: *positively-valanced control* (in which the target was a neutral character, but was described as having positive nonmoral traits) and *standard victim* (in which the target was a neutral character, and was both described as having positive nonmoral

traits and as being morally good). We also randomized between-subjects whether the target character (and all other characters in the vignette) was male or female. Experiment 16 drew from the neutral condition of our iPad vignette, but in no conditions involved any kind of transgression.

Vignette text

In the *positively-valanced* condition, the vignette began as follows: ***Sarah*** *is a politically moderate college student. Her friends generally say that she is fun to be around, and has a good sense of humor.*

In the *direct morality* condition, the vignette instead began: ***Sarah*** *is a politically moderate college student. Her friends generally say that she is a moral and trustworthy person. They also say that she is fun to be around, and has a good sense of humor.*

In both conditions, the vignette then continued: *Last Wednesday, she and a few of her classmates were studying for a big upcoming exam in the common room of Sarah's dorm suite. At one point, one of Sarah's classmates, Gabrielle, asked to use Sarah's iPad to look something up. Later that day, Sarah relaxed by watching a TV show on her iPad, and then went out for the evening.*

Measures

Subjects then answered a series of questions about characters in the vignette, spread out across several pages. All questions were answered on 1-9 Likert scales (Anchors: 1 – Not at all, 2, 3 – A little bit, 4, 5 – Moderately, 6, 7 – Quite a lot, 8, 9 – Extremely). On all pages, the (identical) vignette was presented before the questions. The first presentation of the vignette was paired with the first set of questions.

Key dependent measures. We measured our key dependent measures: (i) ratings of moral character (morality and trustworthiness) as well as two nonmoral traits (intelligence and athleticism), and (ii) a set of behavior predictions (for four moral and four immoral behaviors).

Moral character and nonmoral traits. On the first evaluation page, subjects evaluated the target character, Sarah, on four traits in random order. Subjects rated Sarah's *morality* ("How moral of a person is Sarah?"), *trustworthiness* ("How trustworthy of a person is Sarah?"), *intelligence* ("How intelligent of a person is Sarah?"), and *athleticism* ("How athletic of a person is Sarah?").

Behavior predictions. Across the next two evaluation pages, subjects made a set of eight predictions about the target's moral behavior: four predictions about *moral* behavior (on one page) and four predictions about *immoral* behavior (on another page). The order of these pages was randomized between-subjects, as was the order of predictions within each page.

The questions about moral behavior asked subjects: "How likely do you think Sarah is to donate to charity?", "How likely do you think Sarah is to volunteer to tutor low-income middle school students for free?", "How likely do you think Sarah is to help a friend move to an off-campus apartment?", and "How likely do you think Sarah is to give leftover food to a homeless person?".

The questions about immoral behavior asked subjects: "How likely do you think Sarah is to flirt with others while in a committed relationship?", "How likely do you think Sarah is to intentionally 'forget' to pay a friend back?", "How likely do you think Sarah is to exclude or betray a friend in order to fit in with popular peers?", and "How likely do you think Sarah is to

spread private or mean gossip about others?”. The order of the questions was randomized between-subjects.

Post-experimental survey

Finally, subjects completed a post-experimental survey in which they (i) answered a simple analogy question and wrote three sentences about their activities and plans for their day (questions that were designed to test English comprehension), and then (ii) answered a set of demographic and survey questions. In particular, subjects reported their age, gender, level of education, income, and country of residence.
